# De novo-designed transmembrane proteins bind and regulate a cytokine receptor

**DOI:** 10.1038/s41589-024-01562-z

**Published:** 2024-03-13

**Authors:** Marco Mravic, Li He, Huong T. Kratochvil, Hailin Hu, Sarah E. Nick, Weiya Bai, Anne Edwards, Hyunil Jo, Yibing Wu, Daniel DiMaio, William F. DeGrado

**Affiliations:** 1 Department of Pharmaceutical Chemistry, School of Pharmacy, University of California, San Francisco, CA USA; 2 Department of Integrative Structural and Computational Biology, The Scripps Research Institute, La Jolla, CA USA; 3 Department of Genetics, Yale School of Medicine, New Haven, CT USA; 4 Department of Chemistry, University of North Carolina Chapel Hill, Chapel Hill, NC USA; 5 School of Medicine, Tsinghua University, Beijing, China; 6 Department of Therapeutic Radiology, Yale School of Medicine, New Haven, CT USA; 7 Department of Molecular Biophysics & Biochemistry, Yale University, New Haven, CT USA; 8 Yale Cancer Center, New Haven, CT USA

**Keywords:** Protein design, Membrane proteins, Cell signalling, Computational chemistry

## Abstract

Transmembrane (TM) domains as simple as a single span can perform complex biological functions using entirely lipid-embedded chemical features. Computational design has the potential to generate custom tool molecules directly targeting membrane proteins at their functional TM regions. Thus far, designed TM domain-targeting agents have been limited to mimicking the binding modes and motifs of natural TM interaction partners. Here, we demonstrate the design of de novo TM proteins targeting the erythropoietin receptor (EpoR) TM domain in a custom binding topology competitive with receptor homodimerization. The TM proteins expressed in mammalian cells complex with EpoR and inhibit erythropoietin-induced cell proliferation. In vitro, the synthetic TM domain complex outcompetes EpoR homodimerization. Structural characterization reveals that the complex involves the intended amino acids and agrees with our designed molecular model of antiparallel TM helices at 1:1 stoichiometry. Thus, membrane protein TM regions can now be targeted in custom-designed topologies.

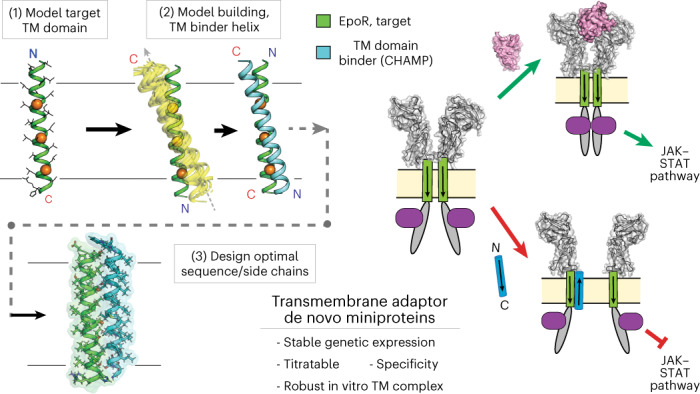

## Abstract (web summary)

The study demonstrates that specific recognition and custom binding geometries can be computationally encoded between protein spans within lipids through designing synthetic transmembrane proteins to functionally regulate a target cytokine receptor.

## Main

Protein transmembrane (TM) domains execute diverse and essential biological functions often through molecular features located deep within the bilayer hydrophobic region. It would be advantageous to have chemical biology tools targeting membrane proteins directly at lipid-embedded sites, allowing manipulation of their functional and structural states analogous to how antibodies and small molecules have been applied to water-exposed protein regions^1,2^.

Approaches for targeting TM regions exist but remain limited. Truncated peptides mimicking natural TM sequences can perturb the assembly of oligomeric complexes and multispanning proteins through competition for native inter-TM domain interactions^3–9^. However, the functional perturbations and membrane protein targets accessible by simple mimic molecules are limited. Likewise, as peptides, TM domain mimics have poor solubility and pharmacology, while as expressed proteins they can fail to properly traffic or insert, thereby restricting their molecular scope. Alternative approaches for TM polypeptide engineering include rational chemical derivatization^4,5,10^, computational design^11^ or screening expressed TM protein variant libraries^10,12,13^, each offering distinct benefits and limitations.

Theoretically, structure-based computational design can direct molecules to specific membrane protein regions with custom binding modes to afford the potential of stabilizing or recognizing distinct protein conformations. However, engineering TM protein complexes has major challenges and sparse precedent, namely due to due performance of models effectively estimating protein interaction energetics in lipids^14^, with noted recent developments^15,16^. The first proof-of-concept de novo design debuted the computed helical antimembrane protein (CHAMP) algorithm, wherein engineered TM peptides bound and selectively distinguished two highly similar single-pass integrin TM domains via association of mutual high-affinity TM GxxxG dimerization motifs presented on both the binder and target TM domains^11,17^. Computational design-informed mutagenesis was recently shown to be effective in improving the potency of natural TM domain mimic polypeptides inhibiting protein homo-oligomerization events^18,19^.

Until now, de novo computational designs have relied on encoding TM association via mimicking a single known, highly stable interaction motif. All designs (de novo or redesigns) have used a single topology: parallel TM helices with type I insertion^18,20,21^. Thus, 15 years after the initial proof of concept, both the binding modes and the spectrum of membrane protein targets have remained extremely limited; computational design of TM-targeting polypeptides is still far from routine.

**Fig. 1:**
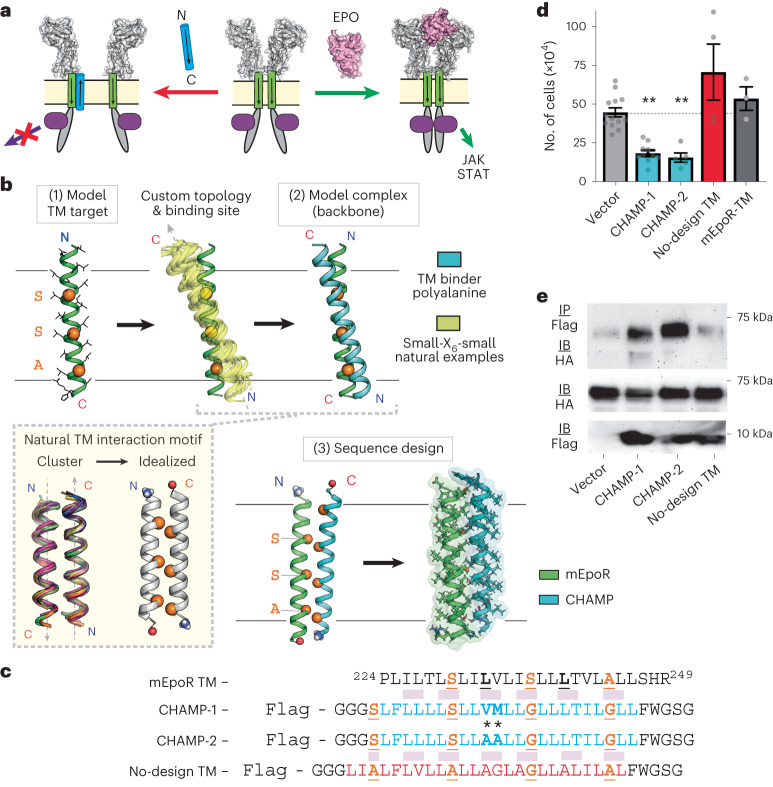
Design of TM proteins binding to mEpoR. **a**, CHAMPs (blue) targeting mEpoR’s TM domain (green) designed with antiparallel TM domain topology to competitively inhibit mEpoR homodimerization and impair cross-membrane activation of JAK−STAT signaling induced by EPO (pink) and JAK2 (purple). **b**, CHAMP algorithm. mEpoR’s TM helix (green) is modeled. A binding polyalanine CHAMP (blue) is positioned to mEpoR’s small-X_6_-small motif (orange, spheres) using a structural informatics approach modeling an idealized helix−helix geometry based on data mining natural examples of the TM motif (yellow, inset). De novo CHAMP sequences were designed with RosettaMP. **c**, TM sequences of mEpoR (leucine zipper repeat, bolded; small-X_6_-small, orange) and Flag-tagged synthetic TM constructs: no-design control (red) and CHAMPs (blue). Protein−protein interface, pink. Asterisks designate key differences. **d**, Mouse BaF3/mEpoR cell counts on day 6 in IL-3-free medium with 0.06 U ml^−1^ EPO stimulating proliferation when expressing the empty vector or synthetic TM domain constructs (vector, *n* = 13; CHAMP-1, *n* = 9 (*P* < 0.001); CHAMP-2, *n* = 5 (*P* < 0.001); no-design TM, *n* = 4; mEpoR-TM, *n* = 3; where *n* is the number of biological replicates). Error bars, standard error. Asterisks indicate *P* values reaching <0.05 from two-tailed unpaired Welch’s *t*-test compared with empty vector transduced cell counts. **e**, Flag-tagged TM proteins pull down HA-tagged mEpoR after co-expression in BaF3/mEpoR cells (representative of *n* = 3). IB, immunoblot; IP, immunoprecipitated. Source data

We sought to develop a generalizable workflow for computational design of TM adapter complexes with customizable geometry. We report several important technological advances in our design of de novo TM proteins binding the type I single-span mouse erythropoietin (EPO) receptor (mEpoR) to inhibit signaling. Our goal was to encode membrane-spanning interaction partners having type II insertion engaging mEpoR in an antiparallel TM helix topology, distinct from that of the parallel native mEpoR homodimer (Fig. 1a). Thus, our code does not use the receptor’s native TM interactions as a starting point. Second, our designed TM miniproteins can be stably expressed by using a titratable promoter to tune function in mammalian cells. Finally, we showed that the expressed TM proteins can inhibit a signal-amplifying receptor^22,23^, a strict functional requirement for our designs.

The designed TM proteins successfully associate with mEpoR and inhibit EPO−EpoR signaling in mammalian cells. Characterization in vitro and in live cells reveals that this complex’s overall structure agrees with the guiding design model of a heterodimeric antiparallel TM topology. This upgraded CHAMP software implementing RosettaMP^24^ can design synthetic TM proteins adopting a specific binding topology with a targeted membrane protein, holding promise for complex tasks of molecular recognition within lipid membranes.

## Results

### Designing TM domains to bind EpoR in a custom topology

EpoR is a prototypical cytokine receptor whose TM domain contributes to both receptor homodimerization^25–27^ and conformational coupling upon ligand binding^28,29^, activating the Janus kinase (JAK)−signal transducer and activator of transcription (STAT) pathway. The TM domains of mEpoR only weakly homodimerize^25–28^, mediating parallel self-interaction via a ‘serine−leucine zipper’ SXXLXXX seven-residue repeats (Fig. 1a–c)^30^. In model membranes, designed TM peptides hosting SXXLXXX sequence repeats spontaneously homodimerize, strongly favoring parallel TM helix geometry like that of mEpoR^31^. However, within multispanning proteins, SXXLXXX patterns mediate TM helix packing in both parallel and antiparallel geometries^30^. Thus, we hypothesized that mEpoR’s TM domain presents a malleable molecular surface susceptible to targeting via different geometries, a geometric specificity challenge we posed to our algorithm.

**Extended Data Fig. 1:**
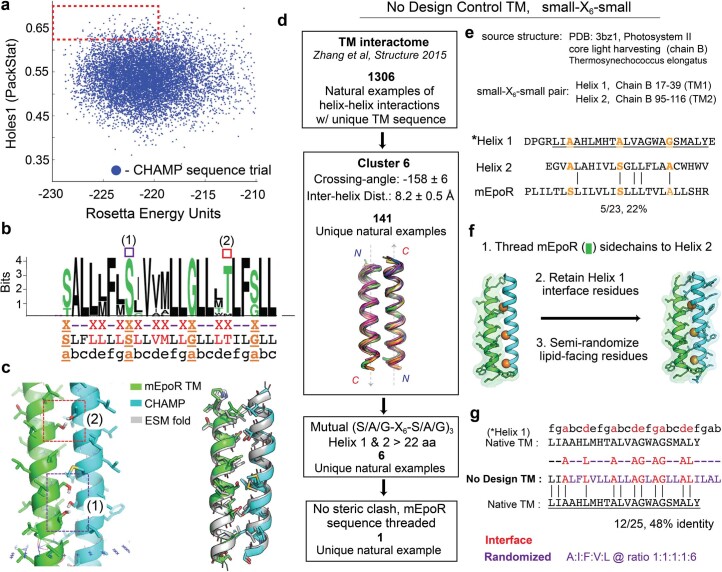
Protein design and ranking of output anti-mEpoR CHAMP sequences. **(a)** Scatter plot of each sequence design trajectory, showing each resulting model’s Rosetta energy and RosettaHoles1 interface packing voids ‘Packstat’ score^39^. Red dashed box represents designed proteins with Rosetta energy less than the mean and top 10% of Packstat. **(b)** Sequence logo CHAMP designs. Below denotes positions designed or fixed. Black hyphen, lipid-facing position fixed to an apolar residues (AFILV); red X, any lipid-friendly residue (GATSVLIFM); orange X, small-X_6_-small positions (GAS). Residues in hydrogen bonds with mEpoR are denoted as 1 and 2 with purple and red squares, respectively. **(c)** Left, model of CHAMP-1/mEpoR TM domain complex of antiparallel helices (green, mEpoR; cyan, CHAMP). Two key hydrogen inter-helical bond groups denoted in purple and red squares, numbered as in (b). Right, ESMfold *ab initio* predicted model of the complex (gray) overlaid, agreeing in helix register and sidechain interactions. **(d)** Rules-based selection of the ‘No Design’ control TM sequence based on TM domain helix-helix pairs filtered from a database of antiparallel left-handed close-packing having the small-X_6_-small consensus sequence^32^. More details in Methods. The mEpoR sequence was threaded onto one helix aligned with the small-X_6_-small motif and assessed for sidechain steric clashes with the native adjacent TM domain sidechains. Only in 1 structural case was compatible with mEpoR’s sequence modeled. **(e)** The source structure, Photosystem II light harvesting complex (PDB:3bz1, chain B), comprised of TM 1 and 2. mEpoR could be threaded onto TM 2, which has only 22% sequence identity. Orange, small-X_6_-small positions. **(f)** Schematic of selecting the ‘No Design’ sequence based Photosystem II TM domain 1 interface residues. Orange spheres, small-X_6_-small positions. **(g)** Final sequence selection of the ‘No Design’ sequence (interface residues in red retained, purple ransomized lipid-facing positions randomized) and comparison to the Photosystem II source TM domain.

mEpoR’s serine−leucine zipper also encompasses the S230-S237-A244 seven-residue pattern of small residues repeated every other helix turn. This small-X_6_-small pattern was queried against our published structural database and globally clustered TM helix−helix interaction geometries^32,33^ and was found to be associated with a common structural topology of tightly packing antiparallel TM helices with a shallow left-handed crossing. The consensus small-X_6_-small amino acids directly line these interfaces, allowing for close approach to the partner helix’s backbone. From >100 nonredundant natural examples of this two-helix TM interaction geometry, we generated idealized backbone coordinates as the template for protein−protein interaction design^34^: interhelical distance = 8.1 Å, crossing angle = −175 degrees, z offset = 2 Å (Fig. 1b and Extended Data Fig. 1). There is little experimental evidence to date concerning the sequence−structure principles for how to encode the antiparallel interaction of small-X_6_-small TM domains^31,35,36^, contrasting with previous designs that relied heavily on receptor mimicry^19^ or well-known sequence motifs for encoding TM interactions, for example, GxxxG^11,17^. Thus, we tested the ability of the data-driven modeling approach to effectively encode a CHAMP sequence de novo through specific complementary interactions with the target’s unique TM molecular surface in defining the desired complex.

First, the target TM domain (here, mEpoR) is modeled as an ideal α-helix embedded in an implicit membrane at an energy-optimized depth and orientation^37^. Second, a polyalanine backbone model of the putative CHAMP binding partner is built in a favorable helix−helix conformation with the target, precisely positioned relative to mEpoR’s small-X_6_-small pattern using the aforementioned data-mined idealized antiparallel topology (Fig. 1b). Next, a flexible-backbone side chain packing routine implemented with RosettaMP^38^ designs the CHAMP sequence, optimizing interactions with mEpoR’s TM domain. Of the 24 embedded CHAMP residues, 13 were automatically designated as ‘potentially interfacial’. The remaining ‘lipid-facing’ residues were semi-randomly assigned an apolar identity (isoleucine, leucine, valine, phenylalanine) fixed throughout the design. The four small-X_6_-small positions were limited to glycine, serine and alanine identities given the bioinformatics data, while the nine remaining interfacial residues sampled a limited lipid-friendly alphabet (glycine, alanine, threonine, serine, valine, leucine, isoleucine, phenylalanine or methionine). The sequence profile of the CHAMP designs is shown in Supplementary Fig. 1.

The critical final step was ranking and selecting the designed sequences according to the theoretical stability of the mEpoR-bound complex. Given the documented poor accuracy of interaction energies predicted by RosettaMP^14^, we instead ranked the models primarily based on the lack of side chain packing voids—a model quality metric commonplace in soluble protein structure prediction and design^39^. We identified design models in the top 10% of Rosetta’s ‘PackStat’ score, whose sequence profile and scores are displayed in Extended Data Fig. 1a,b, and then reduced the selection to two prominent sequences by sequence clustering (Supplementary Fig. 1). This completed the rule-based selection of the highly similar anti-mEpoR CHAMP-1 and CHAMP-2 sequences (Fig. 1c). Orthogonal ab initio prediction of mEpoR−CHAMP complexes by ESMfold^40^ yielded close-packed models within 0.9-Å backbone root mean square deviation of our designs (Extended Data Fig. 1c), suggesting that this close-packed topology is the lowest energy structure for these sequences. We also tested a ‘no-design’ control TM protein with a database-derived sequence (Extended Data Fig. 1d–g) to probe the inherent binding specificity for mEpoR’s TM domain encoded in a generic small-X_6_-small repeat.

Specific CHAMP algorithm adjustments^11^ included (1) implementation in RosettaMP to increase user accessibility; (2) ranking designs on interface packing over Rosetta energy scores; and (3) using an idealized structural bioinformatics-derived molecular model for the CHAMP−mEpoR complex, versus natural templates. Finally, human visual evaluation was cited as critical in past designs^11,19^ but introduces user disparities and limits reproducibility. Our adaptations automate model building, design and final sequence ranked selection, facilitating broader community use.

### CHAMP TM complex with mEpoR inhibits EPO-induced growth

**Extended Data Fig. 2:**
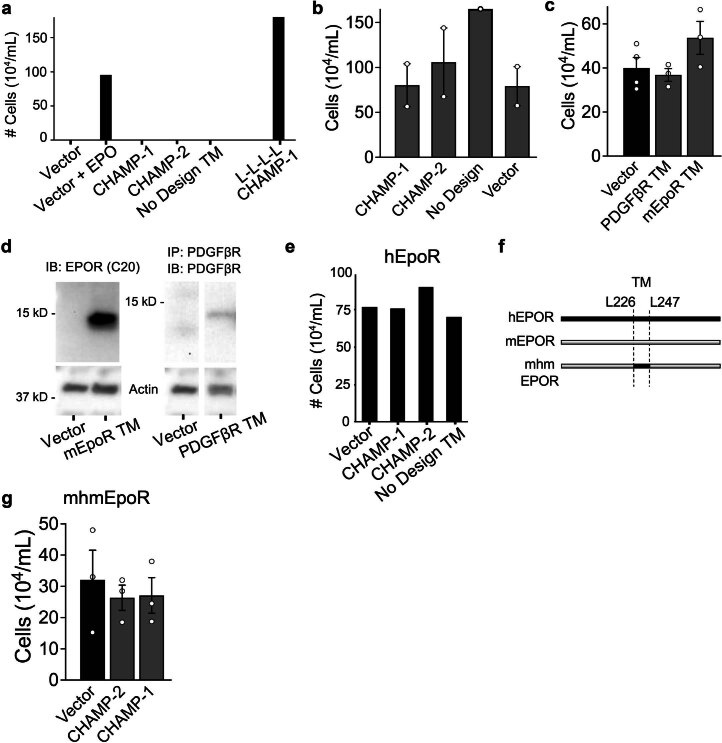
Effects of exogenous synthetic TM proteins on EpoR expressing BaF3 cells. **(a)** BaF3/mEpoR cells transduced with empty vector or a TM proteins (CHAMP-1, CHAMP-1, No Design TM, or CHAMP-1 mutant ‘L-L-L-L’) grown in media not treated with IL-3 and EPO. The number of live cells at day 4 is shown for a single experiment (n = 1). EPO-treated cells transduced only empty vector are the positive control for EPOR-dependent proliferation. **(b)** Mean cell counts on day 4 of BaF3/mEpoR cell proliferating in medium supplemented with IL-3 (5% WEHI-conditioned medium as source) stably expressing empty vector (n = 2), CHAMP-1 (n = 2), CHAMP-1 (n = 2), or No Design TM (n = 1) with bars representing standard error. **(c)** Mean live cell count of BaF3/mEpoR cells expressing empty vector, a short mEpoR TM domain protein construct, or a previous published PDGFβR TM domain construct (Ref. ^41^) after 8-day incubation in medium supplemented with 0.06 U/mL EPO (n = 3). Error bars as standard error. **(d)** Upper, western blots of BaF3/mEpoR cells stably expressing the mEpoR TM construct (C20 cytoplasmic epitope antibody) and PDGFβR TM construct. The PDGFβR TM construct was first immunoprecipitated to increase concentration then immunoblotted using PDGFβR-targeted rabbit anti-serum (C-terminal epitope) as in Ref. ^41^. Performed once (n = 1). **(e)** BaF3/hEPOR cells expressing empty vector, CHAMP-1, CHAMP-1, or No Design TM were incubated for four days in medium supplemented with 0.06 U/mL EPO. The number of live cells is shown for a single experiment (n = 1). **(f)** mhmEpoR construct having the cytoplasmic and extracellular domain sequences of mEpoR but the TM domain of hEpoR. **(g)** Mean live cell count of BaF3/mhmEpoR cells expressing empty vector, CHAMP-1, CHAMP-1, or No Design TM after 8-day incubation with 0.06 U/mL EPO (n = 3). Error bars show standard error. Source data

To test the activity of these designed sequences, retroviral transduction was used to stably express Flag-tagged CHAMP TM proteins in mouse BaF3 cells engineered to express mEpoR (BaF3/mEpoR cells), which lack endogenous EpoR (Fig. 1c–e). Proliferation of BaF3/mEpoR cells can be stimulated by the growth factors interleukin-3 (IL-3) (EpoR-independent) or EPO (EpoR-dependent). CHAMP protein expression did not induce proliferation in the absence of IL-3 and EPO (Extended Data Fig. 2a), indicating a lack of EPO-independent mEpoR activation. Additionally, IL-3-induced proliferation was not reduced by the designed TM proteins (Extended Data Fig. 2b), showing that their expression is not cytotoxic.

We next assayed whether the designed TM proteins impair cell proliferation due to EPO-induced EpoR activation. Over 8 days, EPO-treated BaF3/mEpoR cells expressing CHAMP-1 and CHAMP-2 exhibited significantly reduced proliferation with final cell counts reaching 38% ± 5% and 40% ± 6% (average ± s.e.m., *n* = 6), respectively, versus cells transduced with an empty vector (*P* < 0.05). (Fig. 1d). EPO-treated BaF3/mEpoR cells expressing the no-design control small-X_6_-small-containing TM protein, a type I mEpoR TM domain mimic protein or an unrelated mouse platelet-derived growth factor β receptor (PDGFβR) TM domain protein construct^41^ did not show inhibited proliferation (Fig. 1d, Extended Data Fig. 2c,d and Supplementary Table 1). Thus, only the designed CHAMP proteins exerted dominant-negative inhibition on mEpoR-dependent proliferation induced by EPO. When human EpoR (hEpoR) was expressed instead of mEpoR, CHAMP protein expression did not hamper EPO-stimulated proliferation (Extended Data Fig. 2e). Similarly, CHAMP-1 and CHAMP-2 expression failed to inhibit EPO-stimulated proliferation in cells expressing the ‘mhm-EpoR’ chimera (which consists of mEpoR’s water-soluble domains but the TM domain from hEpoR; Extended Data Fig. 2f,g). This specificity for the mEpoR TM domain is remarkable, given that hEpoR differs from mEpoR by only three mid-spanning residues.

We next used co-immunoprecipitation to test whether the TM proteins physically associate with mEpoR. Detergent lysates of BaF3/mEpoR cells expressing CHAMPs or the no design control proteins (Flag tagged at the N terminus) were immunoprecipitated with an anti-Flag antibody followed by immunoblotting with an antihemagglutinin (HA) antibody recognizing HA-tagged mEpoR. The anti-Flag antibody pulled down mEpoR only from cells expressing CHAMP-1 and CHAMP-2 (Fig. 1e), indicating that these proteins formed a stable complex with mEpoR. The small TM proteins did not affect expression levels of mEpoR.

### Topology of the CHAMP-1/mEpoR TM complex matches the design

**Fig. 2:**
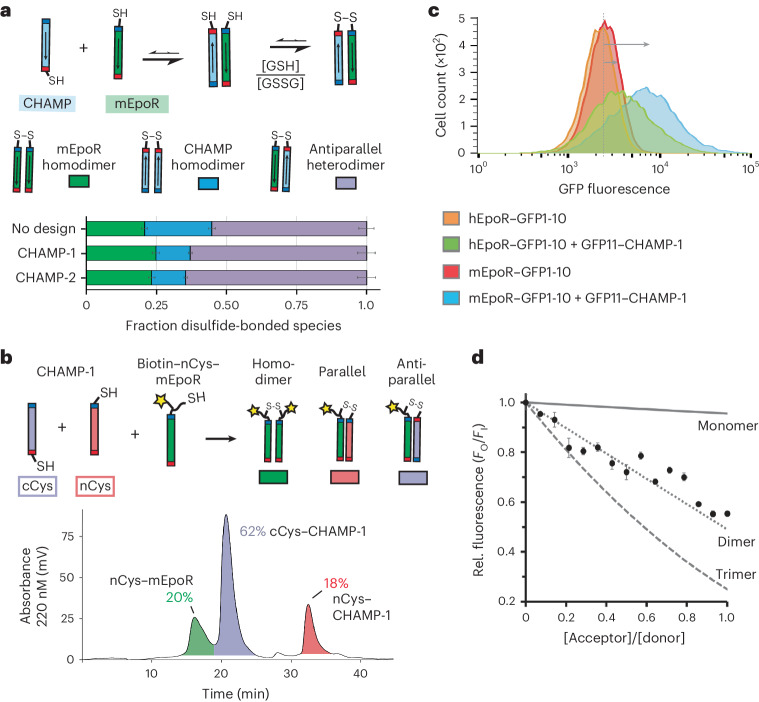
De novo TM domains target mEpoR in the intended dimeric antiparallel type II topology. **a**, Top, equilibrium thiol-disulfide exchange wherein mEpoR-TM peptide with an N-terminal cysteine (green) and each designed TM peptide with a C-terminal cysteine (blue) were reversibly oxidized by mixed glutathione in POPC small unilamellar vesicles (1:50 peptide to lipid ratio). Middle, legend of disulfide-bonded species: mEpoR homodimer (green), antiparallel CHAMP−mEpoR heterodimer (purple), TM design homodimer (blue). Bottom, molar fractions of covalent dimer species (parts of a whole plot) quantified by HPLC (Supplementary Fig. 1; *n* = 3). Blue, N terminus; red, C terminus. **b**, Competitive thiol-disulfide exchange (detailed scheme in Extended Data Fig. 3). Top, legend of peptides. Biotin−nCys−mEpoR-TM peptide (green) reconstituted with both nCys−CHAMP-1 (red) and cCys−CHAMP-1 (purple) was reversibly oxidized all together in C14-Betaine micelles, testing the preference for parallel and antiparallel dimeric species, respectively. Bottom, covalent species captured (streptavidin beads) were reduced, eluted as monomeric peptides and quantified by HPLC (representative of *n* = 3). **c**, Split GFP complementation assay and flow cytometry of BaF3 cells expressing mEpoR−GFP1-10 or hEpoR−GFP1-10 in the presence or absence of co-expressed N-terminal GFP11−CHAMP-1 fusion (representative of *n* = 3 trials). GFP reconstitution indicates GFP11 cytoplasmic localization and CHAMP-1 type II TM orientation. **d**, mEpoR:CHAMP-1 stoichiometry. Dots represent the mean relative (Rel.) donor fluorescence emission quenching of 1.5 µM 7-diethylamino-4-methylcoumerin-labeled mEpoR-TM peptide titrated with fluorescein-labeled CHAMP-1 in C14B at a fixed equimolar total peptide concentration (*n* = 3; bars, standard error). Theoretical FRET curves overlaid for monomer, dimer (1:1) and trimer (2:1) assemblies.

**Extended Data Fig. 3:**
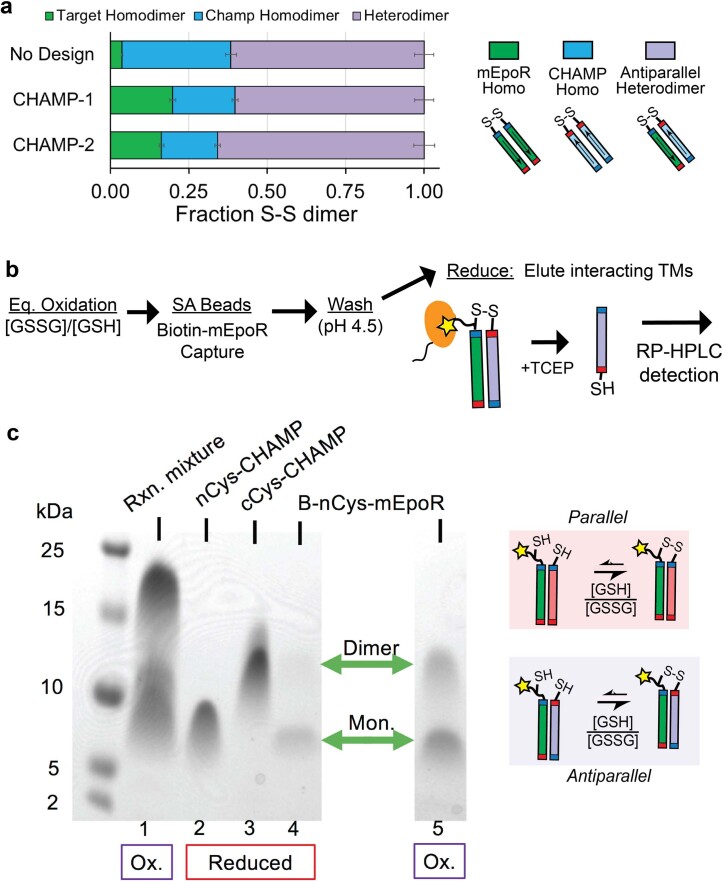
Schematic and SDS-PAGE of the three peptide thiol-disulfide exchange with biotin capture. **(a)** Thio-disulfide exchange experiment Fig. 2a repeated with peptide reconstituted in dodecylphosphocholine (DPC) detergent micelles, showing similar results. Bars represent fraction of total integrated peak area of each species’ peak in the HPLC UV chromatogram, representative of 3 experiments; error bars bootstrapped from the 5% curve fitting error to HPLC UV peaks. **(b)** Workflow for equilibrium thiol-disulfide exchange modified for biotin capture and isolation of only mEpoR-containing disulfide bonded TM dimers, followed by reduction and quantification of the interacting TM helices by reverse phase high-performance liquid chromatography (RP-HPLC). SA, Streptavidin; GSSG, GSH, oxidized and reduced glutathione, respectively; Eq., equilibrium. **(c)** Sodium dodecyl sulfate polyacrylamide gel electrophoresis (SDS-PAGE) of equilibrated oxidized TM peptides in 20 mM m*yristyl sulfobetaine* (C14B) micelles (lanes 1, 5), or purified peptides (lanes 2, 3, 4) reduced in 10 molar equivalents of tris(2-carboxyethyl)phosphine (TCEP), performed once (n = 1). *Lane 1*, mixture of 100 µM Biotinylated nCys-mEpoR with 4-fold molar excess of CHAMP – 2 equivalents of each nCys-CHAMP1 and cCys-CHAMP1 V2 oxidized overnight at a molar ratio of oxidized to reduced glutathione of 0.2. *Lane* 5, 100 µM Biotinylated nCys-mEpoR alone oxidized under the same conditions. Green arrows denote mEpoR monomer and dimer bands. Ox., oxidizing conditions; Mon., Monomer; kDa, kilodaltons; Rxn., Reaction. Source data

We next characterized whether the complex formed with mEpoR’s TM domain in vitro conforms to the intended structure designed computationally: TM helices bound in an antiparallel geometry at 1:1 stoichiometry, which outcompete mEpoR TM homodimerization. First, we tested the relative association of mEpoR’s TM domain and the designed TM sequences as cysteine-containing synthetic peptides in model membranes through an established equilibrium thiol-disulfide exchange assay monitoring relative cysteine reactivity due to noncovalent complex formation (Fig. 2a)^42,43^. Each of the designed TM peptides (with a C-terminal cysteine) was reconstituted with an mEpoR TM domain peptide (with an N-terminal cysteine, mEpoR-TM) in basic buffered solution at a 1:100 peptide to detergent or lipid molar ratio. Following glutathione-assisted reversible oxidation, all the disulfide-bonded dimer species were separated and quantified by reverse-phase high-performance liquid chromatography (RP-HPLC) (Supplementary Fig. 1). In either dodecylphosphocholine (DPC) micelles (Extended Data Fig. 3a) or 1-palmitoyl-2-oleoyl-*sn*-glycero-3-phosphocholine (POPC) small unilamellar vesicles (Fig. 2a), the CHAMP-1, CHAMP-2 and no-design control peptides showed a strong nonrandom preference to form N-to-C disulfide-bonded heterodimers with mEpoR-TM, at 2.5-fold- to 16-fold-higher propensity than mEpoR homodimer formation. Thus, the three de novo small-X_6_-small TM peptides form stable antiparallel complexes in vitro with mEpoR-TM that outcompete its parallel self-interaction.

Then, we performed a second thiol-disulfide exchange experiment (workflow in Extended Data Fig. 3b,c) to test whether CHAMP-1 prefers parallel versus antiparallel helix orientation in complex with mEpoR (not directly probed in the previous assay). We measured whether CHAMP-1 peptide with an N-terminal or C-terminal cysteine (nCys−CHAMP-1, cCys−CHAMP-1) more readily forms disulfide bonds with biotinylated mEpoR-TM peptide with an N-terminal cysteine (biotin−nCys−mEpoR) in detergent solution (Fig. 2b). A three-peptide mixture of a 2:2:1 molar ratio of nCys−CHAMP-1, cCys−CHAMP-1 and nCys−mEpoR was first reconstituted in micellar solution at a 40:1 molar ratio of myristyl sulfobetaine (C14B) detergent to total peptide and then subjected to reversible glutathione-assisted oxidation followed by low -pH quenching. Next, biotin−nCys−mEpoR-containing species were bound to streptavidin beads, also capturing covalently disulfide-bonded TM peptides. After extensive washing, the beads were treated with a reducing agent to elute the TM peptides captured via disulfide, which were then collected and quantified as monomeric species by RP-HPLC. cCys-CHAMP-1 represented >60% of the total peptide captured by disulfide bond formation to biotin−nCys−mEpoR (Fig. 2b), which is more than threefold more than either nCys−CHAMP-1 or biotin-nCys−mEpoR. Thus, in vitro, the TM helices of the CHAMP-1−mEpoR complex showed a strong preference for antiparallel topology.

**Extended Data Fig. 4:**
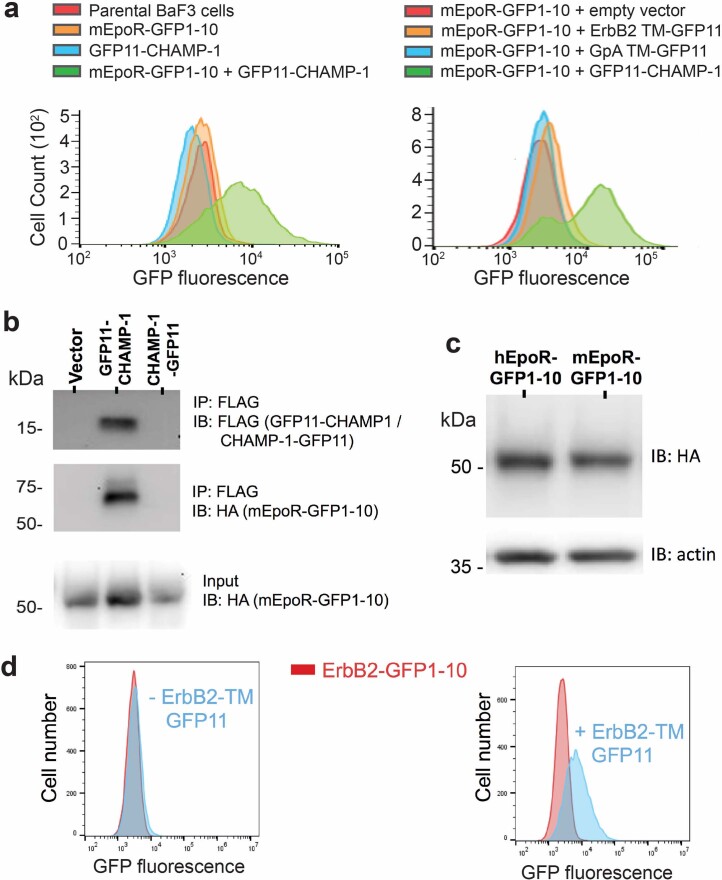
Control split GFP protein complement experiments. **(a)** Left, flow cytometry histogram showing fluorescence of parental BaF3 cells and BaF3 cells expressing mEpoR-GFP1-10 and GFP11-CHAMP-1 together or separately. Right, flow cytometry histogram showing fluorescence of BaF3/mEpoR-GFP1-10 cells co-expressing empty vector, GFP11-CHAMP-1, a GlycophorinA (GpA) TM domain construct fused at its c-terminus to GFP11, or ErbB2 TM domain construct fused to GFP11 at its c-terminus (Supplementary Table 1). **(b)** Extracts were prepared from mEpoR-GFP1-10 cells expressing empty vector or FLAG-tagged CHAMP-1 with a fused N-terminal GFP11 (GFP11-CHAMP-1) or a fused C-terminal GFP11 (CHAMP-1-GFP11). Extracts were subjected to SDS-PAGE directly (input) or after immunoprecipitation with anti-FLAG antibody, which recognizes both FLAG-tagged CHAMP-1 proteins. Performed once (n = 1). Blots were probed with antibodies that recognize FLAG to detect the CHAMP-1 proteins or HA to detect HA-tagged mEpoR-GFP1-10. **(c**) Extracts from cells expressing either the mEpoR or the hEpoR C-terminally fused to GFP1-10 were subjected to SDS-PAGE and immunoblotted with antibodies that recognize HA fused to EpoR or actin as a loading control. Performed once (n = 1). The numbers below the lanes are the relative expression of the EpoR normalized to actin. **(d)** BaF3 (tTa) cells expressing ErbB2 C-terminally fused to GFP1-10 transduced with empty pTight vector or pTight containing the TM domain of ErbB2 fused to GFP11 (ErbB2TM-GFP11). Flow cytometry was performed on cells incubated in 1 nM doxycycline to repress ErbB2TM-GFP11 (left panel) or in 1 pM doxycycline expressing ErbB2TM-GFP11 (right panel). Source data

To assess the TM topology and antiparallel TM orientation of CHAMP-1 with mEpoR in a cellular context, we used split green fluorescent protein (GFP) complementation in BaF3 cells. Fluorescence is generated when two nonfluorescent fragments of GFP (GFP1-10 and GFP11) are in the same cellular compartment in proper proximity and orientation for stable reconstitution (Fig. 2c)^44^. Control experiments with hEpoR−GFP1-10 or mEpoR−GFP1-10 fusions (via a short flexible linker replacing EpoR’s C-terminal cytoplasmic domain) expressed alone in BaF3 cells showed low background cellular mean fluorescence intensity (MFI) (2.5 × 10^3^) (Fig. 2c). We then co-expressed Flag-tagged CHAMP-1 with GFP11 fused to the N or C terminus with hEpoR−GFP1-10 or mEpoR−GFP1-10. Co-expression of the N-terminal GFP-11−CHAMP-1 fusion with mEpoR−GFP1-10 led to a more than fourfold higher MFI than that of cells expressing mEpoR−GFP1-10 alone or the CHAMP-1 fusion alone (Fig. 2c and Extended Data Fig. 4a), indicating successful cytoplasmic GFP11 localization and complex formation. We also confirmed complex formation between mEpoR−GFP1-10 and GFP11−CHAMP-1 by co-immunoprecipitation (Extended Data Fig. 4b). Co-expression of GFP-11−CHAMP-1 with hEpoR−GFP1-10 yielded a smaller twofold increase in MFI (Fig. 2c), despite mEpoR−GFP1-10 and hEpoR−GFP1-10 being expressed at similar levels (Extended Data Fig. 4c), consistent with the preference of CHAMP-1 for mEpoR versus hEpoR in the growth inhibition assay. In mEpoR−GFP1-10 expressing cells, fluorescence was not increased by expression of alternative noninteracting TM domains (from glycophorin A or ErbB2) fused at their cytoplasmic end to GFP11 (Extended Data Fig. 4a), although the ErbB2 TM domain fusion could complement ErbB2−GFP1-10 as expected (Extended Data Fig. 4d). The C-terminal CHAMP-1−GFP-11 fusion was not expressed at a detectable level and did not increase fluorescence (Extended Data Fig. 4a,b). These results indicate that a substantial population of the de novo CHAMP-1 adopts type II TM insertion (cytoplasmic N terminus) and antiparallel helix interaction with mEpoR−GFP1-10 in mammalian cell membranes, as intended in silico.

**Extended Data Fig. 5:**
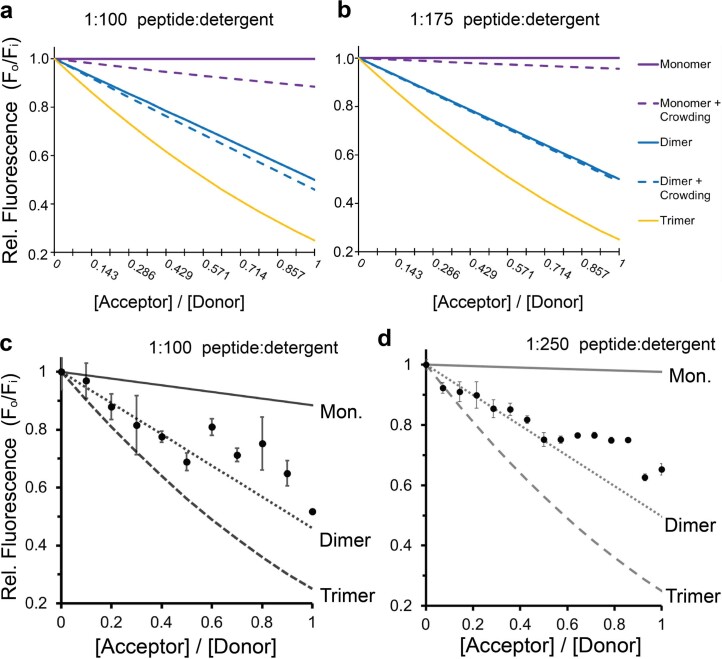
Fluorescence quenching of labeled-mEpoR by CHAMP-1 in C14B micelles at different peptide:detergent ratios and theoretical FRET from non-specific peptide crowding effects. **(a)** Theoretical FRET curves expected for monomeric (Mon.), dimeric, and trimeric complexes are plotted as solid lines. Dotted lines represent FRET curves of each potential oligomerization state also including the micelle crowding factor, which accounts for the contribution to quenching from non-specific co-habitation of donor and acceptor TM peptides in the same micelle, which we modeled as a Poission distribution dependent on the peptide to detergent molar ratio and the detergent’s aggregation number (molecules per micelle). The expected FRET curves with the micelle crowding factor at 1:100 peptide to detergent ratio mole ratio is displayed, given the estimated aggegration number for C14B of 90. **(b)** Theoretical FRET curves with the micelle crowding factor 1:175 peptide: C14B molar ratio, plotted as in panel **a**. **(c)** Dots represent mean relative (Rel.) 460 nm fluorescence emission of 1.5 µM 7-diethylamino-4-methylcoumerin-labeled (DACM-labelled) mEpoR TM peptide (n = 3, independent samples; standard error bars) decaying linearly due to FRET quenching as fluorescein-labeled CHAMP-1 is titrated. Samples were reconstituted in C14-betaine with a detergent to total peptide ratio of 100:1 (1.1 micelles per total peptide) in 50 mM Tris-HCl pH 8, 100 mM NaCl, 0.5 mM EDTA, 5 mM TCEP buffer at fixed equimolar total peptide concentration across the donor titration. **(d)** Mean fluorescence emission of analogous experiment and plot details as in panel c, but reconstituted at a detergent to total peptide ratio of 250:1 (2.8 micelles per total peptide).

Finally, we used a fluorescence resonance energy transfer (FRET)-based fluorescence quenching method^45,46^ to determine the stoichiometry of the CHAMP-1−mEpoR complex in detergent micelles. Increasing molar ratios of fluorescein-5-maleimide-labeled CHAMP-1 (acceptor) peptide to diethylamino-4-methylcoumarin-3-maleimide-labeled mEpoR (donor) peptide were reconstituted in C14B micelles (constant 180:1 detergent to total peptide ratio). Quenching of donor emission was observed, decaying linearly to half-maximum intensity at a 1:1 acceptor to donor ratio (Fig. 2d). Comparing this FRET behavior to theory (Extended Data Fig. 5a,b)^46,47^ suggests that the TM peptides form a nearly full-occupancy complex of 1:1 stoichiometry under these conditions (0.3% mol fraction CHAMP-1 in detergent). Concentrating the TM peptides by decreasing the detergent to peptide ratio (100:1) did not cause additional quenching, but further dilution of the complex (250:1) slightly reduced fluorescence decay, linearly increasing the monomer fraction (Extended Data Fig. 5c,d). These data indicate that the fluorophore-labeled CHAMP-1−mEpoR-TM complex is hetero-dimeric as designed.

### CHAMP-1 inhibits EpoR signaling in a sequence-dependent manner

**Fig. 3:**
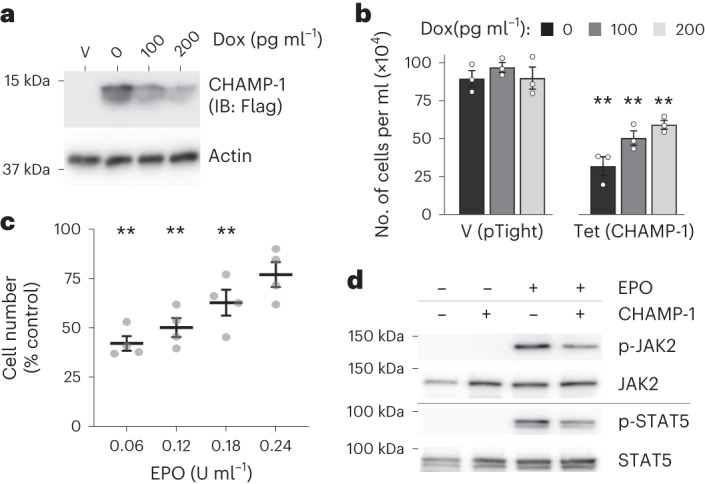
Expression of designed TM protein CHAMP-1 inhibits the EPO−mEpoR signaling cascade. **a**, Dox repression of Flag-tagged CHAMP-1 expression levels from a tetracycline-responsive promoter in BaF3/mEpoR cells expressing the tTA tetracycline transactivator, measured by SDS−PAGE and anti-Flag immunoblot of cells treated with Dox titration. Performed once (*n* = 1). Actin is a loading control. V, empty vector. **b**, Mean number of cells after growth in medium supplemented with 0.06 U ml^−1^ EPO treated with 0, 100 or 200 pg ml^−1^ Dox for cells expressing CHAMP-1 compared to empty vector (pTight) transduced cells (from left to right, *P* = 0.002, 0.002 and 0.037, respectively; *n* = 3). Bars, standard error; *P*-values from unpaired Welch’s *t*-test. **c**, Mean number of BaF3/mEpoR cells expressing CHAMP-1 after incubation for 6 days in medium containing EPO concentrations of 0.06, 0.12, 0.18 and 0.24 U ml^−1^ (from left to right, *P* = 0.033, 0.025, 0.003 and 0.08, respectively; unpaired two-tailed Welch’s *t*-test; *n* = 4 replicate experiments) relative to the number of cells not transduced with CHAMP-1 similarly treated with EPO. **d**, BaF3/mEpoR cell extracts having CHAMP-1 expression and/or 1 U ml^−1^ EPO treatment for 10 min subjected to SDS−PAGE and immunoblotted with antibodies recognizing either phosphorylated or total JAK2 or STAT5 (*n* = 1). **P < 0.05. Source data

For CHAMP-1, we investigated the sequence features and the mechanism driving its function. Expression under a titratable doxycycline (Dox)-repressible promoter in BaF3/mEpoR cells showed that inhibition of EPO-induced proliferation was dose-dependent and tunable by CHAMP expression levels (Fig. 3a,b). Likewise, the inhibitory effect was negatively correlated with the concentration of stimulatory EPO (0 to 0.24 U ml^−1^), as expected (Fig. 3c). Phosphorylation-specific immunoblotting showed that EPO-stimulated tyrosine phosphorylation of JAK2 and STAT5, downstream effectors of EpoR, was reduced by CHAMP-1 expression (Fig. 3d), indicating that CHAMP-1 inhibits the EPO−mEpoR cross-membrane signaling axis.

**Fig. 4:**
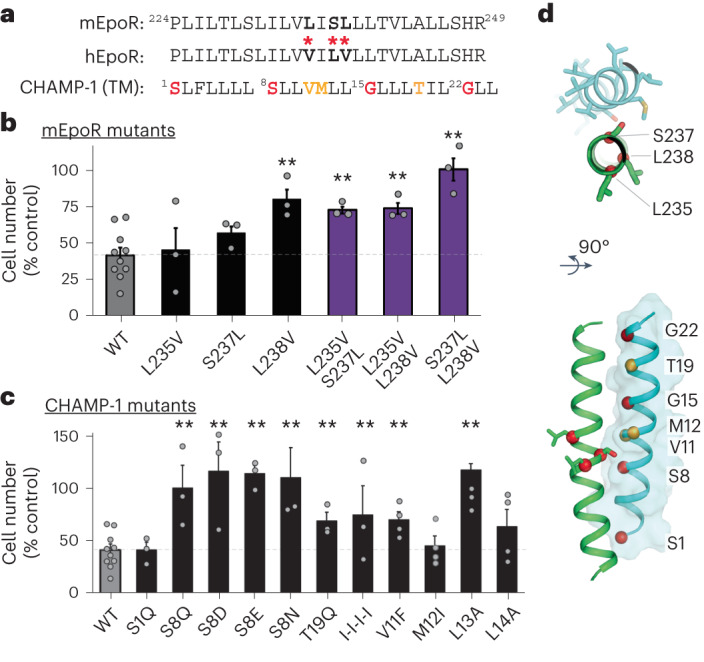
Sequence-specific interaction between mEpoR and CHAMP-1. **a**, Wild-type sequences for core TM regions of mEpoR, hEpoR and CHAMP-1. Dissimilar residues between mEpoR and hEpoR are indicated by bold letters and red asterisks. Mutated CHAMP-1 residues expected to be in contact with mEpoR from the design model include those in the small-X_6_-small repeat S1-S8-G15-G22 (red) and other amino acids at intermediate helix turns (orange). **b**, BaF3 cells stably expressing mEpoR mutants with single or double mEpoR-to-hEpoR amino acid substitutions co-expressed with wild-type CHAMP-1 were cultured in medium supplemented with 0.06 U ml^−1^ EPO. Day 6 mean cell counts are shown as a percentage, relative to the number of cells of wild-type BaF3/mEpoR/ cells with EPO-stimulation and CHAMP-1 expression (*n* = 3; bars, standard error) with significant increases denoted by asterisks; one-tailed Student’s *t*-test. *P*-values: L235V, 0.390; S237L, 0.082; L238V, 0.002; L235V/S237L, 0.005; L235V/L238V, 0.005; S237L/L238V, <0.001. **c**, BaF3/mEpoR cells stably expressing CHAMP-1 mutants were cultured in medium supplemented with 0.06 U ml^−1^ EPO. Mean cell counts at day 6 are shown normalized to the number of cells in the absence of CHAMP-1 expression (*n* = 3; bars, standard error). Asterisks denote a significant decrease in inhibitory potency relative to wild-type CHAMP-1 (increase in cell count, normalized to vector-only control) using a one-tailed Student’s *t*-test, *P*-values: S1Q, 0.488; S8Q, 0.001; S8D, <0.001; S8E, <0.001; S8N, 0.001; T19Q, 0.013; I-I-I-I, 0.035; V11F, 0.23; M12I, 0.454; L13A, <0.001; L14A, 0.178. **d**, Design model of mEpoR (green) and CHAMP-1 (cyan) TM complex with residues subjected to mutation labeled. Top, mEpoR (sticks) and red Cα atoms (spheres); bottom, CHAMP-1 with Cα atoms (spheres) colored as in **a**. WT, wild-type. **P < 0.05.

Next, we used mutagenesis to identify amino acids in the mEpoR and CHAMP-1 TM domains required for this inhibition. We first measured the effect of mEpoR mutants containing single- and double-amino acid substitutions from hEpoR at the three dissimilar TM positions (Fig. 4a,b). Compared to the 58% ± 5% reduction of the final cell count after 6 days upon CHAMP-1 expression in cells expressing wild-type mEpoR (*n* = 10), CHAMP-1 showed similar potency in mEpoR-L235V-expressing cells (55% ± 15% reduction) and modestly dampened inhibition in mEpoR-S237L-expressing cells (43% ± 8%). L238V-expressing cells showed significantly reduced responsiveness to CHAMP-1 (20% ± 11% reduction in proliferation; *P* < 0.003). S237L/L238V-expressing cells were not inhibited (1% ± 13% reduction), while L235V/S237L-expressing cells and L235V/L238V-expressing cells were still partially inhibited by CHAMP-1 (27% ± 4% and 26% ± 8% reduction, respectively).

**Extended Data Fig. 6:**
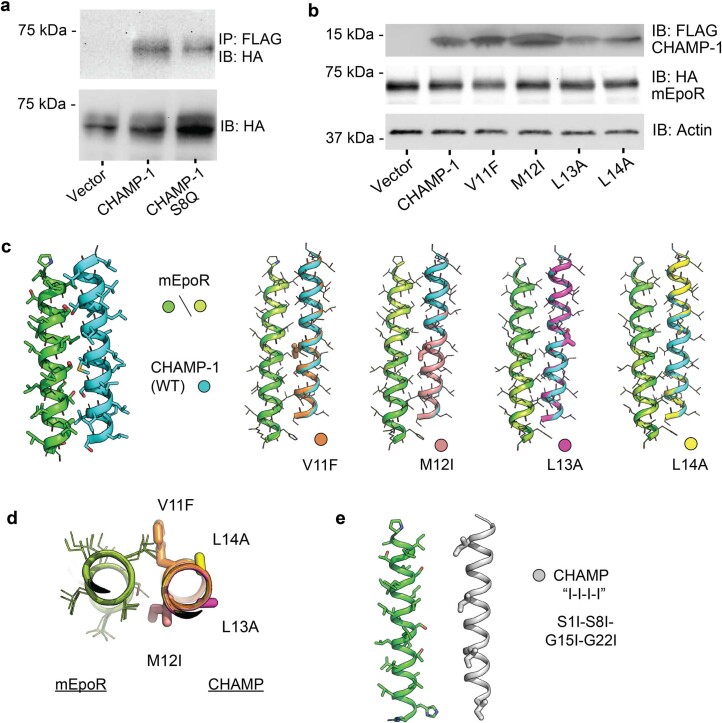
CHAMP-1 mutagenesis and ab initio prediction of CHAMP-1 mutant complexes. **(a)** Extracts from BaF3/mEpoR expressing empty vector or FLAG-tagged CHAMP-1 or mutant SQ8 were immunoprecipitated with anti-FLAG, subjected to SDS-PAGE, and probed with anti-HA to detect HA-tagged mEpoR. Bottom panel shows samples without prior immunoprecipitation. **(b)** Western blots of extracts of BaF3/mEpoR cells stably expressing wildtype or mutant CHAMP-1. Blots were probed with antibodies recognizing CHAMP (anti-FLAG), mEpoR (anti-HA), and actin as a loading control. Performed once (n = 1). **(c)** ESMfold predictions of CHAMP-1 and mutants with the mEpoR TM domain. Left, mEpoR (green) with CHAMP-1 wildtype (WT, cyan). Right, predictions for the EpoR/CHAMP-1 complex baring point mutations on CHAMP-1: V11F (orange), M12I (pink), L13A (magenta), L14A (yellow), overlain with the wildtype complex. **(d)** Top view of point mutant predictions of CHAMP-1 in complex with mEpoR, overlain and colored as in panel **c**. **(e)** ESMfold prediction of mEpoR (green) with CHAMP-1 ‘I-I-I-I’ mutant (gray, Ile in sticks) results in two distant non-interacting helices. Source data

Similarly, a panel of CHAMP-1 mutants was tested (Fig. 4a,c). Mutants S8Q, S8N, S8D and S8E lost all inhibitory potency; cells proliferated indistinguishably from cells transduced with empty vector. CHAMP-1-T19Q was modestly less inhibitory than CHAMP-1 (30% ± 8% reduction in cell count compared to parental BaF3/mEpoR cells lacking CHAMP-1, *P* = 0.01). The S1Q was completely tolerated, inducing inhibition similar to that with wild-type CHAMP-1 (58% ± 7%, *P* < 0.001). Interestingly, even though CHAMP-1-S8Q failed to inhibit proliferation, this mutant still co-immunoprecipitated mEpoR (Extended Data Fig. 6a). The S8Q, S8N, S8D and S8E mutants could lose their potency due to reduced interaction with EpoR or a lower monomeric pool of CHAMP-1, given that strongly polar membrane-embedded side chains often drive TM domain self-association in a depth-dependent manner^48^. CHAMP-1 and CHAMP-2 differ at positions 11 and 12, with sequences of VM and AA, respectively, highlighting additional tolerated amino acids. We also explored apolar disruptive mutations. CHAMP-1 small-X_6_-small residues S1-S8-G15-G22 were mutated to either isoleucine (I1-I8-I15-I22 (I-I-I-I)) or leucine (L1-L8-L15-L22 (L-L-L-L)). I-I-I-I CHAMP-1 exhibited significantly lower inhibitory potency, with a 34% ± 28% reduction in cell number versus the 58% reduction due to CHAMP-1 (*P* < 0.05), but interestingly the mutant did not completely abolish activity (Fig. 4c). By contrast, L-L-L-L lost inhibitory potency and instead induced EPO-independent proliferation similar to previously engineered polyleucine TM proteins (Extended Data Fig. 2a)^49^. We also tested point mutations at four consecutive positions, V11−L14, in an attempt to define the CHAMP-1 helix register binding mEpoR. V11F showed significantly impaired inhibitory activity (24% ± 3% reduction in proliferation, *P* < 0.05), whereas M12I and L14A exhibited only modestly reduced potency relative to CHAMP-1 and the differences did not reach statistical significance (54% ± 9% and 36% ± 16%, respectively). L13A, having the lowest expression level, showed no inhibition (1% ± 3%; Extended Data Fig. 6b). Thus, mutagenesis did not identify a helix register, as the most impactful substitutions, V11F and L13A, lie on opposite faces of CHAMP-1’s TM helix. Interestingly, ab initio predicted^40^ models of each point mutant (Extended Data Fig. 6c,d) adopted backbone conformations nearly identical to that in the native complex, revealing that the conformations for the mutated side chains may be tolerated within the interface. By contrast, the I-I-I-I mutant is predicted not to interact with mEpoR. Many factors, such as mutant expression level (Extended Data Fig. 6e), membrane trafficking or reduced monomeric availability may be responsible for an apparent reduction in potency, but these are factors that we did not rigorously quantify. While many substitutions are tolerated, changes at the small-X_6_-small motif, as well as other sites, mitigate CHAMP-1’s ability to inhibit mEpoR.

### Solution NMR characterizing CHAMP-1 interaction with mEpoR

**Fig. 5:**
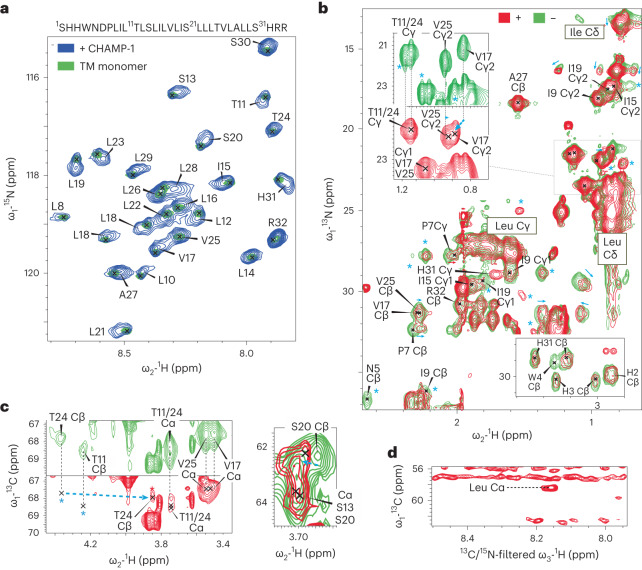
Solution NMR of the side chain-mediated CHAMP-1−mEpoR complex in DPC micelles. **a**, mEpoR-TM2 sequence and [^1^H-^15^N]-HSQC spectra in [^2^H]-DPC at 200 µM U-^15^N,^13^C,^1^H with 40 mM sodium acetate pH 5.2, 20 mM NaCl, 0.5 mM EDTA and 5 mM dithiothreitol (DTT) (45 °C, 800 MHz). Monomeric (green; peaks, X’s) and CHAMP-1-bound states (blue; 1 mol %) were independently assigned^58,59^. **b**, [^1^H-^13^C]-HSQC spectra of mEpoR-TM2 monomer (green) and CHAMP-1-bound (red) states from **a** have widespread differences: chemical shift perturbations (cyan arrows); new or broadened peaks (cyan asterisks). Top inset (5⨯ contour), V17 Cɣ2-Hɣ2 peak shift. **c**, Target epitope residues. Left, shift perturbation of T24 Cβ (cyan arrow), new unassigned peaks (cyan asterisks), and broadening of T11 Cα/Cβ and T24 Cα resonances. Right, shift perturbation of S20 Cβ−Hβ resonance, alongside broadening of S13 Cα, S13 Cβ and S20 Cα. **d**, Two-dimensional F1-[^13^C]-edited/F3-[^13^C,^15^N]‐filtered HSQC‐NOESY spectrum. Transferred NOE crosspeaks indicate direct contact between mEpoR-TM2 ^13^C atoms, for example, leucine Cα and CHAMP-1 ^14^N/^12^C-attached protons, for example, backbone amide proton(s).

**Extended Data Fig. 7:**
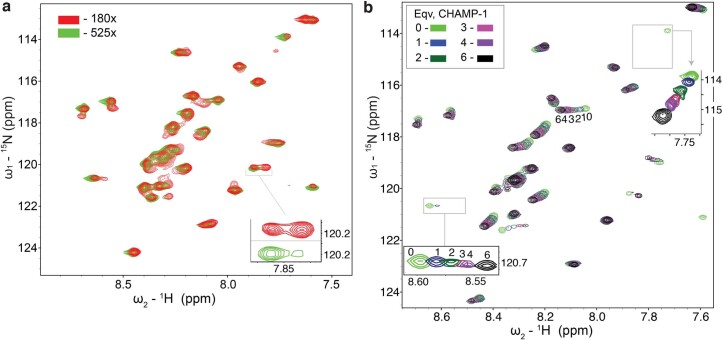
^15^N mEpoR-TM2 monomer-homodimer behavior in C14-Betaine micelles and binding to CHAMP-1. **(a)** The ^1^H-^15^N HSQC spectra of 0.3 mM ^15^N mEpoR-TM2 at 45 °C, pH 5.2, and 800 MHz when diluted in myristyl-sulfobetaine (C14B) micelles at 180 and 520 molar equivalents of detergent (54 mM, red; 156 mM, green). The more concentrated sample (180x, red) shows a strong second set of peaks emerging in exchange that is slow on the NMR time scale, likely representing the mEpoR-TM2 in the homodimeric state. *Right inset*, example pair of related, interconverting peaks dependent on the detergent concentration. **(b)** Titration of 1, 2, 3, 4, and 6 molar equivalents (eqv.) of CHAMP (navy, dark green, red, purple, black, respectively) to 0.3 mM ^15^N mEpoR-TM2 where additional C14-B is added alongside each eqv. CHAMP to maintain a constant molar ratio of C14-B to total peptide of 180:1. Reference spectra (no CHAMP, green) in (**b**) is predominantly the monomeric species at 520:1 ratio C14-B:mEpoR-TM2. Insets track shifting resonances at lower contour.

**Extended Data Fig. 8:**
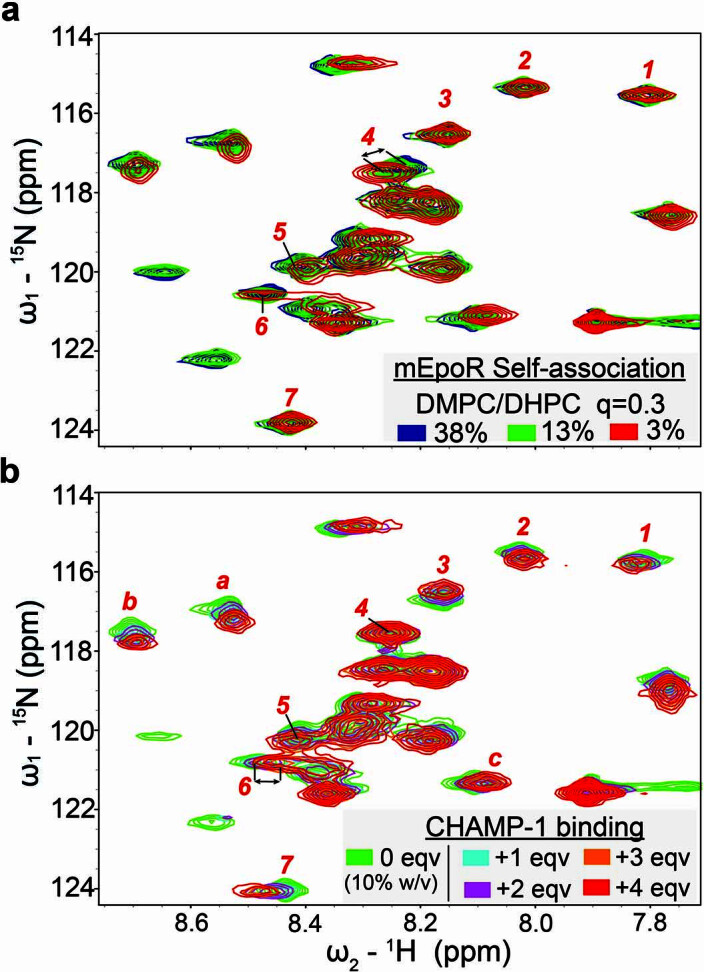
mEpoR-TM2 monomer-homodimer equilibrium and binding to CHAMP-1 in DHPC/DMPC q = 0.3 bicelles. (**a**) ^1^H-^15^N HQSC spectral changes of 0.5 mM ^15^N-mEpoR-TM2 at 45 °C, pH 5.2, and 800 MHz upon titration of DMPC/DMPC q = 0.3 bicelles at 38%, 13%, and 3% (w/v) at constant mEpoR-TM2 (navy, green, red, respectively). (**b**) ^1^H-^15^N HQSC spectral changes titration of of 0.5 mM ^15^N-mEpoR-TM2 at 45 °C, pH 5.2, and 800 MHz upon titration of 1, 2, 3, and 4 molar equivalent (eqv.) unlabeled CHAMP (cyan, orange, maroon, red, respectively) at a constant bicelle concentration of 10%. Spectra are overlaid on the CHAMP-free reference spectra in 10% bicelles. Both the mEpoR homo-oligomer (likely homodimer, panel a) and mEpoR-CHAMP heterodimer complex (panel b) exhibit chemical shift perturbation relative to monomer resonances with ‘fast’ chemical exchange. Numbered resonances denote key induced chemical shift changes that differ significantly between the two different types of titrations, differentiating that mEpoR-TM2 experiences distinct chemical environment in the mEpoR homodimer state versus in complex with CHAMP. Peaks 1, 2, 3, 6 and 7 do not shift as mEpoR converts from monomer to homodimer, but shift significantly upon CHAMP binding. Peak 4 shifts upon homodimerization but not upon CHAMP binding. Peak 5 shifts in different directions in panel **a** versus panel **b**. Lowercase letters denote resonances experiencing peak shift similar between panel a and panel **b**.

**Extended Data Fig. 9:**
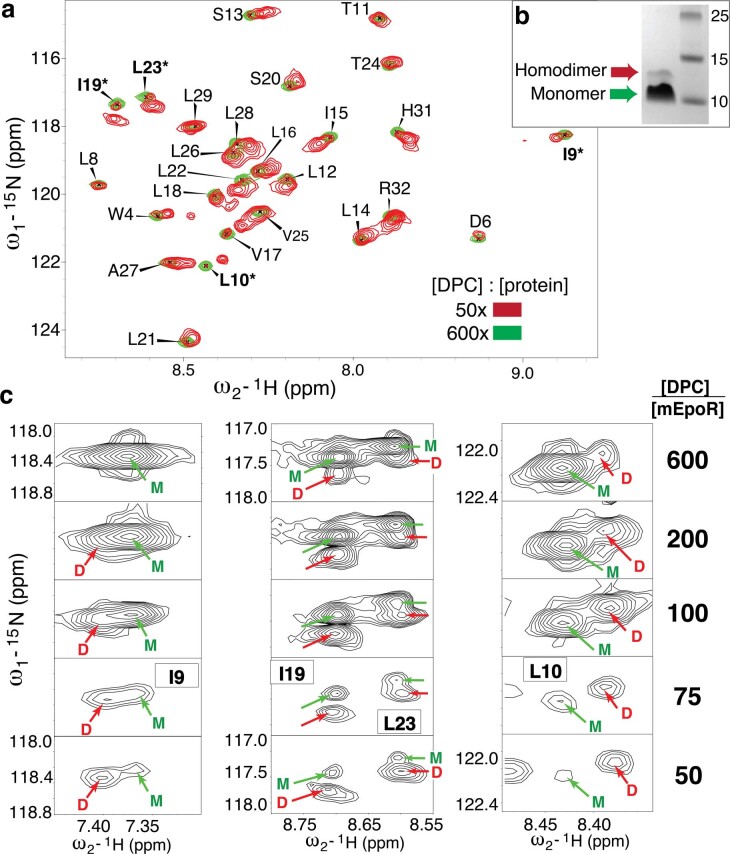
mEpoR Monomer-Homodimer equilibrium in DPC. **(a)** Assigned ^1^H-^15^N HSQC spectrum of 0.1 mM ^15^N mEpoR at high DPC molar ratio (600x, green) with the spectrum at low DPC concentration (50x, red) overlaid; the latter [DPC] concentration corresponds to approximately 1 TM peptide per 1 micelle. Recorded at 45 °C, 800 MHz, pH 5.2, with the predominantly monomer spectra (green) contoured 10-fold higher than the largely homodimer spectra (red). **(b)** SDS-PAGE of mEpoR showing non-covalent monomer-dimer equilibrium, representative gel of n = 3 replicate lanes. Mon, monomer. **(c)** Examples of newly emerging peaks (starred, bolded residues in panel **a**) of the mEpoR TM homodimer species upon reduction of DPC detergent concentration, titrated at 600, 200, 100, 75, and 50 molar ratios of detergent to TM peptide, showing the mEpoR TM peptide monomer-homodimer equilibria in the absence of CHAMP. Further concentrating mEpoR-TM2 to only 25x DPC molar ratio results in a sparse spectrum suggestive on peptide aggregation, where only 2 peaks (R32 and R33) are observed. M, monomer (green arrow); D, dimer, (red arrow). Source data

**Extended Data Fig. 10:**
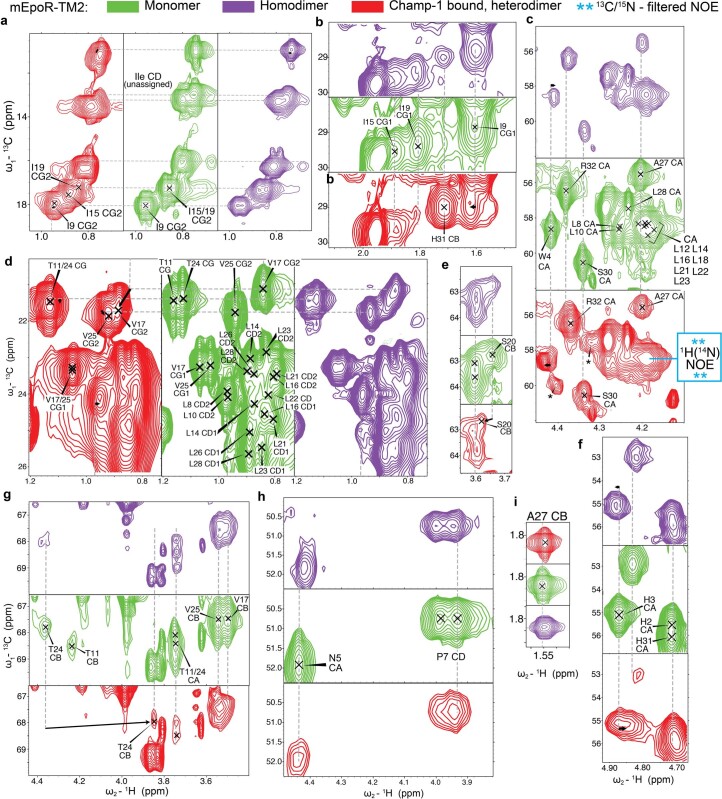
Comparison of ^1^H-^13^C HSQC spectra of mEpoR-TM2 from monomeric (green), CHAMP-1-bound (red), and partially homodimeric (purple) samples. **(a-i)** Different spectral regions perturbed. More monomeric resonances assigned from backbone-backbone and backbone sidechain experiments. Noted heterodimer assignments were derived independently by backbone-backbone, backbone-sidechain, and ^15^N-edited NOESY. *Cyan inset*, location of the isolated ^13^C resonance having a transfer NOE to a ^14^N-attached amide ^1^H peaks in the 13C-edited/^13^C,^15^N-filtered HSQC-NOESY (Fig. 5).

We next measured solution nuclear magnetic resonance (NMR) spectra for isotope-labeled mEpoR TM peptides in the presence of unlabeled CHAMP-1 peptides, and this was repeated for several constructs and membrane mimics (Supplementary Tables 2 and 3). To clearly differentiate chemical perturbations due to CHAMP-1 binding in each situation, we also systematically titrated detergent or bicelle concentration with mEpoR TM alone in parallel to identify spectral changes inherent to its monomer−homodimer equilibrium. First, we recorded [^1^H-^15^N]-HSQC spectra of U-^15^N-labeled mEpoR-TM1 reconstituted with and without 1.3 molar equivalents of CHAMP-1 in C14B micelle conditions mimicking our FRET experiment (180:1 detergent to peptide ratio) (800 MHz, 45 °C, pH 5.2) (Supplementary Fig. 2). New peaks emerged distinct from mEpoR’s homodimer resonances, indicating a slow-exchanging CHAMP-1-bound mEpoR population. A second mEpoR construct (mEpoR-TM2) and a different CHAMP-1 peptide having polar TM-flanking sequences were similarly assayed (Supplementary Table 2). CHAMP-1 titration to [U-^15^N]mEpoR-TM2 in C14B induced fast-exchanging chemical shift perturbations, while mEpoR-TM2’s monomer−homodimer equilibrium in C14B was in slow exchange (Extended Data Fig. 7). In 1,2-dimyristoyl-*sn*-glycero-3-phosphocholine (DPMC)/1,2-dihexanoyl-*sn*-glycero-3-phosphocholine (DHPC) *q* = 0.3 bicelles, fast-exchanging chemical shift perturbations were observed for both bicelle and CHAMP-1 titrations, yet they differed in directionality (Extended Data Fig. 8), allowing assignment of distinct monomeric, homodimeric and heterodimeric shifts. In DPC micelles, mEpoR-TM2’s well-dispersed monomeric ^1^H-^15^N resonances underwent distinct slow-exchanging behavior upon titration of CHAMP-1 (Fig. 5a–c) and lowered DPC concentration, allowing unambiguous classification of homodimer and heterodimer states (Extended Data Figs. 9 and 10). Thus, mEpoR and CHAMP-1 assembled for all model membranes and peptide construct combinations tested, albeit varying in exchange behavior. The CHAMP-1−mEpoR-TM2 complex could be isolated as the major species with spectra suitable for resonance assignment, with DPC giving the best spectral properties with an excess of both detergent (>400:1 DPC to mEpoR) and CHAMP-1 (>1.5 mol %, or 6−8 equivalents).

In [^2^H]-DPC, we characterized how CHAMP-1 binding alters mEpoR-TM2’s side chain environment. We first assigned [U-^15^N, ^13^C]-labeled mEpoR-TM2’s monomeric state in excess [^2^H]-DPC using triple-resonance HNCA and HNCB spectra followed by (H)CC(CO)NH and H(CC)(CO)NH backbone-side chain TOCSY spectra to separate heavily overlapping ^1^H-^13^C spectral regions (Supplementary Figs 3–5, and Supplementary Tables 4 and 5). For resonances of the mEpoR-TM2−CHAMP-1 complex, we independently repeated backbone and partial side chain assignments (Supplementary Fig. 5 and Supplementary Tables 4 and 5). Comparison of monomeric versus CHAMP-1-bound mEpoR-TM2 [^1^H-^13^C]-HSQC spectra showed induced broadening of select peaks, new resonances and widespread chemical shift perturbations across diverse side chain chemical groups (Fig. 5b,c and Extended Data Fig. 10). Numerous mEpoR side chains in close interaction (<4 Å) with CHAMP-1 in our design model experienced substantial changes, including V17 Cɣ2 (shift; Fig. 5b, inset, and Extended Data Fig. 10d), I19 Cɣ1 (broadening; Extended Data Fig. 10b), I19 Cɣ2 (shift; Extended Data Fig. 10a), T24 Cβ (shift; Fig. 5c, left, and Extended Data Fig. 10g), S13 and S20 overlapping Cα atom (broadening; Fig. 5c, right, and Extended Data Fig. 10e) and S20 Cβ (shift; Fig. 5c, right, and Extended Data Fig. 10e). Induced ^1^H-^13^C chemical shift perturbations upon CHAMP-1 binding were similar to those of homodimeric mEpoR in about 50% of resonances, including numerous assigned (for example, V17, A27, S30, H31 and R32) and unassigned peaks (Extended Data Fig. 10b−d,i). Likewise, CHAMP-1 induced many shift and intensity perturbations distinct from mEpoR homodimerization, including at mEpoR-TM2 residues contacting CHAMP-1 in the design model (I9, I19, S20 and T24; Extended Data Fig. 10b,e,g) and membrane-proximal residues (H3, W4, N5 and P7; Extended Data Fig. 10f,h).

**Fig. 6:**
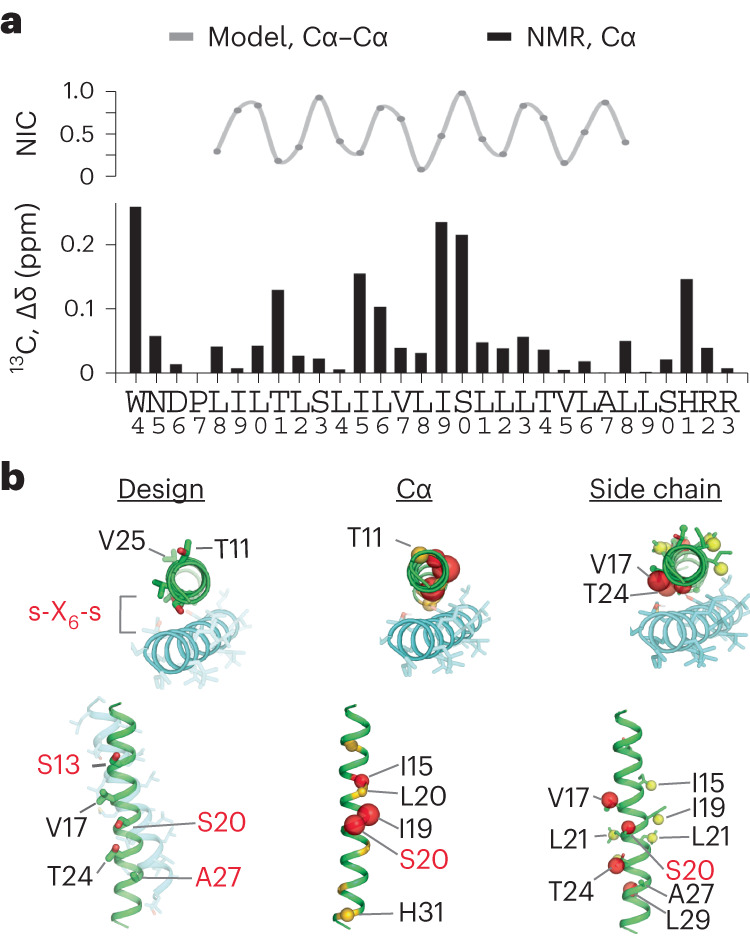
Mapping the CHAMP-1−mEpoR TM interface from chemical shift perturbations. **a**, Helical periodicity in mEpoR-TM2 Cα shift perturbation upon CHAMP-1 binding and its agreement with expected CHAMP-1−mEpoR Cα−Cα distances from the design model, plotted as normalized interhelical closeness (NIC; Methods), a metric tracking the minimum interhelical Cα−Cα distance for each residue in the model of the complex. **b**, Left, CHAMP-1 (cyan) and mEpoR (green) design model noting protein-facing and lipid-facing side chains (sticks) and targeted small-X_6_-small repeats (red). Middle, perturbed Cα atoms (scaled spheres, green−red color scale) lie on one face of mEpoR’s TM α-helix, overlapping the targeted epitope and CHAMP-1 interface; minimally perturbed Cα atoms are lipid-facing in the design model. Right, perturbed side chain atoms enriched at one helix face, including V17, S20 and T24.

Interestingly, per-residue shift perturbations (Δ*δ*) to mEpoR-TM2’s ^13^Cα atoms exhibited a clear pattern of three to four residue periodicity (Fig. 6a) mirrored to a lesser extent by ^1^H-^15^N Δ*δ* (Supplementary Fig. 7), possibly indicating its helical register and interaction surface with CHAMP-1. Notably, these Δ*δ* perturbations were roughly in phase with the interhelical Cα−Cα distances between mEpoR and CHAMP-1 within the core of our design model (Fig. 6a). mEpoR-TM2 Cα atoms with the largest Δ*δ* were closest to CHAMP-1, while the least perturbed residues were lipid-facing. However, this correlation was not exact and became out of phase toward both helix termini, suggesting a slightly different CHAMP-1 packing angle. A thorough comparison of side chain resonances along the mEpoR-TM2 TM helix was obfuscated by spectral overlaps, particularly for leucine residues. All the unambiguously assigned resonances with substantial spectral changes are plotted on mEpoR’s structure in Fig. 6b. While CHAMP-1 binding had a dispersed impact, the majority of highly perturbed side chain atoms lie at the helix face expected to bind CHAMP-1 (for example, V17, I19, S20 and T24).

Finally, we measured direct interatomic interaction between [^1^H,^13^C,^15^N]-mEpoR-TM2 and unlabeled CHAMP-1 in [^2^H]-DPC through an F1-[^13^C]-edited/F3-[^13^C,^15^N]‐filtered HSQC‐NOESY experiment. Intermolecular transfer resonances were observed between ^13^C-attached protons (mEpoR) and ^12^C- or ^14^N-attached protons (CHAMP-1) in the two-dimensional projection spectrum (Fig. 5d). One crosspeak could be attributed to a CHAMP-1 amide proton (8.14 ppm) interacting with an mEpoR-TM2 leucine ^13^Cα (58.4 ppm). Overlap of many leucine Cα peaks prevented unambiguous residue-specific assignment (Extended Data Fig. 10c). Nonetheless, these results indicate that the complex between CHAMP-1 and mEpoR-TM2 in DPC is direct and features tight backbone−backbone packing as in the small-X_6_-small-mediated design.

## Discussion

Here, we develop and successfully demonstrate a more automated and distinct implementation of the CHAMP algorithm wherein membrane protein-specific bioinformatics data guide the design of de novo TM domains to bind a target protein with conformational specificity. The mini-membrane proteins exhibit exquisite molecular recognition in cellular lipid bilayers, discriminating between the highly similar hEpoR and mEpoR. To assess whether our procedure encoded the intended custom binding mode with the mEpoR TM domain, we undertook rigorous biophysical characterization. Overall, the CHAMP-1−mEpoR complex in vitro and in vivo is consistent with the intended nonnative 1:1 heterodimeric antiparallel TM topology designed in silico. The TM complex forms readily and is stable in vitro across diverse membrane mimics, elevated temperatures and pH, —attributed to an extensive interaction network. Notably, CHAMP-1’s robust side chain-mediated binding contrasts with that of past library-selected synthetic EpoR-activating TM domains, which co-immunoprecipitated EpoR but induced negligible side chain chemical shift perturbations in analogous TM fragment solution NMR experiments^50,51^.

The precise structure and side chains stabilizing the complex are not fully clear and consistent between our cellular and biophysical data. NMR spectra in DPC micelles show that residues I236 and S237 of mEpoR are most strongly perturbed (that is, I19 and S20 of the mEpoR-TM2 peptide) along with V234 (V17) and T241 (T24). These residues constitute a continuous helix face shared with the mEpoR TM surface (S230-S237-A244) that we targeted in our design, implying their interaction with CHAMP-1 (Fig. 6b). Yet, inhibition by CHAMP-1 in BaF3 cells was most sensitive to mEpoR substitutions at L238 and S237, but not I236, suggesting that the former residues contact CHAMP-1. Notably, both sets of experiments are consistent with S237 participating in the complex. However, the TM helix register implied by interface mapping using activity measurements in living cells differs between solution NMR perturbations in DPC. Residues L238 and I236 lie on opposite faces of mEpoR’s TM domain (~200° helix rotation apart) and thus cannot both simultaneously contact monomeric CHAMP-1. It is possible that different conformations of TM helix packing and ensembles of interacting amino acids exist in each distinct chemical environment. There is precedent for similar behavior, with integrin β3 having alternative TM interfaces inferred from experiments performed in human versus bacterial cell membranes^52^.

On CHAMP-1, substitutions at small-X_6_-small positions reduced or reversed CHAMP-1’s influence on BaF3/mEpoR cell proliferation, indicating that small-X_6_-small residues are important for interaction. V11F, in phase with the small-X_6_-small motif, slightly reduced inhibition. However, the L13A substitution on the opposite helix face fully abrogated inhibition. Changes at adjacent sites (L14A, M12I and V11A-M12A within CHAMP-2) had minimal effect. Interestingly, the negative-control design assembled into antiparallel complexes with mEpoR-TM in vitro, suggesting that the small-X_6_-small motif is sufficient to encode interaction. However, this TM protein control did not co-immunoprecipitate with mEpoR or inhibit signaling in BaF3/mEpoR cells, indicating that this sequence is missing features critical for in vivo interaction and activity in the cellular environment. Conversely, S8Q was not inhibitory, but still immunoprecipitated mEpoR. Thus, the exact structure−activity relationship of our designed TM domains remains difficult to parse from inhibition in vivo. This is not entirely surprising, as many important membrane protein properties that affect anti-EpoR activity in cells can be altered upon TM protein mutation, including expression level, cell surface trafficking, additional competitive TM interactions (self-assembly or off-target), etc. Nonetheless, the intended mEpoR signaling inhibition mechanism of CHAMP-1, TM binding in an antiparallel orientation, was successfully encoded.

We demonstrated the design of small expressible TM proteins completely from scratch to control interaction topology and effectively perturb a signal-amplifying surface receptor. The CHAMP-1 sequence may serve as a novel tool complementing existing engineered water-soluble and TM polypeptides for studying the EPO receptor’s signal conduction mechanism^53–56^ and its role in erythropoiesis and other activities^57^. The technological advances described here should facilitate accessibility and increasing complexity in design of tool molecules targeting diverse membrane proteins directly at their bioactive TM regions.

## Methods

### Computational design

A 25-residue ideal α-helix was used to build an initial model for the monomeric mEpoR TM domain using RosettaMP’s FastRelax protocol implemented in RosettaScripts^38^ to calculate the lowest energy orientation in an implicit bilayer. All simulations with RosettaMP used the score function weights: mpframework_smooth_fa_2012 (ref. ^32^). The second interacting CHAMP helix was built from mining curing a database of interacting pairs of TM domain helices present in natural membrane protein X-ray structures^32^, specifically, ‘Cluster 6’ of close-packing antiparallel TM helices with a left-handed crossing from this work. This cluster contained 141 natural TM helix−helix examples of unique sequence. These pairs of TM sequences were subsequently filtered into those having three consecutive small-X_6_-small repeats and that were at least 22 residues long, for both helices. Six TM helix pairs matched these criteria. For these six structures, we calculated the best-fit coiled-coil parameters of their backbone coordinates to Crick’s coiled-coil equations for the special case of antiparallel symmetry implemented using the coiled-coil Crick parameterization (CCCP) octave source code described in ref. ^34^. Parameters representative of the helix−helix geometries (listed in the main text) were used to generate de novo coordinates for the corresponding idealized dimeric antiparallel coiled coil using the same code from ref. ^34^. One helix was superposed onto the mEpoR TM domain model in the register of the small-X_6_-small motif, thus creating a knowledge-based template positioning the second polyalanine CHAMP helix.

Sequence design rotamer trials were performed using RosettaMP, implementing Rosetta LayerDesign, by packing the side chain rotamer trials first at core positions, then at interface boundary positions and finally at noninterface positions. Then, a FastRelax step followed, including minimization, rotamer repacking (fixed side chain identity) and rigid body re-orientation of the TM domain complex in the implicit bilayer. The designation of each CHAMP residue (interface or lipid-facing) and the sequence logo outputs are shown in Extended Data Fig. 1b. Small-X_6_-small motif interface positions were limited to alanine, serine or glycine. Interface positions at alternate helix turns were limited to alanine, serine, threonine, valine, isoleucine, methionine, leucine and phenylalanine. Lipid-facing residues were allowed to be selected from this alphabet during the Rosetta modeling, but identities were later reassigned using an automated script selecting apolar amino acids at random from a weighted probability distribution as previously described^17^: alanine, isoleucine, valine and phenylalanine at 10% probability and leucine at 60% probability, with no additional Ala-X_6_-Ala or Ala-X_3_-Ala motifs being allowed to form at lipid-facing positions. Each model was evaluated for the absence of large packing voids at the helix−helix interface by RosettaHoles^39^, that is, the PackStat filter and its total Rosetta energy, taking the top 10% of PackStat scores for subsequent selection. These top sequences were clustered hierarchically using the BLOSOM matrix corresponding to the average pairwise sequence identity of the sequences (BLOSOM85).

Three clusters of CHAMP designs resulted, and the top-ranked sequence by PackStat score was automatically suggested for experimental testing by the analysis script. One CHAMP cluster differed from the other two by having serine rather than glycine at the final small-X_6_-small position, which induced nonideal helix geometry in the mEpoR TM domain. Thus, we chose not to test this design or cluster, making this the only application of human intervention from visualizing molecular models. The top sequences from the remaining two clusters constituted CHAMP-1 and CHAMP-2, which differ only at two non-small-X_6_-small mid-spanning positions (Fig. 1). Both CHAMP-1 and CHAMP-2 minimized molecular models had a pair of interhelical hydrogen bond networks engaging mEpoR’s serine and threonine side chains (Extended Data Fig. 1c). All steps were performed using scripts that automated the decision-making process using the above-described rules for model building, design, ranking and sequence selection.

The no-design TM domain sequence was derived from a ‘rules’-based selection based on interface residues directly extracted from two TM domains interacting via small-X_6_-small motifs within a natural membrane protein, detailed in Extended Data Fig. 1d–g. From the set of structurally clustered antiparallel TM helix−helix interactions used for CHAMP design, six curated natural examples, we modeled and searched whether threading mEpoR’s sequence on each of the 12 TM domains resulted in a clash with the adjacent TM helix using Rosetta fixed-backbone rotamer repacking (‘fixbb’). Only 1 of the 12 possible mEpoR-threaded model cases had no steric clashes with the adjacent natural TM domain: TM domains 1 and 2 of the photosystem II light-harvesting complex (PSII, PDB:3BZ1) chain B. mEpoR was threaded onto TM2 but showed essentially no homology to this PSII TM span (<22% sequence identity; Extended Data Fig. 1e). The sequence of the no-design control TM protein was derived from the interface residues of the PSII chain B TM1, replacing PSII lipid-facing residues with semi-randomly selected apolar amino acids as described above (Extended Data Fig. 1f,g). No sequence changes were modeled to optimize the interface with mEpoR. Two residues, alanine and leucine, were added to the C terminus to extend the TM domain to match CHAMP sequence designs in the number of small-X_6_-small residues at four, while ensuring that alanine was not the last apolar residue. The final no-design control sequence and the natural source TM1 from PS II had 48% sequence identity.

Constructs for protein expression were designed such that the N terminus and Flag tag could be in the cytoplasm and a neutral C terminus was far enough past the TM domain that the charged carboxylic acid was in the lumen. Synthetic peptides of CHAMP-1 were designed specific to each experiment, varying the composition of polar residues flanking the TM domain and usually including a tryptophan for spectroscopic detection.

The ESMfold^40^ server was used to predict ab initio the lowest energy structures of mEpoR and CHAMP complexes, connecting TM domain sequences with a ten-residue polyglycine flexible linker. ESMfold was preferred to OmegaFold because the latter predicted noninteracting helices, and ESMfold was preferred to AlphaFold2 because the former has superior performance in the absence of multiple-sequence alignment data. Predictions performed with a 20-glycine linker gave the same structures. Thus, parallel helix orientation is a possible outcome but was not predicted.

### Peptide synthesis and purification

TM peptides were synthesized by solid-state fmoc microwave synthesis with ChemMatrix rink amide resin (Biotage) using a Biotage Initiatior+ Alstra, cleaved using a trifluoroacetic acid (TFA) cocktail (Sigma) from solid-phase resin, and then purified by RP-HPLC as previously described^17^. All peptides were produced as C-terminal carboxamides with free amino N termini, except for biotin−nCys−mEpoR. Precursor nCys−mEpoR peptide was labeled at its free amino N terminus as a protected peptide on resin with NHS-biotin (Sigma) by swelling the resin with dimethylformamide (DMF), adding 10 equivalents of *N,N*-di-isopropylethylamine, and then adding 1.5 molar equivalents of NHS-biotin dissolved in minimal DMF and stirring at room temperature for 45 min, performed twice. Peptides were purified by RP-HPLC using a C4 prep column (10-μm, 214TP, Vydac) using a linear gradient of solvent A (water, 0.1% TFA) and solvent B (60/30/9.9/0.1 isopropanol/acetonitrile/water/TFA). Peptide purity of >95% was achieved in all cases and confirmed using analytical HPLC (C4, Vydac). Correct product masses were confirmed by MALDI mass spectrometry using the matrix α-cyano-4-hydroxycinnamic acid (Sigma).

Peptides for fluorescence quenching, cCys−CHAMP-1 and nCys–mEpoR, were labelled in solution with fluorescein-5-maleimide and (diethylamino-4-methylcoumarin-3-yl)maleimide, respectively (Anatrace). Then, 4 mg of lyophilized peptide was dissolved with 10 molar equivalents of maleimide-derivatized fluorophore in 1 ml of DMF, and 0.2 ml of water with pH 7 HEPES buffer (final, 25 mM), followed by incubating the reaction overnight at room temperature under nitrogen gas on a rotating shaker. The fluorescently labeled peptide products were purified by HPLC as described above.

### FRET-based fluorescence quenching of TM peptides

Following published protocols^45^, donor (diethylamino-4-methylcoumarin-3-yl)maleimide)-labeled mEpoR TM peptide was reconstituted at different donor-to-CHAMP-1 acceptor molar ratios at a fixed total peptide concentration across the titration, fixed peptide to detergent ratio, and total ratio of mEpoR to CHAMP-1 at equimolar. mEpoR from a trifluoroethanol (TFE, Sigma) stock solution was mixed with a separate ethanol stock solution of unlabeled CHAMP-1 and fluorescein-5-maleimide-labeled CHAMP-1 to yield final concentrations of 1.5 μM labeled donor mEpoR and 1.5 μM total CHAMP-1 (combined unlabeled and acceptor-labeled) alongside different amounts of C14B (0.5 mM (~100:1 detergent to peptide), 0.725 mM (~175:1) or 0.95 mM (~250:1)). For C14B, CMC = 0.2 mM and has 90 detergent monomers per micelle. The organic solvent peptide−detergent mixtures were evaporated under vacuum and then reconstituted in 50 mM Tris-HCl pH 8, 100 mM NaCl, 0.5 mM EDTA and 5 mM Tris(2-carboxyethyl)phosphine (TCEP) and subjected to bath sonication, vortexed, equilibrated overnight in the dark and then aliquoted in triplicate into 96-well black round-bottom plates and read in a SpectraMax H5 via monochromator (Molecular Devices). Fluorescence emission scans were recorded upon excitation at 410 nm (435-nm cut-off). Donor relative ration fluorescence intensity (460 nm) was monitored for samples reconstituted with increasing molar ratios of labeled CHAMP-1 acceptor (with compensating unlabeled CHAMP-1 removed).

Theoretical equations for FRET-based donor emission decay across donor/acceptor titration for oligomeric complexes of different stoichiometry (monomer, dimer, trimer, etc.) were plotted using classic theoretical equations^46,47^. A crowding factor was calculated based on C14B concentration in the micelle phase and estimated peptide per micelle ratio using Poisson statistics, accounting for additional donor quenching from nonspecific micelle co-occupation or collision.

### Thiol-disulfide equilibrium exchange

The two peptide mixture samples were prepared by mixing a TFE stock solution of nCys−mEpoR, an ethanol stock solution of cCys−designed TM peptide and a methanol stock solution of either DPC or POPC to a final total peptide to detergent ratio of 1:100 and equimolar peptide ratio. Solutions were dried under nitrogen gas and vacuum overnight and then reconstituted at a 100 μM concentration of each peptide in 100 mM Tris-HCl pH 8.6, 100 mM KCl and 1 mM EDTA with 0.45 mM oxidized glutathione (GSSG) and 1.05 mM reduced glutathione (GSH) to initiate reversible redox conditions. After overnight equilibration, samples were quenched with HCl (0.1 M final concentration). Reaction mixtures were separated by analytical RP-HPLC using a C4 column (Vydac 214TP, 5 μm) and quantified by integration of ultraviolet (UV) chromatogram peaks to quantify the relative species fractions of disulfide-bonded dimer species: homodimers and heterodimers. The identity of each species was confirmed by mass spectrometry.

The biotin capture thiol-disulfide exchange procedure was performed to isolate only the mEpoR-containing species from the nine possible monomeric or disulfide-bonded species when three cysteine-containing peptides (nCys−mEpoR, cCys−CHAMP-1, nCys−CHAMP-1) were mixed for competitive reversible oxidation. Peptides were co-dissolved with detergent in TFE, dried under a gaseous nitrogen stream, lyophilized and reconstituted in an aqueous solution. Biotinylated nCys−mEpoR was reconstituted at 100 µM with fourfold molar excess of CHAMP, 200 µM nCys−CHAMP-1 and 200 µM cCys−CHAMP-1, in 20 mM C14B (40:1 detergent to total peptide ratio) in 20 mM Tris pH 8 and 50 mM NaCl, as well as 3 mM glutathione (5:1, [GSH]/[GSSG]), and then allowed to undergo reversible oxidation overnight. TM peptide oxidation was confirmed by SDS−PAGE (Extended Data Fig. 3c). The mixture was quenched by the addition of concentrated sodium acetate (lowering the pH to 4.5) and bound in batch to streptavidin-conjugated biotin beads overnight, capturing a fraction of the biotin−nCys−mEpoR monomeric and disulfide-bonded species. Noncovalently associated TM peptides were washed from the beads with excess detergent to increase the detergent/protein ratio and the dilution in the micelle phase, facilitating dissociation from bound mEpoR-TM peptide: 5 bead volumes, 5 times, 200 mM C14B, 100 mM sodium acetate, 50 mM NaCl. Peptides that were disulfide bonded to mEpoR were eluted from the beads by washing with 10 mM TCEP added to the same high-C14B-content buffer, reducing disulfide bonds and also diluting noncovalent TM peptide interactions. The eluted material was separated by analytical RP-HPLC using a linear solvent gradient on a C4 column, integrating the UV chromatogram to quantify relative species mole fractions as described above.

### Expression of isotope-enriched mEpoR TM domain fragments

The sequence encoding the mEpoR TM domain fused either to His-tagged T4 lysozyme (cysteine-free mutant) with a thrombin cleavage site or to His-tagged SUMO with a sequence-specific nickel-assisted cleavage (SNAC) site^60^ was cloned into a pET28 vector. Proteins were expressed in BL21(DE3) in M9 minimal medium supplemented with 0.5 g [^15^N]NH_4_Cl_2_ (Cambridge Isotopes) and 0.2 g of isotope-enriched ISOGROW (Sigma) per liter of culture, with or without 3 g of [^13^C]d-glucose replacing natural d-glucose (Sigma). Cells were induced with 0.4 mM IPTG at an optical density of 0.8 followed by overnight growth at 30 °C or 37 °C. Pelleted cells were suspended in a lysis buffer for solubilizing inclusion bodies: 8 M urea, 0.5 mM EDTA, 50 mM sodium phosphate pH 7.5, 2% (w/v) SDS. Cycles of tip sonication (10 min) and rotary shaking (30 min) were repeated until a clear homogeneous (nonviscous) solution was achieved. After centrifugation (30 min, 35,000*g*), the lysate was poured over a gravity column of Ni-NTA agarose resin (HisPur, Thermo Fisher). Resin was washed with 10 column volumes of detergent-free lysis buffer and then washed with 4 column volumes of 25 mM imidazole detergent-free lysis buffer before elution in lysis buffer containing 1% SDS and 250 mM imidazole. For thrombin cleavage, the eluted protein was repeatedly concentrated, the buffer was exchanged to remove excess SDS into 50 mM Tris pH 8, 100 mM NaCl with 0.1% *n*-Dodecyl-β-d-maltoside using a 30-kDa centrifugal filter (EMD Millipore) and the sample subjected to overnight dialysis using a 20-kDa membrane (Slide-a-lyzer, Thermo) for trace SDS removal. For SNAC peptide tag self-cleavage, imidazole was removed (<0.5 mM) from the protein sample using a 10-kDa centrifugal filter and the sample was buffer exchanged into 100 mM *N*-cyclohexyl-2-aminoethanesulfonic acid (CHES) pH 8.5, 100 mM NaCl, with NiCl_2_ added to reach a 2 mM final concentration. Due to the high residual SDS content, SNAC cleavage was performed at 42 °C for >95% completion in 24−36 h. The cleaved isotope-enriched mEpoR TM domain peptide was then purified by RP-HPLC and lyophilized (~10 mg of peptide per 1-liter culture).

### Solution NMR in membrane mimics

Isotopically enriched mEpoR-TM fragments in TFE stock solution of known concentration were combined with synthetic CHAMP-1 peptides (ethanol) along with lipid or detergent, dried under a nitrogen gas stream and further dried under vacuum. Samples were reconstituted in NMR buffer (40 mM sodium acetate pH 5.2, 20 mM NaCl, 0.5 mM EDTA, 10 mM DTT, 5% (v/v) D_2_O) and then sonicated, filtered (0.2-μm) and transferred to a 3-mm Shigemi tube. Spectra of labeled mEpoR fragments with and without CHAMP were recorded at 45 °C on a Bruker 800-MHz spectrometer with cryogenic triple-resonance probes: [^1^H-^15^N]- and [^1^H-^13^C]-HSQC, HNCA, HNCB, (H)CC(CO)NH, H(CC)(CO)NH, [^13^C]-edited NOESY-HSQC and [^13^C]-edited/[^13^C,^15^N]-filtered HSQC-NOSEY according to Bruker’s standard pulse sequences. In addition, HNCA and HN(CO)CA spectra were recorded for a sample comprising 800 mM [^2^H]-DPC, 2 mM [^1^H,^15^N,^13^C]mEpoR-TM2 and 12 mM unlabeled CHAMP-1 on a Bruker 900-MHz spectrometer with a triple-resonance cryogenic probe. Spectra were processed in NMRPipe^59^. Assignment and analysis were performed using Sparky^58^.

Chemical shift perturbation at Cα atoms was normalized (0 to 1) to the largest induced shift value and plotted on the monomeric TM helix of mEpoR (Fig. 6), thus scaling the relative sphere size and color (green, least perturbed; red, most perturbed) of each Cα atom. For side chain atoms, ^1^H or ^13^C atoms with measured shift perturbation were split into three groups according to the magnitude of the perturbation (green, least perturbed; yellow, modestly perturbed; red, most perturbed), not including resonances broadened beyond detection (S13, T11).

### Cloning and vectors for mammalian cell expression

The HA-tagged hEpoR and HA-tagged mEpoR genes were originally obtained from S. Constantinescu (Ludwig Institute) and subcloned into pMSCV-neo (Clontech) using EcoRI and HpaI restriction sites. The chimeric mhm-EpoR and mEpoR mutants containing point mutations in the mEpoR TM were constructed using double-stranded DNA gBlock gene fragments (Integrated DNA Technologies), as previously described^50^. The construct encoding the human PDGF-βR TM domain flanked by a signal sequence was described previously^41^. The hEpoR−GFP1-10 fusion protein was constructed by replacing the C-terminus of hEpoR downstream of residue 258 with a 10-amino acid flexible linker (GGSGGGGSGG) followed by the sequence encoding the GFP1-10 fragment (residues 1−215) using DNA gBlock gene fragments and BglII restriction sites. The mEpoR−GFP1-10 fusion protein was constructed by replacing the sequence encoding hEpoR_1−258_ in hEpoR−GFP1-10 with the sequence encoding mEpoR_1−257_ using DNA gBlock gene fragments and HpaI and BstBI restriction sites. Similarly, GFP1-10 was fused after the TM domain of ErbB2. All noninducible GFP11 fusion proteins were constructed by cloning DNA gBlock gene fragments into pMSCV-neo by using EcoRI and XhoI restriction sites. The Dox-responsive ErbB2-TM−GFP11 was constructed by cloning DNA gBlock gene fragments into pTight-puro by using BamHI and EcoRI restriction sites. The general structure of the ErbB2-TM−GFP-11 and glycophorin A TM−GFP11 proteins takes the form hEpoR signal peptide−TM domain–GFP11.

### Cells, retroviral infections and growth inhibition assays

Human embryonic kidney (HEK) 293 T cells were maintained in DMEM-10: DMEM supplemented with 10% FBS (Gemini Bioproducts), 4 mM l-glutamine, 20 mM HEPES (pH 7.3) and 1⨯ penicillin/streptomycin (P-S). To produce retrovirus stocks, 2 μg pantropic pVSV-G (Clontech), 3 μg pCL- (Imgenex) and 5 μg of the retroviral expression plasmid of interest were mixed with 250 μl of 2⨯ HEBS. Then, 250 μl of 0.25 M calcium chloride was added to each mixture while bubbling. The mixture (~500 μl) was incubated for 20 min at room temperature and then added dropwise to 2.0 × 10^6^ 293 T cells plated the day before in 100-mm tissue culture dishes in DMEM-10. The cells were incubated with the transfection mixture for 6−8 h at 37 °C and the medium was replaced with 5 ml fresh DMEM-10. The cells were incubated for another 48 h at 37 °C and then the viral supernatant was harvested, filtered through a 0.45-μm filter (Millipore) and either used immediately or stored at −80 °C.

Mouse IL-3-dependent BaF3 and derivative cells were maintained in RPMI-10 medium: RPMI-1640 supplemented with 10% heat-inactivated FBS, 5% WEHI-3B cell-conditioned medium (as the source of IL-3), 4 mM l-glutamine, 0.06 mM β-mercaptoethanol and 1⨯ P-S. BaF3 cells expressing mEpoR, hEpoR and all EpoR mutants and chimeras were generated by infecting BaF3 cells with pMSCV-neo vector containing the desired HA-tagged EpoR gene. BaF3 cells (5 ⨯ 10^5^) were washed with PBS and then resuspended in 500 μl of RPMI-10 medium with 4 μg ml^−1^ polybrene. Then, either 500 μl of retroviral supernatant or 500 μl of DMEM-10 for mock infection was added to the re-suspended cells and then incubated for 8 h at 37 °C. After incubation, 9 ml of RPMI-10 was added and the cells were incubated overnight at 37 °C before selection in 1 mg ml^−1^ G418. Wild-type and mutant CHAMP proteins cloned in MSCV-puro were introduced into cells by infection, followed by selection in 1 μg ml^−1^ puromycin.

For proliferation assays, 2 ⨯ 10^5^ BaF3 and derivative cells expressing the appropriate genes were washed in PBS three times to remove IL-3. Cell pellets were resuspended in 10 ml RPMI-10 lacking WEHI-3B cell-conditioned medium but supplemented with 0.06 U ml^−1^ human EPO (Epoetin Alfa, Amgen). Viable cells were counted 6−8 days after IL-3 removal. All growth inhibition assays were performed in at least three independent biological replicates (that is, independent infections to express TM proteins). All reported experiments included positive and negative controls that performed as expected, and no outliers in these experiments were excluded. All graphs show average cell counts ± s.e.m. The statistical significance of differences between control and experimental samples was evaluated by either one-tailed or two-tailed Student’s *t*-tests with unequal variance, performed using the T.TEST function in Microsoft Excel (2013).

### Construction and analysis of inducible cell lines

BaF3 cells were transduced to express an engineered version of the tetracycline-controlled transactivator protein, tTA-Advance (tTA), via retroviral infection with the pRetroX-Tet-Off Advanced (Clontech) vector and selection with 1 mg ml^−1^ G418. CHAMP-1 or ErbB2-TM cloned in the expression vector pRetroX-TIGHT-puro (Clontech) was introduced into cells expressing tTA by retroviral infection and selection with 1 μg ml^−1^ puromycin. HA−mEpoR was retrovirally transduced with pMSCVneo (Clontech) and selected with 0.6 U ml^−1^ human EPO (Epoetin Alfa, Amgen) in the absence of IL-3.

To assess expression levels of the CHAMP proteins, BaF3/mEpoR/tTA cells expressing a CHAMP protein were grown in 10-ml cultures in RPMI-10/IL-3 medium in the absence of Dox or supplemented with 100 or 200 pg ml^−1^ Dox for 48 h. Cells were pelleted in the presence of 1 mM phenylmethylsulfonyl fluoride (PMSF) for 10 min at 1,500 r.p.m. at 4 °C. Cell extracts were prepared and 20−30 μg of total protein was electrophoresed. After transfer to 0.2-µm polyvinylidene fluoride (PVDF) membranes and blocking in 5% milk in TBST (20 mM Tris, 150 mM NaCl, 0.1% Tween 20), the blots were incubated overnight at 4 °C with 1:1,000 anti-Flag-HRP (Sigma-Aldrich) in 5% milk in TBST. Blots were then washed and visualized using enhanced chemiluminescence.

For proliferation assays, BaF3/mEpoR/tTA cells expressing CHAMP proteins were first cultured in 10 ml of RPMI-10/IL-3 medium in the absence of Dox or supplemented with 100 or 200 pg ml^−1^ Dox for 48 h. Then, 2 ⨯ 10^5^ BaF3 cells were washed in PBS, resuspended in IL-3-free medium supplemented with the same concentration of Dox and 0.06 U ml^−1^ human EPO, and counted as described above.

### Immunoprecipitation and immunoblotting

To assess protein phosphorylation, BaF3 cells and their derivatives were first starved in RPMI-10 IL-3-free medium for 3 h at 37 °C and were then acutely stimulated with 1 U ml^−1^ EPO for 10 min at 37 °C. Cells were then washed twice with ice-cold PBS containing 1 mM PMSF. For phosphotyrosine and phospho−protein blots, 1⨯ HALT protease and phosphatase inhibitor cocktail (Thermo Scientific) and 500 μM hydrogen peroxide-activated sodium metavanadate were also added to the wash solution. Cells were lysed in Flag-lysis buffer (50 mM Tris pH 7.4, 150 mM NaCl, 1 mM EDTA, 1% Triton X-100) supplemented with protease and phosphatase inhibitors as described above. All lysates were incubated on ice for 20 min, followed by centrifugation at 14,000 r.p.m. for 30 min at 4 °C. The total protein concentration of the supernatants was determined using a bicinchoninic acid (BCA) protein assay kit (Pierce).

To immunoprecipitate Flag-tagged CHAMP peptides, 50 μl of anti-FLAG M2 matrix gel (Sigma-Aldrich) was added to 0.5 mg of total protein and rotated overnight at 4 °C. Immunoprecipitated samples were washed four times with 1 ml NET-N buffer (100 mM NaCl, 0.1 mM EDTA, 20 mM Tris-HCl pH 8.0, 0.1% Nonidet P-40) supplemented with protease inhibitors as above, pelleted and resuspended in 2⨯ Laemmli sample buffer (2⨯ SB) supplemented with 200 mM DDT and 5% β-mercaptoethanol (β-ME). Precipitated proteins and whole cell lysates were heated at 95 °C for 5 min and then resolved by SDS−PAGE on 7.5%, 10% or 20% polyacrylamide gels according to the size of the protein of interest. The resolving gel was then transferred by electrophoresis to a 0.2-μm nitrocellulose or PVDF membrane and 0.09% SDS was added to the transfer buffer for membranes used to detect phosphorylated proteins.

Membranes were blocked with gentle agitation for 2 h at room temperature in 5% nonfat dry milk in TBST. To detect the phosphorylated forms of JAK2 and STAT5, anti-phospho-JAK2 (Tyr1008) (clone D4A8, Cell Signaling) and anti-phospho-STAT5 (Y694) (9351, Cell Signaling) were used. To detect the total JAK2 and STAT5, anti-JAK2 (clone D2E12, Cell Signaling) and anti-STAT5 (9363, Cell Signaling) were used. A horseradish peroxidase (HRP)-conjugated mouse anti-HA antibody (clone 6E2, Cell Signaling) was used to detect HA-tagged EpoR and all EpoR mutants. All antibodies were used at a 1:1,000 dilution. Membranes were incubated overnight with gentle agitation in primary antibody at 4 °C, washed five times in TBST and then incubated with gentle agitation for 1 h at room temperature in a 1:10,000 dilution of donkey anti-mouse or donkey anti-rabbit HRP (Jackson Immunoresearch), as appropriate. To re-probe membranes, they were stripped in Restore Western Stripping Buffer (Thermo Scientific) for 15 min at room temperature with gentle agitation, washed five times in TBST, blocked in 5% milk in TBST for 1 h at room temperature and incubated overnight at 4 °C with antibody, as described above. Membranes were incubated with Super Signal West Pico or Femto chemiluminescent substrate (Pierce) to detect protein bands.

### Split GFP complementation assay

BaF3 cells were transduced to express the GFP1-10 fragment fused to EpoR, the GFP11 fragment fused to CHAMP-1, or both. The EpoR−GFP1-10 fusion protein consists of (from the N terminus to the C terminus) residues 1−258 from hEpoR or residues 1−257 from mEpoR, a 10-amino acid flexible linker (GGSGGGGSGG) and the GFP1-10 segment. The GFP11−N1 fusion proteins consist of (from the N terminus to the C terminus) a Flag tag, the GFP11 segment (residues 216−231), a GGG linker and the CHAMP-1 sequence. For flow cytometry, 5 ⨯ 10^5^ cells were collected by centrifugation at 1,000 r.p.m. for 10 min at 4 °C and then washed in cold PBS and resuspended in 300 µl cold PBS. Cells were then analyzed on a CytoFLEX equipped with a green laser and the data were plotted in FlowJo.

### Reporting summary

Further information on research design is available in the Nature Portfolio Reporting Summary linked to this article.

## Online content

Any methods, additional references, Nature Portfolio reporting summaries, source data, extended data, supplementary information, acknowledgements, peer review information; details of author contributions and competing interests; and statements of data and code availability are available at 10.1038/s41589-024-01562-z.

### Supplementary information

Supplementary Information Supplementary Figs. 1−7 and Tables 1−5.

Reporting Summary

### Source data

Source Data Fig. 1 Unprocessed western blots.

Source Data Fig. 3 Unprocessed western blots.

Source Data Extended Data Fig. 2 Unprocessed western blots.

Source Data Extended Data Fig. 3 Unprocessed gel.

Source Data Extended Data Fig. 4 Unprocessed western blots.

Source Data Extended Data Fig. 6 Unprocessed western blots.

Source Data Extended Data Fig. 9 Unprocessed gel.

## Data Availability

Chemical shift data have been uploaded to BMRB, with entry assigned accession number 51401. Source data are provided with this paper.

## Code availability

Custom scripts for molecular model building, Rosetta design configuration files, example command lines, intermediate files, and analysis scripts for ranking Rosetta output files are publicly available and hosted on a GitHub repository, including Rosetta. The uploaded software is compatible with the latest version of Rosetta: https://github.com/mmravic314/CHAMP2023/.
