# Supplementary material for: De novo-designed transmembrane proteins bind and regulate a cytokine receptor
Source: Nat Chem Biol. 2024 Mar 13;20(6):751–60. doi: 10.1038/s41589-024-01562-z (PMC11142920; doi:10.1038/s41589-024-01562-z)
Supplement: Supplementary file 1 — Supplementary Figs. 1−7 and Tables 1−5. [file 41589_2024_1562_MOESM1_ESM.pdf]

nature chemical biology

Article

https://doi.org/10.1038/s41589-024-01562-z

De novo-designed transmembrane proteins bind and regulate a cytokine receptor

In the format provided by the authors and unedited

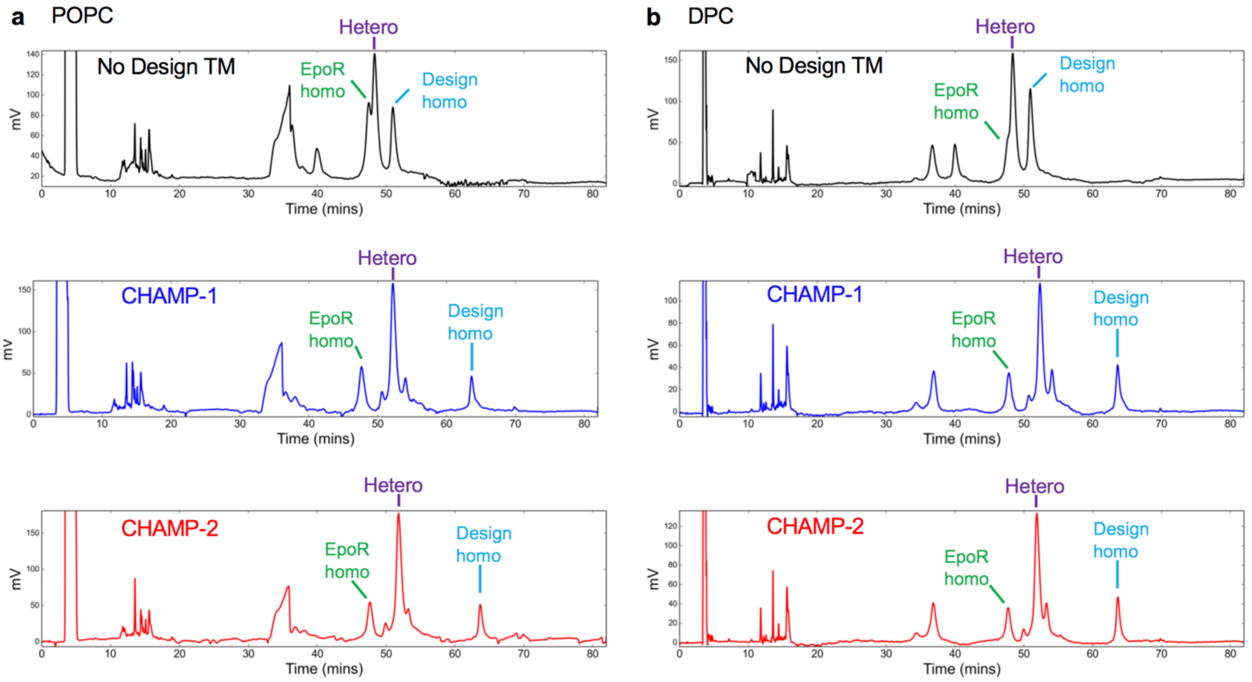

Supplementary Figure 1. Raw RP-HPLC traces of mEpoR and designed TM peptide thiol-disulfide exchange experiments

(a) 220 nm UV chromatogram (representative of 3 replicate runs; signal reported as detector millivolts, mV) of equimolar mixtures of nCys mEpoR mixed with the No Design (top, black), CHAMP-1 (middle, blue), or CHAMP-2 (bottom, red) peptide reconstituted in POPC lipid vesicles in degassed mixed glutathione buffer after equilibrium oxidation (peptide primary sequences in Supplementary Table 2). Peaks corresponding to each disulfide bonded dimer species are annotated, colored as in Figure 2a: EpoR homo., homodimer (green); Hetero, design-mEpoR heterodimer (purple); design homo, homodimer (cyan). These traces are the raw data which were subject to peak integration, and decovolution in cases of overlap, to yield fraction of disulfide bonded species reported in Figure 2a

(b) 220 nm UV chromatogram (representative of 3 replicate runs) of equimolar mixtures of mEpoR mixed with the No Design (top, black), CHAMP-1 (middle, blue), or CHAMP-2 (bottom, red) peptide reconstituted in DPC micelles after equilibrium oxidation. Peaks labelled as in panel **a**. These traces are the raw data analyzed and reported in Extended Data Fig. 3a.

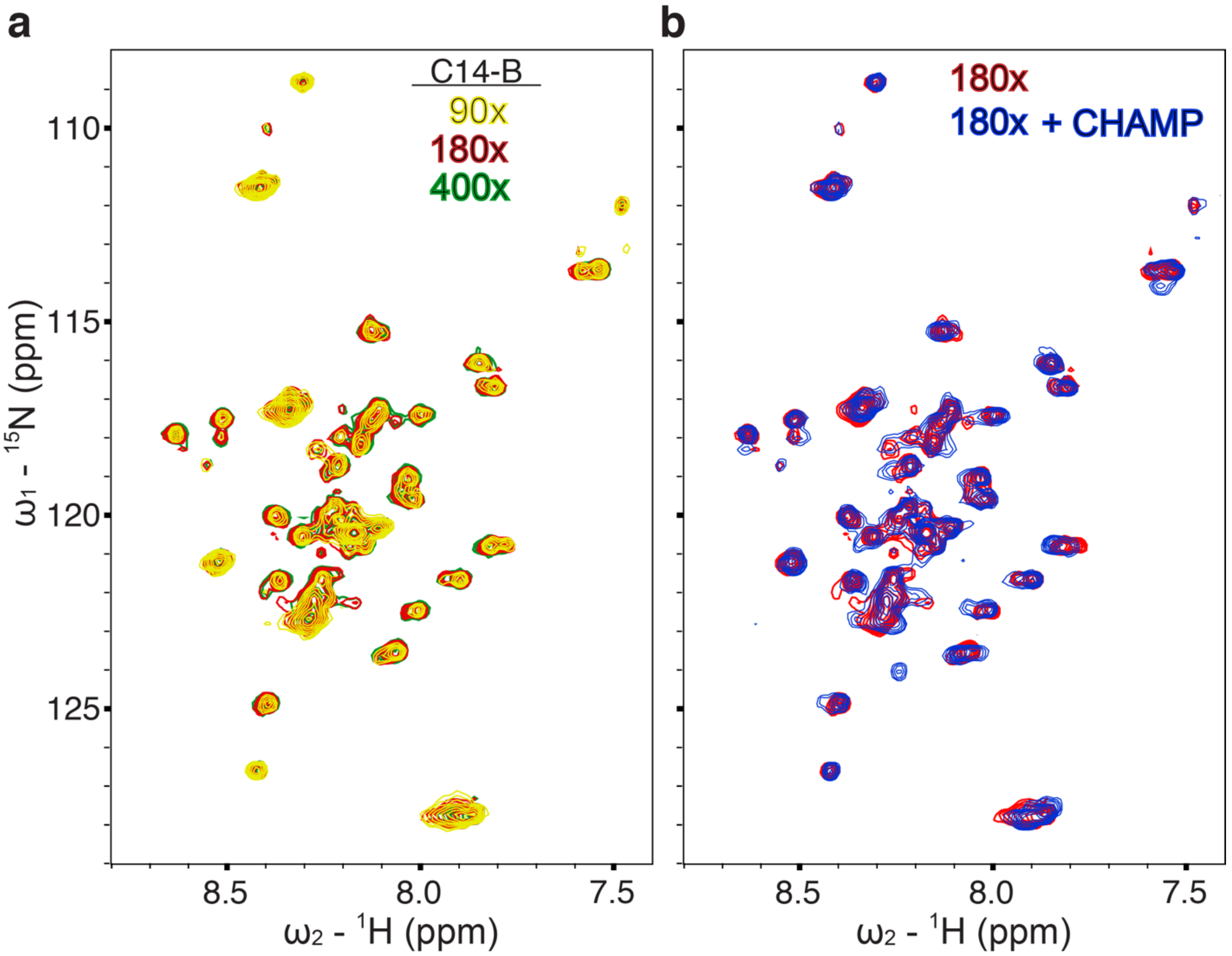

Supplementary Figure 2. ^{15}N mEpoR-TM1 monomer-homodimer behavior in C14-Betaine micelles and binding to CHAMP-1

(a) ^{1}H-^{15}N HSQC spectra of 0.3 mM ^{15}N mEpoR-TM1 when titrated with myristyl-sulfobetaine (C14-B) micelles at 90, 180, and 400 molar equivalents of detergent to peptide (27 mM, yellow; 54 mM, red; 120 mM, blue) in 40 mM sodium acetate buffer pH 5.2, 20 mM NaCl, 0.5 mM EDTA, 5 mM DTT at 45° C and 800 MHz. C14-B has approximately 90 detergent molecules per micelle, i.e. aggregation number. Multiple populations are observed which show resonance-dependent peak broadening over the titration, but negligible chemical shift perturbations.

(b) The ^{1}H-^{15}N HSQC spectra is shown overlaid for ^{15}N mEpoR-TM1 at 0.3 mM with (blue) and without (red) addition of unlabeled CHAMP-1 at 1.35 molar equivalents. The total detergent:protein molar ratio of 180 was kept constant, i.e. 0.4 mM CHAMP, 108 mM C14-B; 360x C14-B per mole mEpoR-TM1). Many new peaks are observed and some existing peaks show large decays in intensity, indicative of mEpoR-TM1's interaction with CHAMP-1 slow on the NMR timescale.

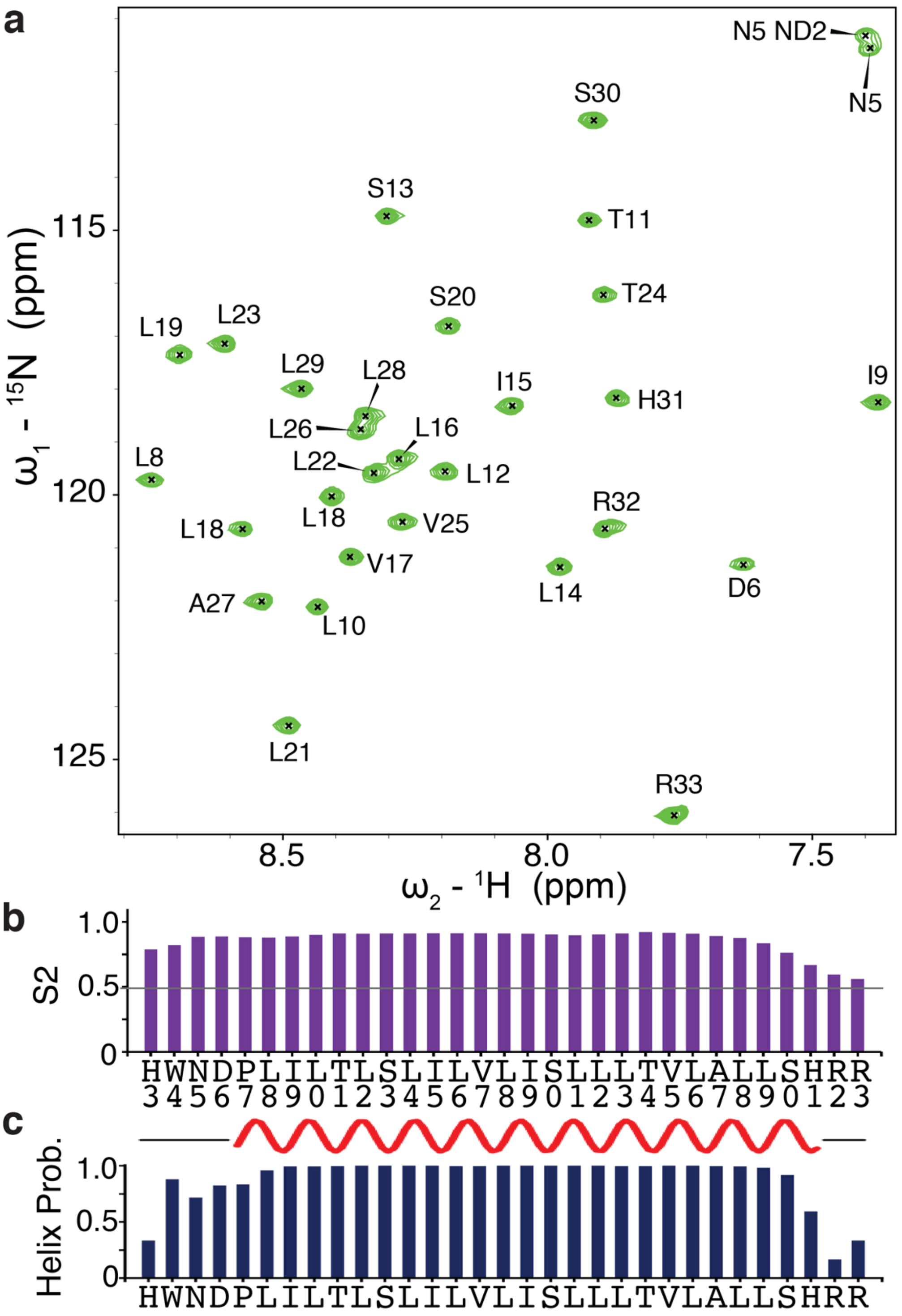

Supplementary Figure 3. **^{1}H-^{15}N HSQC spectra and per-residue structural features of monomeric mEpoR-TM2 in excess DPC.**
(a) Assigned ^{1}H-^{15}N HSQC spectra of 0.2 mM ^{15}N mEpoR at 45° C, 800 MHz, pH 5.2, and high [DPC] : [protein] molar ratio of 600 (roughly 12 micelles per TM peptide monomer). **(b)** Predicted order parameter (S2) from TALOS+. **(c)** Predicted helicity from chemical shift index software. *Top*, predicted helical (red) and disordered (black) residues from CSI-3. *Bottom*, probability of helical secondary structure predicted by TALOS+. Prob., probability.

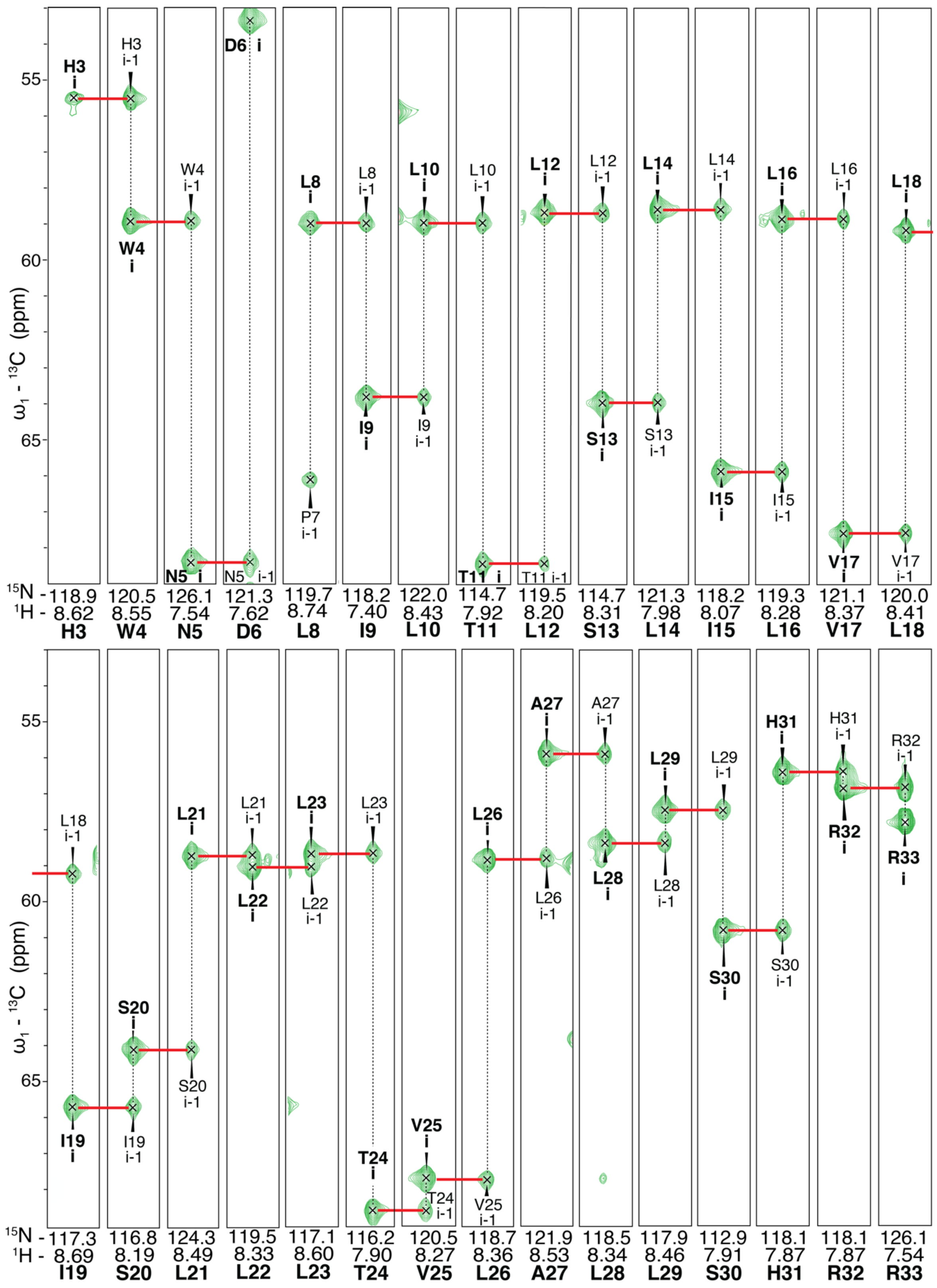

Supplementary Figure 4. HNCA Assignment of mEpoR-TM2 monomer. $^{1}H^{13}C^{15}N$ mEpoR at 1 mM in 400 mM ^{2}H-DPC at 45° C, 800 MHz, pH 5.2. Red lines denote *i* and *i-1* resonance connectivity; X axis, ^{15}N. Pro 7 assigned by L8 i-1 and lack of ^{15}N-^{1}H i peak or D6 i-1 peaks.

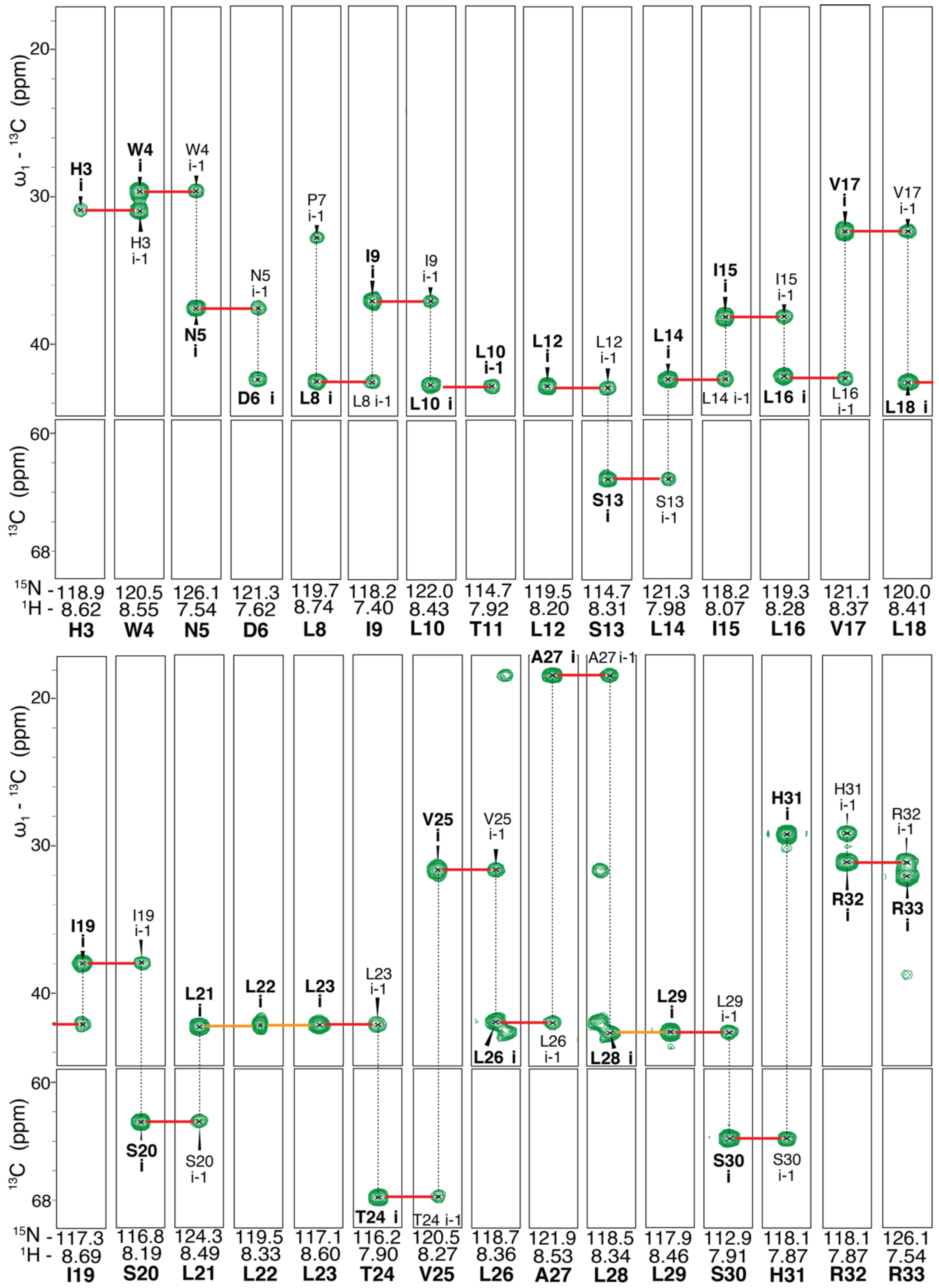

Supplementary Figure 5. HNCB Assignment of mEpoR-TM2 monomer. $^{1}H^{13}C^{15}N$ mEpoR at 1 mM in 400 mM ^{2}H-DPC at 45° C, 800 MHz, pH 5.2. Orange lines denote ambiguous connectivity due to overlap of *i* and *i-1* resonances. Red lines denote *i* to *i-1* links. X axis, ^{15}N. T11 displays no residue *i* CB-N resonance and L12 shows no *i-1* CB-N resonance to T11. Although a resonance exists in the T11 N-H strip, which is likely the L10 *i-1* CB-N resonance.

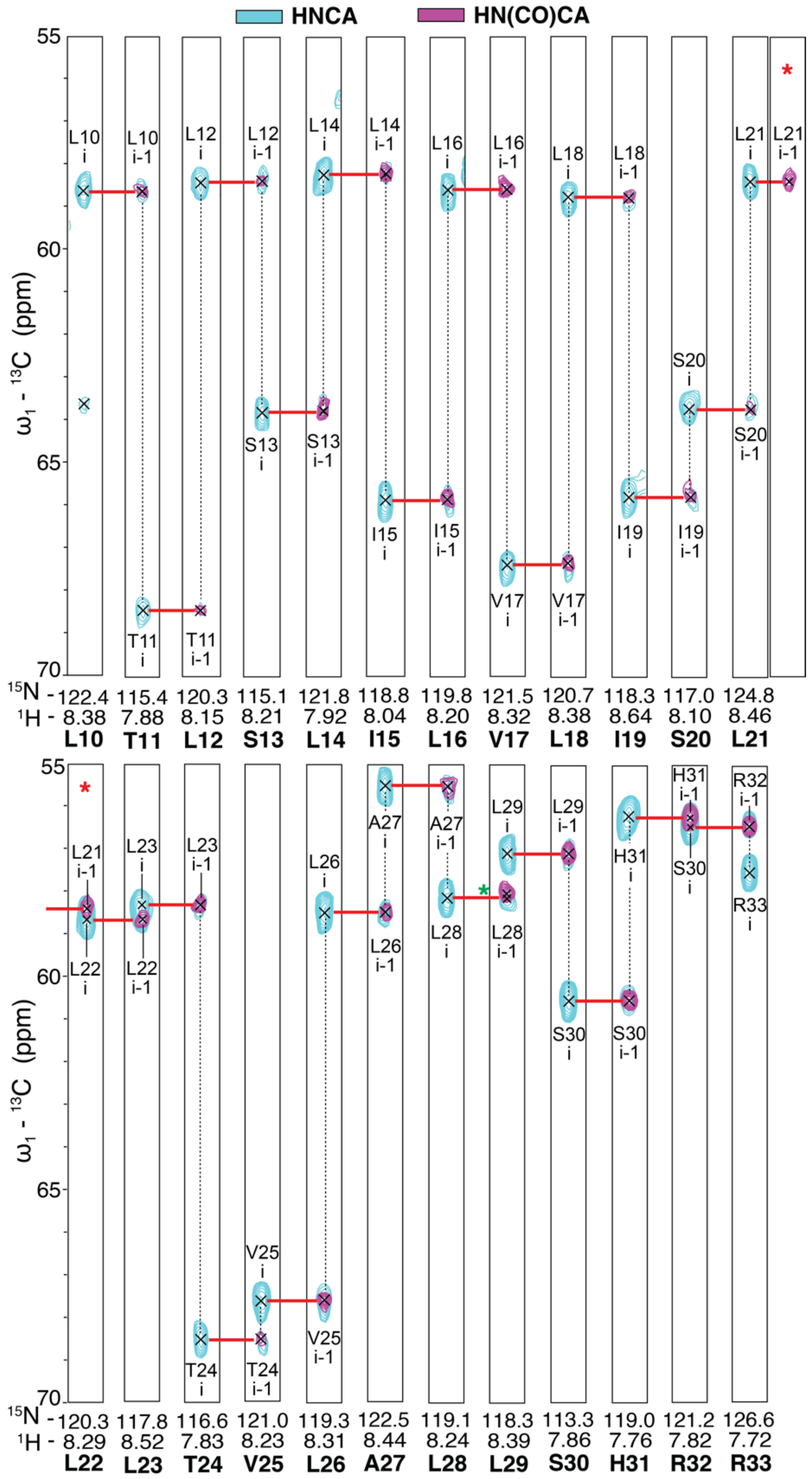

Supplementary Figure 6. Paired HNCA and HNcoCA spectra of $^{1}H^{13}C^{15}N$ mEpoR in with CHAMP-1. $^{1}H^{13}C^{15}N$ mEpoR at 2 mM with 10 mM CHAMP-1 in 800 mM ^{2}H-DPC at 45° C, 900 MHz, pH 5.2; HNCA, cyan; HNcoCA, magenta. Red lines denote *i* and *i-1* resonance connectivity; X axis, ^{15}N. Red asterisk, repeated L22 strip of HCcoCA to show connectivity to L21 across rows of strips. Green asterisk, discrepancy in HNCA and HNcoCA peak position of L28 CA by 0.15 ppm (^{13}C). H31, R32, and R33 resonances contoured 5, 10, 20 times higher, respectively.

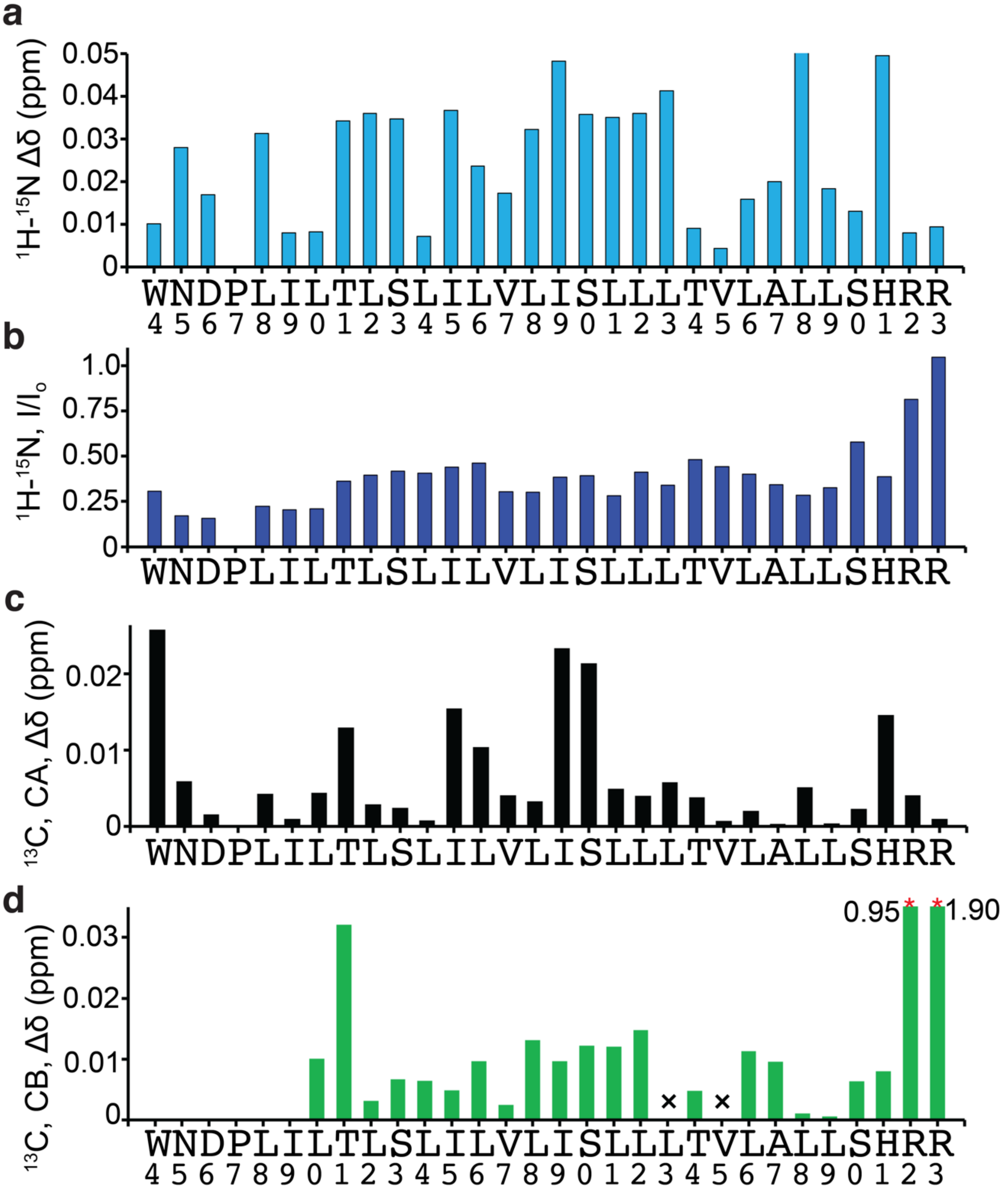

Supplementary Figure 7. Per-residue chemical shift and intensity perturbations of mEpoR CHAMP-1 bound state versus monomeric state.

(a) Hybrid ^{1}H-^{15}N chemical shift perturbation of monomeric and CHAMP-1 bound mEpoR-TM2.

(b) Relative intensity (I) between ^{1}H-^{15}N resonances in HSQC spectra of monomeric and CHAMP-1 bound mEpoR-TM2.

(c) CA chemical shift perturbation of monomeric and CHAMP-1 bound mEpoR-TM2.

(d) CB chemical shift perturbation of monomeric and CHAMP-1 bound mEpoR-TM2. Missing residues notes by 'x'. R32 and R33 are off-scale, and their induced shift values listed on top.

Supplementary Table 1. Expressed protein constructs in BaF3 cells
The designed CHAMP-1 TM domain underlined.

**mEpoR**
MDKLRVPLWPRVGPLCLLLAGAAWAPSPSLYPYDVPDYAPDPKFESKAALLASRGSEELLCFTQRLED<br>LVCFWEEAASSGMDFNYSFSYQLEGESRKSCSLHQAPTVRGSVRFWCSLPTADTSSFVPLELQVTEA<br>SGSPRYHRIIHINEVVLLDAPAGLLARRAEEGSHVVLRWLPPPGAPMTTHIRYEVDVSAGNRAGGTQR<br>VEVLEGRTECVLSNLRGGTRYTFAVRARMAEPSFSGFWSAWSEPASLLTASDLDPLILTLSLILVLISLL<br>LTVLALLSHRRTLQQKIWPGIPSPESEFEGLFTTHKGNFQLWLLQRDGCLWWSPGSSFPEDPPAHLEV<br>LSEPRWAVTQAGDPGADDEGPLLEPVGSEHAQDTYLVLDKWLLPRTPCSENLSGPGGSVDPVTMDE<br>ASETSSCPSDLASKPRPEGTSPSSFEYTILDPSSQLLCPRALPPELPPTPPHLKYLYLVVSDSGISTDYS<br>SGGSQGVHGDSSDGPYSHPYENSLVPDSEPLHPGYVACS
**mhmEpoR**
MDKLRVPLWPRVGPLCLLLAGAAWAPSPSLYPYDVPDYAPDPKFESKAALLASRGSEELLCFTQRLED<br>LVCFWEEAASSGMDFNYSFSYQLEGESRKSCSLHQAPTVRGSVRFWCSLPTADTSSFVPLELQVTEA<br>SGSPRYHRIIHINEVVLLDAPAGLLARRAEEGSHVVLRWLPPPGAPMTTHIRYEVDVSAGNRAGGTQR<br>VEVLEGRTECVLSNLRGGTRYTFAVRARMAEPSFSGFWSAWSEPASLLTASDLDPLILTLSLILVVILVL<br>LTVLALLSHRRTLQQKIWPGIPSPESEFEGLFTTHKGNFQLWLLQRDGCLWWSPGSSFPEDPPAHLEV<br>LSEPRWAVTQAGDPGADDEGPLLEPVGSEHAQDTYLVLDKWLLPRTPCSENLSGPGGSVDPVTMDE<br>ASETSSCPSDLASKPRPEGTSPSSFEYTILDPSSQLLCPRALPPELPPTPPHLKYLYLVVSDSGISTDYS<br>SGGSQGVHGDSSDGPYSHPYENSLVPDSEPLHPGYVACS
**mEpoR-TM**
MDHLGASLWPQVGSLCLLLAGAAWAPPLDPLILTLSLILVLISLLLTVLALLSHRRALKQKIWPGIPSPES<br>EGPYSNPYENSLIPAAEPLPPSYVACS
**ErbB2-TM-GFP11**
MDHLGASLWPQVGSLCLLLAGAAWPVTFIIATVVGVLLFLILVVVVGILGGSGGGGSGGRDHMVLHEY<br>VNAAGIT
**GpA-TM-GFP11**
MDHLGASLWPQVGSLCLLLAGAAWERVQLAHHFSEPEITLIIFGVMAGVIGTILLISYGIRRLIGGSGGG<br>GSGGRDHMVLHEYVNAAGIT
**ErbB2-TM-GFP1-10**
MDHLGASLWPQVGSLCLLLAGAAWPVTFIIATVVGVLLFLILVVVVGILGGSGGGGSGGMVSKGEELFT<br>GVVPILVELDGDVNGHKFSVRGEGEGDATIGKLTLKFICTTGKLPVPWPTLVTTLTYGVQCFSRYPDHM<br>KRHDFFKSAMPEGYVQERTISFKDDGKYKTRAVVKFEGDTLVNRIELKGTDFKEDGNILGHKLEYNFN<br>SHNVYITADKQKNGIKANFTVRHNVEDGSVQLADHYQQNTPIGDGPVLLPDNHYLSTQTVLSKDPNEK
**PDGFβR-TM**
MRLPGAMPALALKGELLLLSLLLLLEPQISQGLVVTPKVVVISAILALVVLTIISLIILIMLWQKKPRYEIRWK<br>VIESVSCPAPRAEAEDSFL

**mEpoR-GFP1-10**
MDKLRVPLWPRVGPLCLLLAGAAWAPSPSLYPYDVPDYAPDPKFESKAALLASRGSEELLCFTQRLED LVCFWEEAASSGMDFNYSFSYQLEGESRKSCSLHQAPTVRGSVRFWCSLPTADTSSFVPLELQVTEA SGSPRYHRIIHINEVVLLDAPAGLLARRAEEGSHVVLRWLPPPGAPMTTHIRYEVDVSAGNRAGGTQR VEVLEGRTECVLSNLRGGTRYTFAVRARMAEPSFSGFWSAWSEPASLLTASDLDPLILTLSLILVLISLL LTVLALLSHRRTLQQKIWGGSGGGGSGGMVSKGEELFTGVVPILVELDGDVNGHKFSVRGEGEGDAT IGKLTLKFICTTGKLPVPWPTLVTTLTYGVQCFSRYPDHMKRHDFFKSAMPEGYVQERTISFKDDGKYK TRAVVKFEGDTLVNRIELKGTDFKEDGNILGHKLEYNFNSHNVYITADKQKNGIKANFTVRHNVEDGSV QLADHYQQNTPIGDGPVLLPDNHYLSTQTVLSKDPNEK
**hEpoR-GFP1-10**
MDHLGASLWPQVGSLCLLLAGAAWAPPPNLYPYDVPDYAPDPKFESKAALLAARGPEELLCFTERLE DLVCFWEEAASAGVGPGNYSFSYQLEDEPWKLCRLHQAPTARGAVRFWCSLPTADTSSFVPLELRVT AASGAPRYHRVIHINEVVLLDAPVGLVARLADESGHVVLRWLPPPETPMTSHIRYEVDVSAGNGAGSV QRVEILEGRTECVLSNLRGRTRYTFAVRARMAEPSFGGFWSAWSEPVSLLTPSDLDPLILTLSLILVVIL VLLTVLALLSHRRALKQKIWGGSGGGGSGGMVSKGEELFTGVVPILVELDGDVNGHKFSVRGEGEGD ATIGKLTLKFICTTGKLPVPWPTLVTTLTYGVQCFSRYPDHMKRHDFFKSAMPEGYVQERTISFKDDGK YKTRAVVKFEGDTLVNRIELKGTDFKEDGNILGHKLEYNFNSHNVYITADKQKNGIKANFTVRHNVEDG SVQLADHYQQNTPIGDGPVLLPDNHYLSTQTVLSKDPNEK
**CHAMP-10-GPF11 (was not expressed)**
MDYKDDDDKGGGSLFLLLLSLLVMLLGLLLTILGLLFWGSGGGGRDHMVLHEYVNAAGIT
**GPF11-CHAMP-1**
MDYKDDDDKGGGRDHMVLHEYVNAAGITGGGSLFLLLLSLLVMLLGLLLTILGLLFWGSG

Supplementary Table 2. TM peptide sequences used in this work
Synthesized peptides were produced by solid-phase fmoc synthesis as C-terminal carboxamides and N-terminal free amines. "Biotin nCys mEpoR" was produced with biotin conjugated by peptide bond to the amino terminus. Fluorophore labeled TM peptides for FRET were produced as cysteine-maleimido conjugates at residues designated by asterisks. mEpoR-TM constructs for NMR were recombinantly expressed as fusion proteins: mEpoR-TM1 fused to T4 Lysozyme and cleaved by thrombin enzyme; mEpoR-TM2 fused to SUMO and cleaved chemically by Ni^{2+}-induced SNAC self-cleavage.

Thiol-disulfide	
nCys mEpoR	NH3-WKK**C**GGGPLILTL**S**LILVLI**S**LLLTVL**A**LLSHRR-NH2
cCys CHAMP-1	NH3-SWGR**S**LFLLLL**S**LL**VM**LL**G**LLLTIL**G**LLKSGGG**C**-NH2
cCys CHAMP-2	NH3-SWGR**S**LFLLLL**S**LL**AA**LL**G**LLLTIL**G**LLKSGGG**C**-NH2
cCys no Design TM	NH3-SWGRLI**A**LFLVLL**A**LLAGLA**G**LLALIL**A**LKSGGG**C**-NH2
FRET, Cys-maleimido	
nCys mEpoR	NH3-WKK**C***GGGPLILTL**S**LILVLI**S**LLLTVL**A**LLSHRR-NH2
cCys CHAMP-1	NH3-SWGR**S**LFLLLL**S**LLVMLL**G**LLLTIL**G**LLKSGGG**C***-NH2
Thiol-disulfide biotin capture	
Biotin nCys mEpoR	Biotin-GWK**C**GGGPLILTL**S**LILVLI**S**LLLTVL**A**LLSHRR
cCys CHAMP-1 v2	NH3-KKQNRR**S**LWLLLL**S**LLVMLL**G**LLLTIL**G**LLKSGG**C**-NH2
nCys CHAMP-1	NH3-**C**GGSR**S**LWLLLL**S**LLVMLL**G**LLLTIL**G**LLK-NH2
NMR, construct pair 1	
mEpoR-TM1	GSCGQSDNDPLILTLSLILVLISLLLTVLALLSHRR
cCys CHAMP-1	NH3-SWGR**S**LFLLLL**S**LLVMLL**G**LLLTIL**G**LLKSGGG**C***-NH2
NMR, construct pair 2	
mEpoR-TM2	SHHWNDPLILTLSLILVLISLLLTVLALLSHRR
CHAMP-1 v3	NH3-β-RSLFLLLLSLLVMLLGLLLTILGLLKSGGC-NH2
	β = β-alanine

Supplementary Table 3. Fusion protein constructs for NMR
Constructs for isotopically enriched recombinant expression in *E. coli*. Final TM constructs after thrombin protease cleavage or sequence-specific nickel-assisted cleavage (SNAC) used for NMR are underlined.

Construct	Sequence
**7x His T4 Lysozyme (thrombin) mEpoR TM-1**	MGSSHHHHHHHGGSGRGSHMGNIFEMLRIDEGLRLKIYKDTEGYYTIGIGHLLTKSPSL<br>NAAKSELDKAIGRNTNGVITKDEAEKLFNQDVDAAVRGILRNAKLKPVYDSLDAVRRAALINMVFQM<br>GETGVAGFTNSLRMLQQKRWDEAAVNLAKSRWYNQTPNRAKRVITTFRTGTWDAYAAGGSGSTE<br>NGLVPRGSCGQSDNDPLILTLSLILVLISLLLTVLALLSHRR
**7x His SUMO (SNAC) mEpoR TM-2**	MHHHHHHGSDSEVNQEAKPEVKPEVKPETHINLKVSDGSSEIFFKIKKTTPLRRL<br>MEAFAKRQGKEMDSLRFLYDGIRIQADQTPEDLDMEDNDIIEAHREQIGGAGG<br>SHHWNDPLILTLSLILVLISLLLTVLALLSHRR

Supplementary Table 4. CB chemical shift perturbations. Induced chemical shift changes of mEpoR-TM in ^{2}H-DPC upon addition of unlabeled CHAMP-1 peptide at 7 mol eqv. and 600 mol eqv. DPC, across multiple spectra including polypeptide carbon beta (CB) atoms resonances: HNCB, CBCANH, CCcoNH, ^{1}H-^{13}C HSQC. Frequencies listed in ppm. Complete resonance list for the monomeric mEpoR-TM in DPC can be found in the BMRB repository.

**Monomer mEpoR-TM, HNCB**				**Complex, CBCANH**				
	C	N	H		C	N	H	
	w1	w2	w3		w1	w2	w3	δ Shift
I9CB-N-H	36.40	118.23	7.40					
L10CB-N-H	42.14	122.01	8.43	L10CB-T11N-H	42.04	114.89	7.95	0.10
T11CB-N-H	68.50	114.75	7.92	T11CB-L12N-H	68.18	119.57	8.22	0.32
L12CB-N-H	41.86	119.50	8.20	L12CB-S13N-H	41.82	114.64	8.27	0.03
S13CB-N-H	62.94	114.66	8.31					
L14CB-N-H	41.61	121.30	7.98	L14CB-I15N-H	41.64	118.34	8.11	0.03
I15CB-N-H	37.33	118.24	8.07					
L16CB-N-H	41.47	119.27	8.28	L16CB-V17N-H	41.39	120.92	8.35	0.09
V17CB-N-H	31.30	121.10	8.37	V17CB-L18N-H	31.32	120.05	8.44	0.03
L18CB-N-H	41.69	119.97	8.41	L18CB-I19N-H	41.82	117.78	8.70	0.13
I19CB-N-H	37.56	117.28	8.69	I19CB-S20N-H	37.65	116.61	8.15	0.10
S20CB-N-H	62.67	116.76	8.19	S20CB-L21N-H	62.79	124.29	8.52	0.12
L21CB-N-H	42.01	124.30	8.49					
L22CB-N-H	41.79	119.51	8.33					
L23CB-N-H	41.75	117.08	8.60					
T24CB-N-H	67.80	116.15	7.90	T24CB-V25N-H	67.85	120.46	8.28	0.05
V25CB-N-H	31.35	120.47	8.28					
L26CB-N-H	41.58	118.71	8.36	L26CB-A27N-H	41.61	121.88	8.49	0.02
A27CB-N-H	18.32	121.93	8.53	A27CB-L28N-H	18.42	118.39	8.31	0.10
L28CB-N-H	42.26	118.43	8.35	L28CB-L29N-H	42.26	117.85	8.44	0.01
L29CB-N-H	42.24	117.89	8.46					
S30CB-N-H	63.83	112.87	7.91	S30CB-H31N-H	63.89	118.29	7.83	0.05
H31CB-N-H	28.92	118.14	7.87					
R32CB-N-H	30.85	126.07	7.76					
R33CB-N-H	31.76	126.08	7.75					

Supplementary Table 4. CB chemical shift perturbations, continued. Induced chemical shift changes of mEpoR-TM in ^{2}H-DPC upon addition of unlabeled CHAMP-1 peptide at 7 mol eqv. and 600 mol eqv. DPC, across multiple spectra including polypeptide carbon beta (CB) atoms resonances: HNCB, CBCANH, CCcoNH, ^{1}H-^{13}C HSQC. Frequencies listed in ppm.

**Complex, 1H-13C**			**Complex, CC(co)NH**						**Composite**	
inferred from unbound resonance										
	w1	δ Shift		w1	w2	w3	δ Shift		avg δ Shift	Error
								I9CB		
								L10CB	0.10	
								T11CB	0.32	
								L12CB	0.03	
			S13CB-L14N-H	62.87	121.35	7.98	0.07	S13CB	0.07	
			L14CB-I15N-H	41.71	118.27	8.11	0.10	L14CB	0.06	0.07
I15CB-HB	37.38	0.05						I15CB	0.05	
			L16CB-V17N-H	41.37	120.95	8.37	0.11	L16CB	0.10	0.02
								V17CB	0.03	
								L18CB	0.13	
								I19CB	0.10	
			S20CB-L21N-H	62.80	124.29	8.53	0.13	S20CB	0.12	0.01
			L21CB-L22N-H	41.89	119.78	8.35	0.12	L21CB	0.12	
			L22CB-L23N-H	41.94	117.46	8.57	0.15	L22CB	0.15	
								L23CB	*NA*	
								T24CB	0.05	
								V25CB	*NA*	
			L26CB-A27N-H	41.38	121.99	8.52	0.20	L26CB	0.11	0.18
			A27CB-L28N-H	18.41	118.54	8.27	0.09	A27CB	0.10	0.01
			L28CB-L29N-H	42.24	117.77	8.46	0.01	L28CB	0.01	0.01
			L29CB-S30N-H	42.24	112.81	7.91	0.01	L29CB	0.01	
			S30CB-H31N-H	63.90	118.47	7.84	0.07	S30CB	0.06	0.02
H31CB-HB	29.00	0.08						H31CB	0.08	

			R31CB-R32N-H	28.96	120.66	7.88	1.90	R32CB	1.90	
			R32CB-R33N-H	30.81	126.06	7.77	0.95	R33CB	0.95	

Supplementary Table 5. ^{13}C Sidechain chemical shift perturbations. Detectable and unambiguous shift changes of mEpoR-TM in ^{2}H-DPC with unlabeled CHAMP-1 in CCcoNH and ^{1}H-^{13}C HSQC spectra beyond CB. Frequencies listed in ppm.

TOCSY CCcoNH									
monomer TM				Champ-1 / mEpoR-TM complex					
	C	N	NH		C	N	NH		TOCSY shift
Assignment	w1	w2	w3	Assignment	w1	w2	w3		
T11CG2-L12N-H	21.473	119.506	8.207	T11CG-L12N-H	21.485	119.743	8.208		0.012
I15CD-L16N-H	10.71	119.264	8.292	I15CD-L16N-H	13.504	119.187	8.274		2.794
I15CG2-L16N-H	17.417	119.264	8.292	I15CG2-L16N-H	17.667	119.25	8.275		0.25
V17CG1-L18N-H	23.234	119.977	8.419	V17CG1-L18N-H	23.33	120.144	8.449		0.096
V17CG2-L10N-H	21.045	119.974	8.419	V17CG2-L18N-H	21.833	120.119	8.449		0.788
I19CG2-N-S20H	17.347	116.755	8.196	I19CG2-S20N-H	17.157	116.632	8.175		0.19
I19CD1-S20N-H	10.48	116.77	8.196						
L21CG-L22N-H	26.595	119.536	8.339	L21CG-L22N-H	26.956	119.802	8.35		0.361
L22CG-L23N-H	26.909	117.099	8.614	L22CG-L23N-H	26.576	117.44	8.571		0.333
T24CG2-V25N-H	21.376	120.465	8.287	T24CG-V25N-H	21.506	120.476	8.29		0.13
V25CG1-L26N-H	23.201	118.717	8.365	V25CG1-L26N-H	23.555	118.849	8.363		0.354
V25CG2-L26N-H	21.916	118.735	8.365	V25CG2-L26N-H	21.915	118.839	8.366		0.001
A27CB-L28N-H	18.324	118.454	8.355	A27CB-L28N-H	18.41	118.535	8.266		0.086
L29CG-S30N-H	26.77	112.886	7.921	L29CG-S30N-H	26.183	112.774	7.908		0.587
R32CD-R33N-H	43.548	126.092	7.763	R32CD-R33N-H	43.585	126.06	7.766		0.037
R32CG-R33N-H	27.243	126.091	7.763	R32CG-R33N-H	27.283	126.076	7.767		0.04

1H-13C HSQC								
	C	H		C	N	δ shift (HSQC)	error w/ TOCSY	avg δ Shift
Assignment	w1	w2	Assignment	w1	w2			
I15CD-HD	13.086	0.748	I15CD-HD	13.505	0.767	0.419	2.38	1.397
I15CG2-HG2	17.297	0.835	I15CG2-HG2	17.637	0.887	0.34	0.09	0.295
V17CG1-HG1	23.349	1.029	V17CG1-HG1	23.402	1.033	0.053	0.04	0.0745
V17CG2-HG2	21.134	0.842	V17CG2-HG2	21.728	0.885	0.594	0.19	0.691
I19CG2-HG2	17.302	0.835	I19CG2-HG2	17.28	0.839	0.022	0.02	0.106
T24CG2-HG2	21.412	1.127	T24CG-HG	21.542	1.129	0.13	0	0.13
V25CG1-HG1	23.26	1.07	V25CG1-HG1	23.468	1.05	0.208	0.15	0.281
V25CG2-HG2	21.86	0.94	V25CG2-HG2	21.898	0.92	0.038	0.04	0.0195
