# Supplementary material for: De novo-designed transmembrane proteins bind and regulate a cytokine receptor
Source: Nat Chem Biol. 2024 Mar 13;20(6):751–60. doi: 10.1038/s41589-024-01562-z (PMC11142920; doi:10.1038/s41589-024-01562-z)
Supplement: Supplementary file 2 — Reporting Summary [file 41589_2024_1562_MOESM2_ESM.pdf]

nature portfolio

Corresponding author(s): marco mravic, Bill DeGrado, Dan DiMaio

Last updated by author(s): Jan 9, 2024

Reporting Summary

Nature Portfolio wishes to improve the reproducibility of the work that we publish. This form provides structure for consistency and transparency in reporting. For further information on Nature Portfolio policies, see our Editorial Policies and the Editorial Policy Checklist.

Statistics

For all statistical analyses, confirm that the following items are present in the figure legend, table legend, main text, or Methods section.

n/a	Confirmed	
☐	☒	The exact sample size (*n*) for each experimental group/condition, given as a discrete number and unit of measurement
☐	☒	A statement on whether measurements were taken from distinct samples or whether the same sample was measured repeatedly
☐	☒	The statistical test(s) used AND whether they are one- or two-sided<br>*Only common tests should be described solely by name; describe more complex techniques in the Methods section.*
☒	☐	A description of all covariates tested
☐	☒	A description of any assumptions or corrections, such as tests of normality and adjustment for multiple comparisons
☐	☒	A full description of the statistical parameters including central tendency (e.g. means) or other basic estimates (e.g. regression coefficient) AND variation (e.g. standard deviation) or associated estimates of uncertainty (e.g. confidence intervals)
☐	☒	For null hypothesis testing, the test statistic (e.g. *F*, *t*, *r*) with confidence intervals, effect sizes, degrees of freedom and *P* value noted<br>*Give P values as exact values whenever suitable.*
☒	☐	For Bayesian analysis, information on the choice of priors and Markov chain Monte Carlo settings
☒	☐	For hierarchical and complex designs, identification of the appropriate level for tests and full reporting of outcomes
☒	☐	Estimates of effect sizes (e.g. Cohen's *d*, Pearson's *r*), indicating how they were calculated

Our web collection on statistics for biologists contains articles on many of the points above.

Software and code

Policy information about availability of computer code

Data collection	Rosetta3, ESMfold, Coiled-Coil Crick Parameterization, TopSpin v4, Agilent ChemStation Software, BioRad Chemidock MP imager. Custom software and Rosetta configuration files publicly available at https://github.com/mmravic314/CHAMP2023/
Data analysis	NMRPipe v9.9, FlowJo v10, Sparky-NMRFAM, Prism 9. Custom scripts provided in https://github.com/mmravic314/CHAMP2023/

For manuscripts utilizing custom algorithms or software that are central to the research but not yet described in published literature, software must be made available to editors and reviewers. We strongly encourage code deposition in a community repository (e.g. GitHub). See the Nature Portfolio guidelines for submitting code & software for further information.

Data

Policy information about availability of data

All manuscripts must include a data availability statement. This statement should provide the following information, where applicable:

- Accession codes, unique identifiers, or web links for publicly available datasets
- A description of any restrictions on data availability
- For clinical datasets or third party data, please ensure that the statement adheres to our policy

Chemical shift data has been uploaded to BMRB, with entry assigned accession number 51401.

Human research participants

Policy information about studies involving human research participants and Sex and Gender in Research.

Reporting on sex and gender	n/a
Population characteristics	n/a
Recruitment	n/a
Ethics oversight	n/a

Note that full information on the approval of the study protocol must also be provided in the manuscript.

Field-specific reporting

Please select the one below that is the best fit for your research. If you are not sure, read the appropriate sections before making your selection.

☒ Life sciences ☐ Behavioural & social sciences ☐ Ecological, evolutionary & environmental sciences

For a reference copy of the document with all sections, see nature.com/documents/nr-reporting-summary-flat.pdf

Life sciences study design

All studies must disclose on these points even when the disclosure is negative.

Sample size	n >=3. For cell-based assays, at least 3 independent cell culture biological replicates were performed; rationale for sufficient sample size is standard for subsequent t-test to assessing variance. More than 3 replicates for some samples were performed if that sample was repeated as internal comparison to other samples on different days. Figures present some as data representative of 3 independent replicate experiments (i.e. gels, HPLC chromatogram, etc)
Data exclusions	No data were excluded from the analysis
Replication	n=3, biological replicates for cell culture experiments; n=3 technical replicates for in vitro experiments. All attempts a replication were successful
Randomization	Stable transduced cell lines were prepared for cell culture experiments. For each data point replicate, cells derivative from a parent cell line were cultured independently and seeded randomly into respective well plates to be assayed for n=3 biological replicates on different days.
Blinding	Not applicable; investigators were not blinded to group allocations during data collection and analysis. All experiments described in the manuscript require the samples to be clearly labeled for data analysis to be feasibly performed by the respective researchers

Reporting for specific materials, systems and methods

We require information from authors about some types of materials, experimental systems and methods used in many studies. Here, indicate whether each material, system or method listed is relevant to your study. If you are not sure if a list item applies to your research, read the appropriate section before selecting a response.

Materials & experimental systems

n/a	Involved in the study	
☐	☒	Antibodies
☐	☒	Eukaryotic cell lines
☒	☐	Palaeontology and archaeology
☒	☐	Animals and other organisms
☒	☐	Clinical data
☒	☐	Dual use research of concern

Methods

n/a	Involved in the study	
☒	☐	ChIP-seq
☐	☒	Flow cytometry
☒	☐	MRI-based neuroimaging

Antibodies

Antibodies used	Secondary: donkey anti-mouse HRP (Jackson Immunoresearch); donkey anti-rabbit HRP (Jackson Immunoresearch). Primary or conjugated: HRP-conjugated mouse anti-HA (clone 6E2, Cell Signaling). Anti-JAK2 (clone D2E12, Cell Signaling). Anti-STAT5

	#9363 (Cell Signaling). Anti-phospho-JAK2 (Tyr1008) (clone D4A8, Cell Signaling). Anti-phospho-STAT5 (Y694) #9351 (Cell Signaling). Anti-FLAG (M2)(Sigma-Aldrich). Anti-EPOR (clone C-20 sc-695, Santa Cruz). anti-PDGF (rabbit polyclonal antiserum, raised against the C-terminal 13 amino acids of the human PDGFβR)
Validation	All the commercial antibodies were validated by the vendors, and were used as instructed. The in-house raised anti-PDGF antibody was validated in previous reports (Petti, Lisa M., et al. "A single amino acid substitution converts a transmembrane protein activator of the platelet-derived growth factor β receptor into an inhibitor." Journal of Biological Chemistry 288.38 (2013): 27273-27286.)

Eukaryotic cell lines

Policy information about cell lines and Sex and Gender in Research

Cell line source(s)	HEK293T cell line: Human embryonic kidney 293 cells with SV40 large T antigen. Source: HEK293T/17 (293T) cells used for making retroviruses were purchased from ATCC.<br>BaF3 cell line: murine interleukin-3 dependent pro-B cell line. Source: Alan D'Andrea, Dana Farber Cancer Institute.
Authentication	Upon thawing, each aliquot of BaF3 cells were tested for characterisic morphology, growth in suspension, and IL-3 dependence . Expression of transferred genes (e.g. EPOR) was verified by western blotting. The identity of HEK 293T cells was authenticated by the American Type Culture Collection cell line authentication service, and authenticated by the supplier by STR profiling
Mycoplasma contamination	293T and BaF3 cells were mycoplasma free (MycoAlert Mycoplasma Detection Kit, Lonza)
Commonly misidentified lines<br>(See ICLAC register)	None

Flow Cytometry

Plots

Confirm that:

☒ The axis labels state the marker and fluorochrome used (e.g. CD4-FITC).

☒ The axis scales are clearly visible. Include numbers along axes only for bottom left plot of group (a 'group' is an analysis of identical markers).

☒ All plots are contour plots with outliers or pseudocolor plots.

☒ A numerical value for number of cells or percentage (with statistics) is provided.

Methodology

Sample preparation	Cells were collected by centrifugation at 1,000 rpm for 10 minutes at 4°C, washed in cold PBS, and re-suspended in 300ul cold PBS.
Instrument	CytoFlex LX
Software	FlowJo V10
Cell population abundance	20,000 events were collected for each sample. After gating, 10,000 - 16,000 events were used for figures.
Gating strategy	The default FSC/SSC gating was used. Events with FSC values lower than a threshold were not recorded. A mock sample was used in every trial to ensure that the main cell population is recorded.

☒ Tick this box to confirm that a figure exemplifying the gating strategy is provided in the Supplementary Information.
