# Supplementary figures and images for: De novo-designed transmembrane proteins bind and regulate a cytokine receptor
Source: Nat Chem Biol. 2024 Mar 13;20(6):751–60. doi: 10.1038/s41589-024-01562-z (PMC11142920; doi:10.1038/s41589-024-01562-z)

- Fig. 1 Unprocessed gels

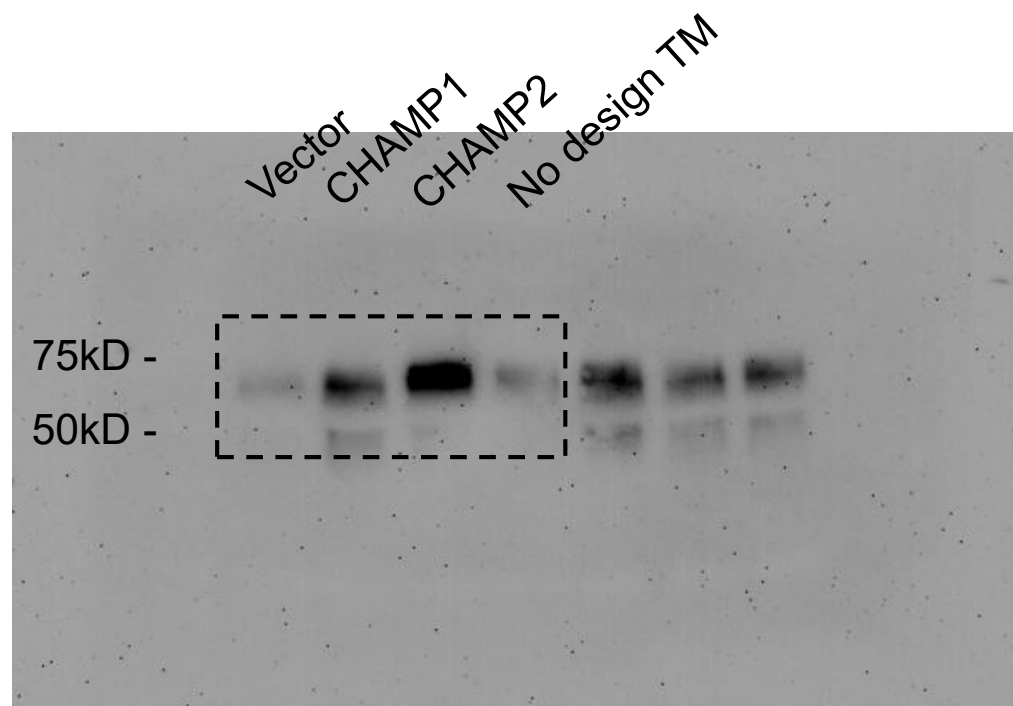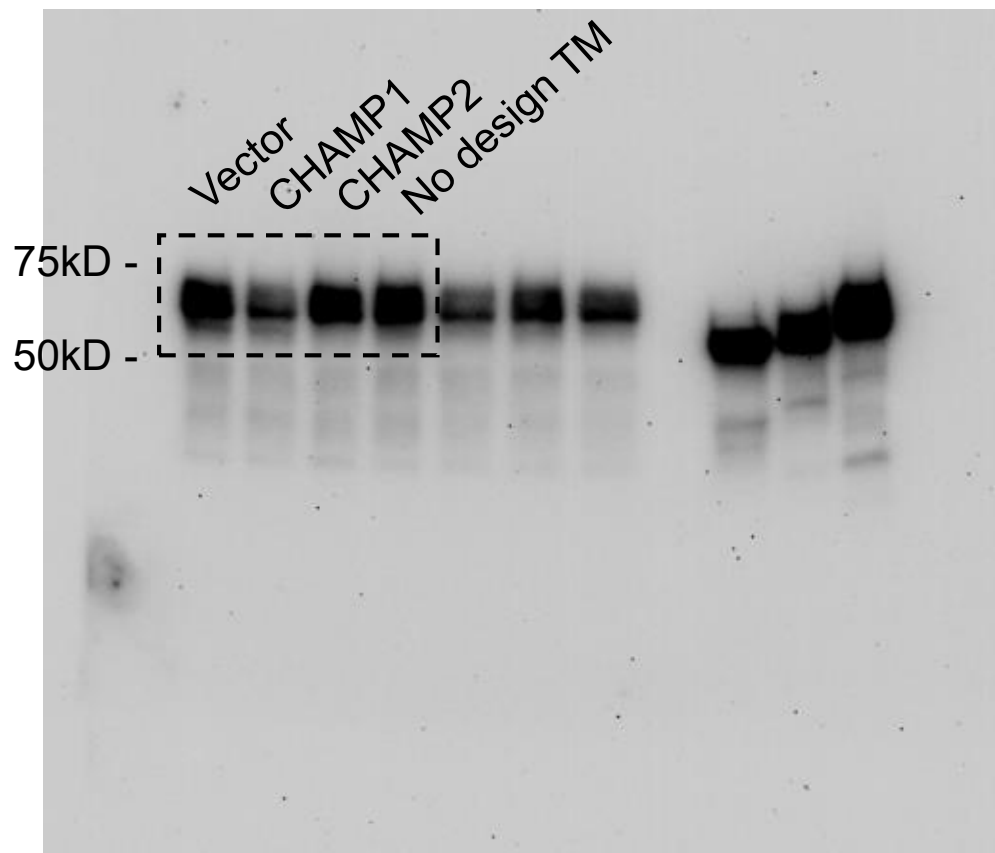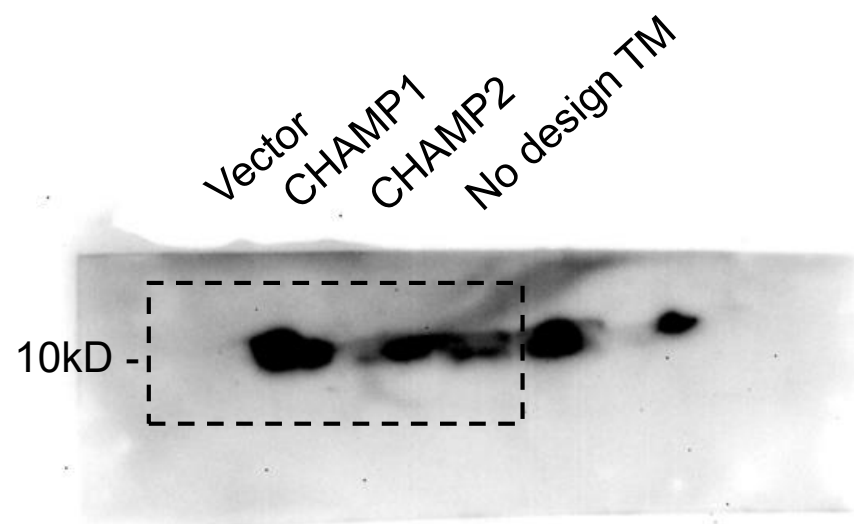

Supplement: Supplementary file 3 — Unprocessed western blots. [file 41589_2024_1562_MOESM3_ESM.pdf]

• Fig. 3 Unprocessed gels

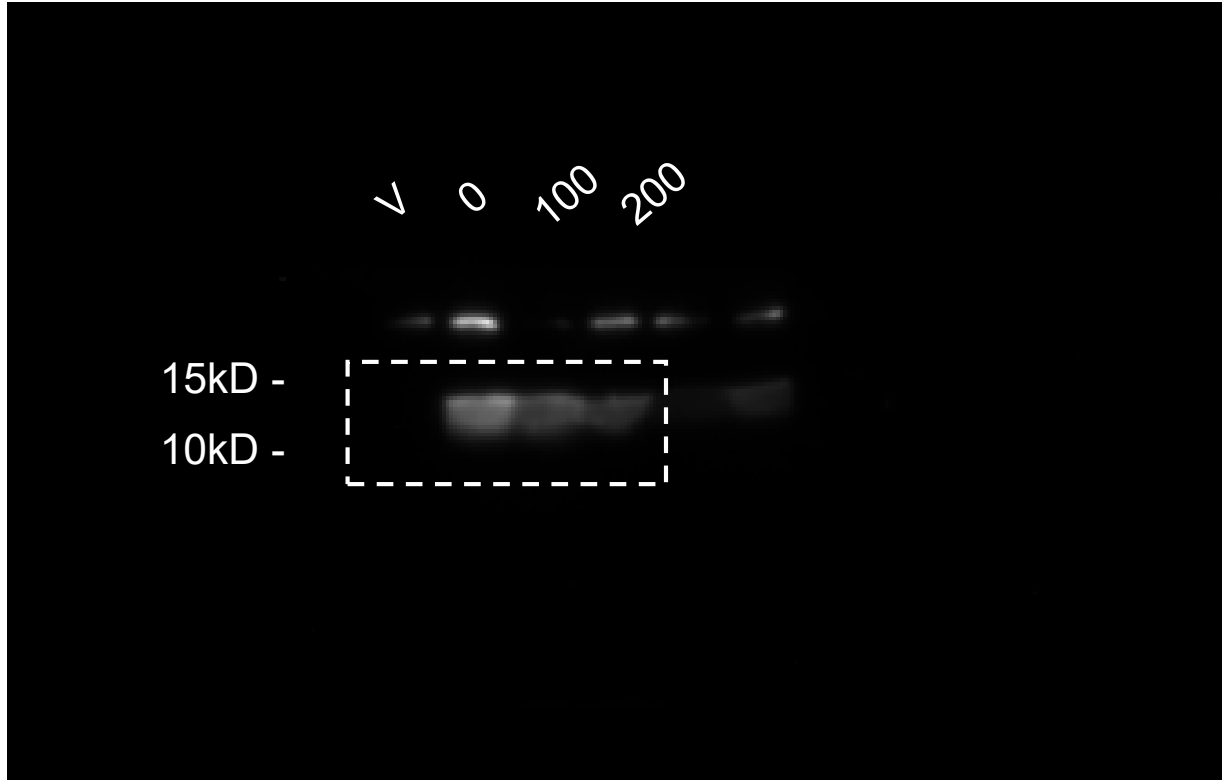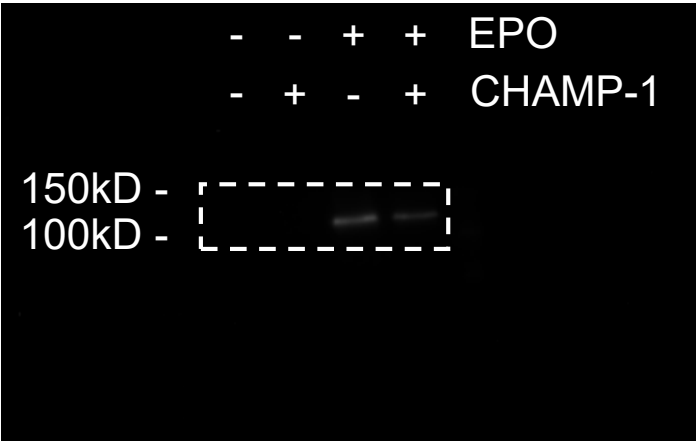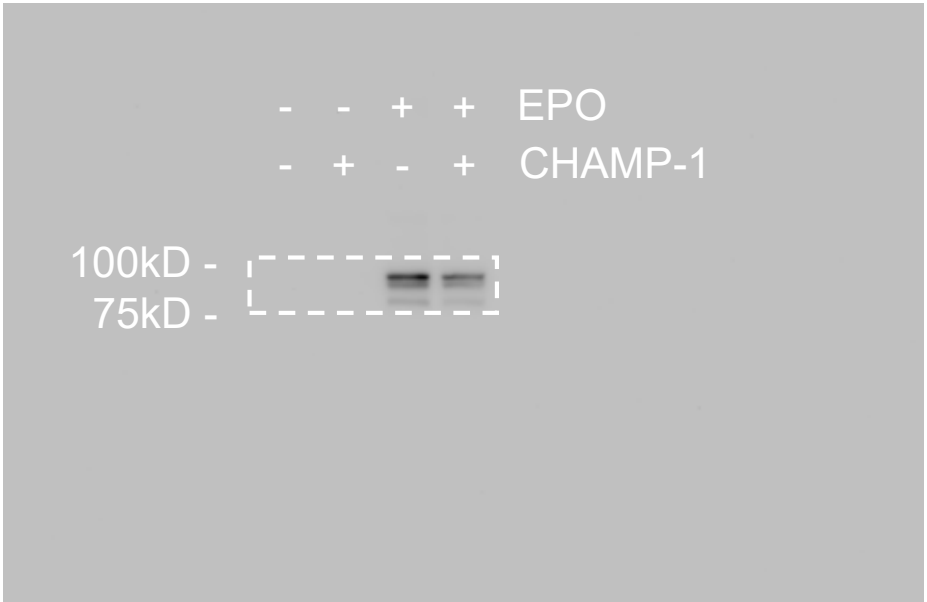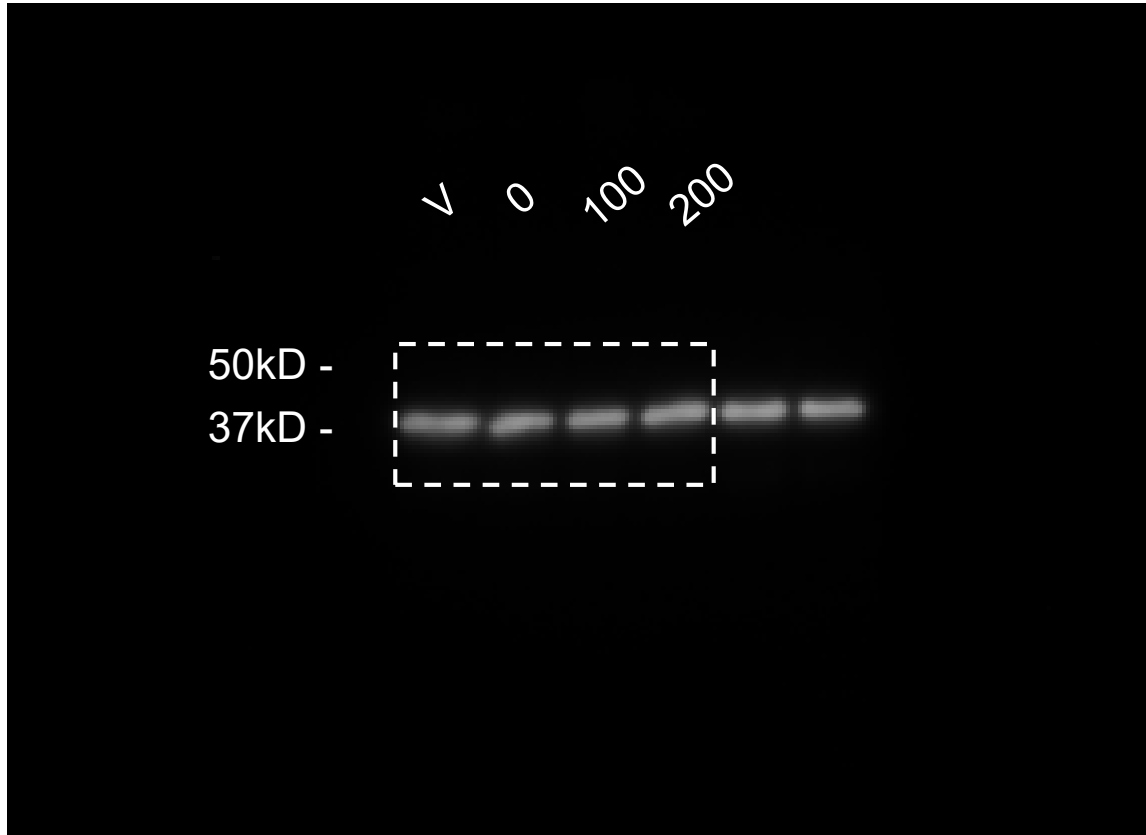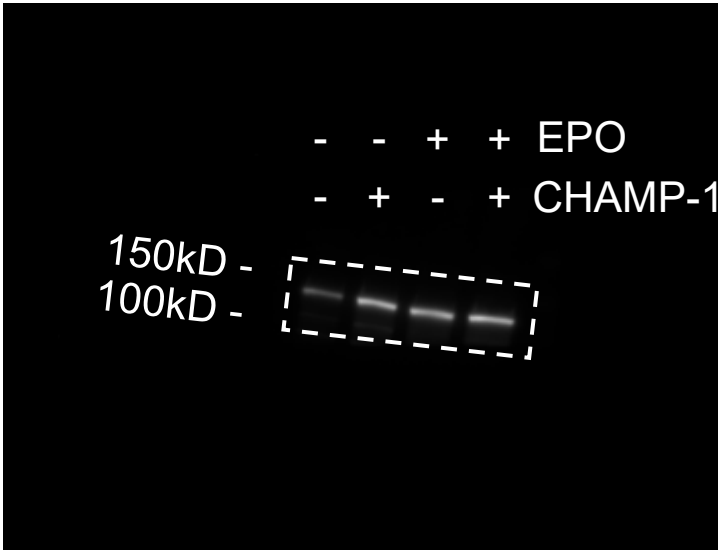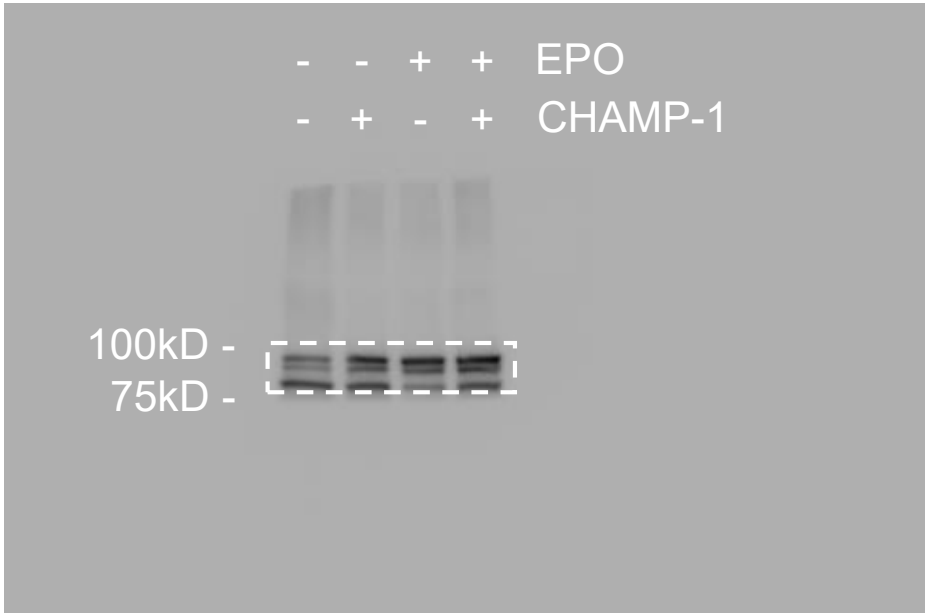

Supplement: Supplementary file 4 — Unprocessed western blots. [file 41589_2024_1562_MOESM4_ESM.pdf]

Extended Data Figure 3c

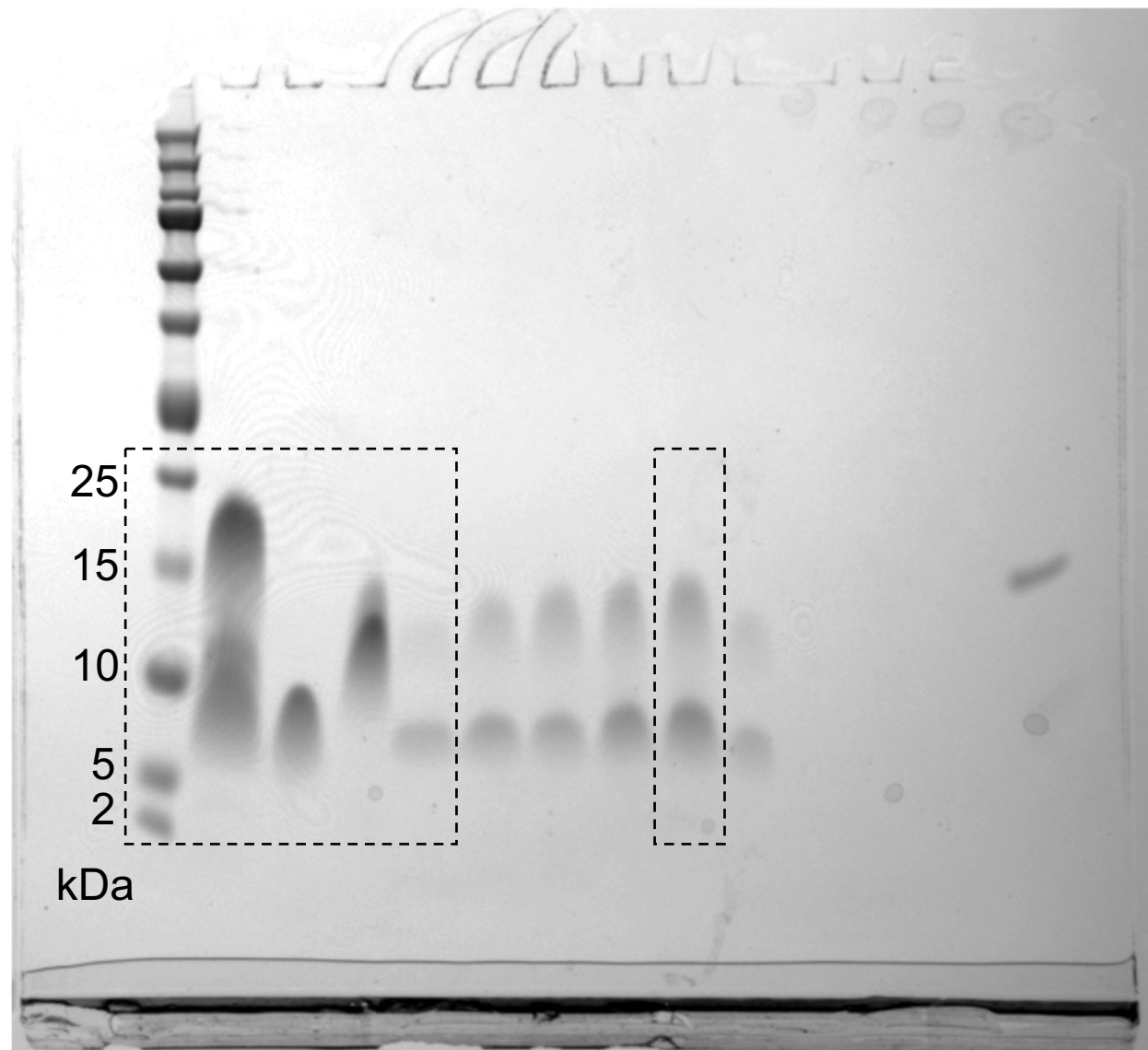

Supplement: Supplementary file 6 — Unprocessed gel. [file 41589_2024_1562_MOESM6_ESM.pdf]

- Extended Fig. S4 Unprocessed gels

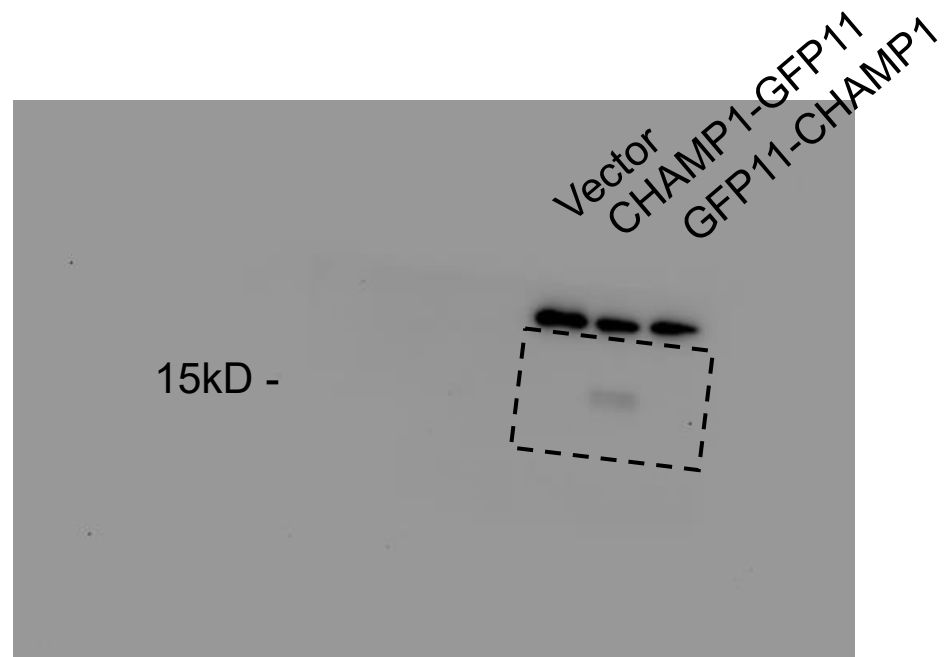

Vector  
CHAMP1-GFP11  
GFP11-CHAMP1

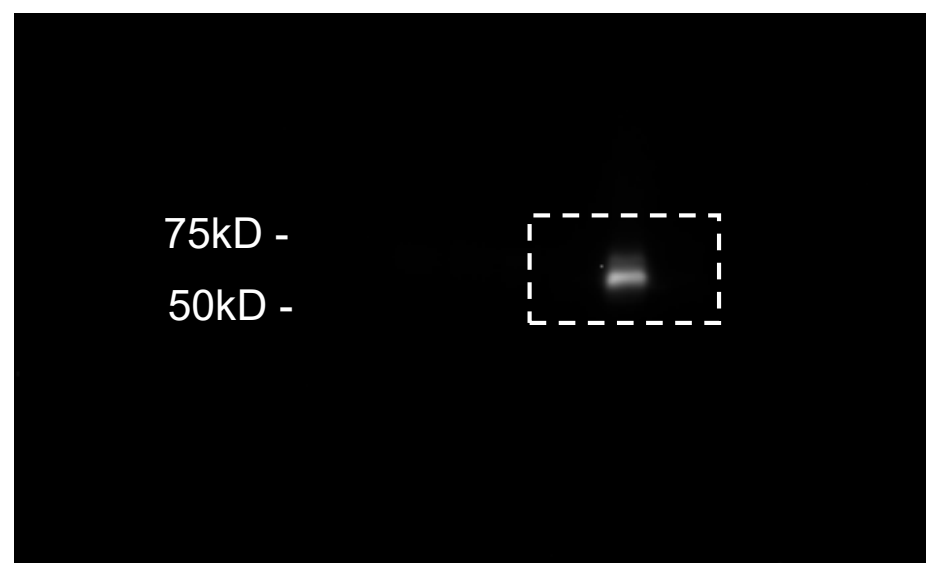

Vector  
CHAMP1-GFP11  
GFP11-CHAMP1

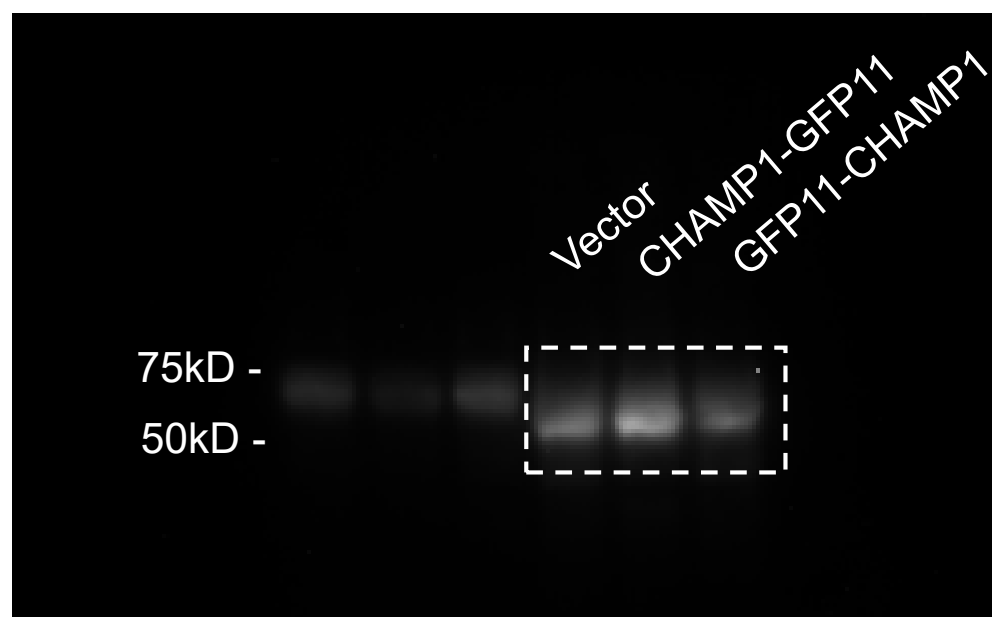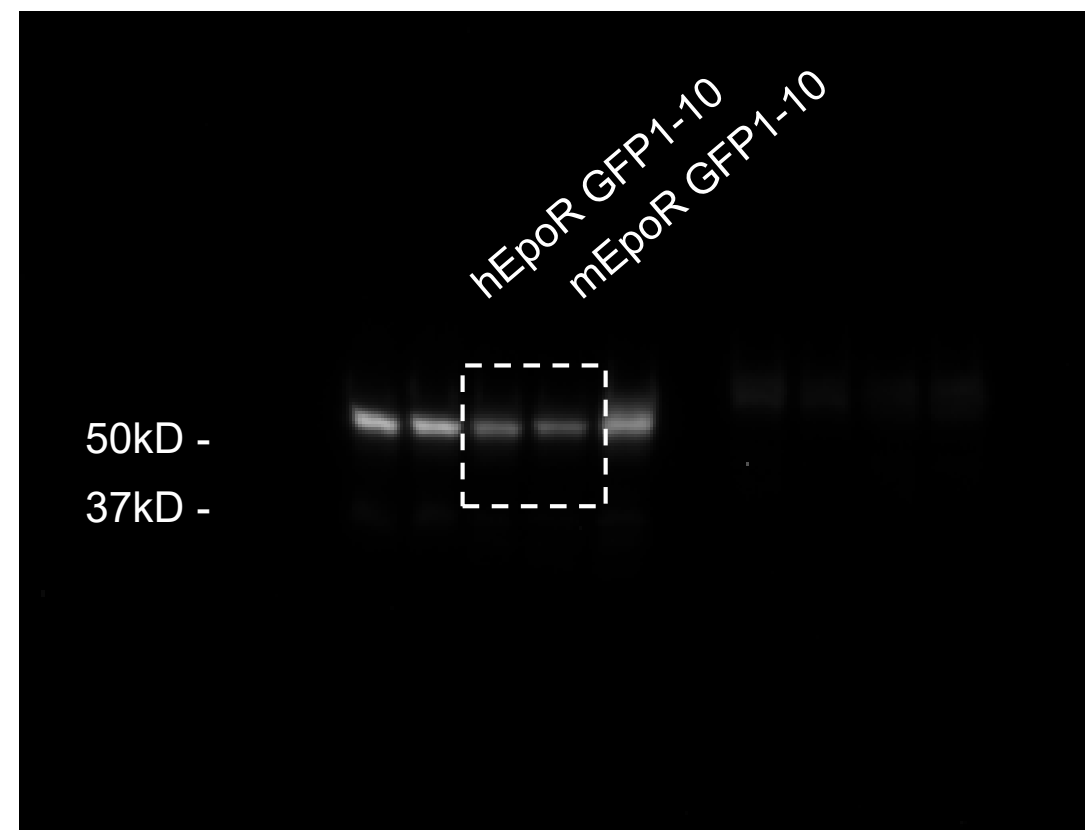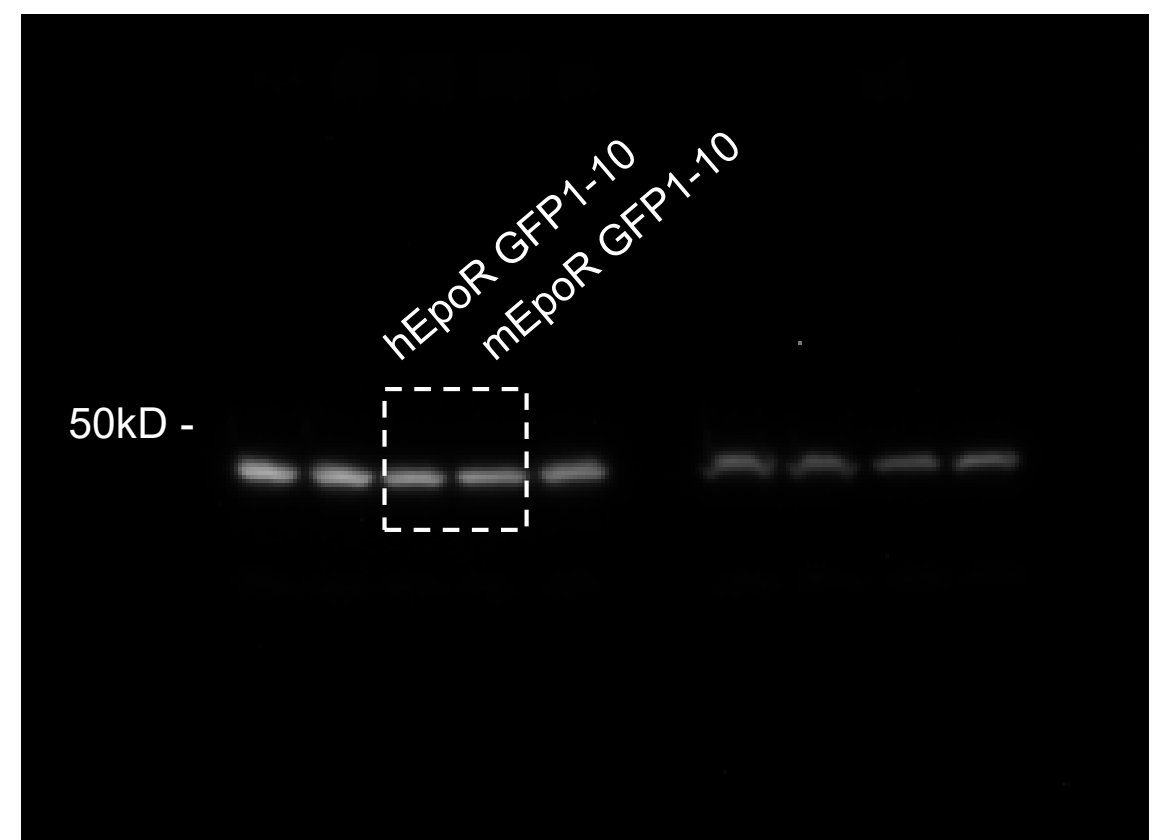

Supplement: Supplementary file 7 — Unprocessed western blots. [file 41589_2024_1562_MOESM7_ESM.pdf]

- Extended Fig. S6 Unprocessed gels

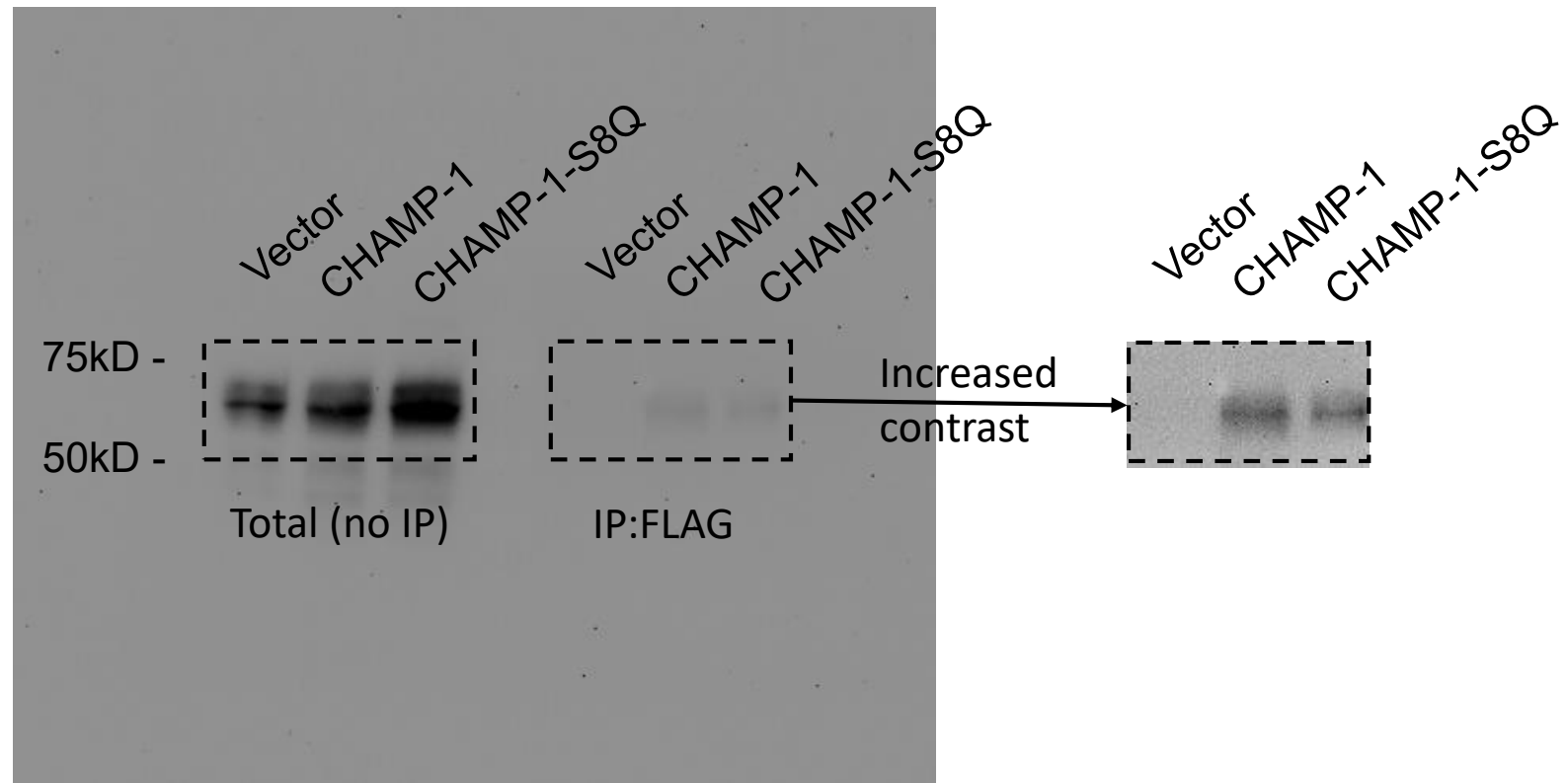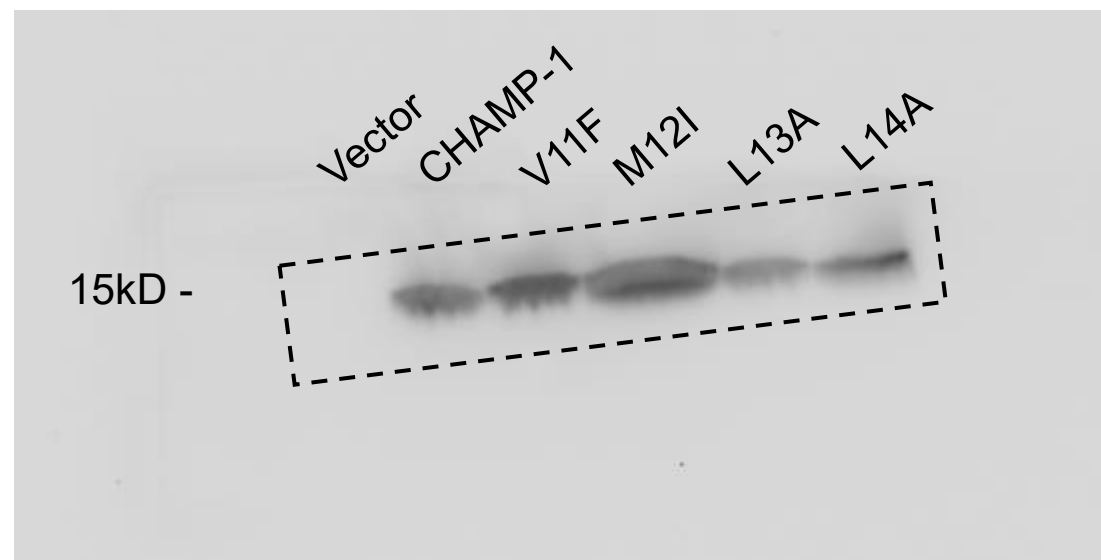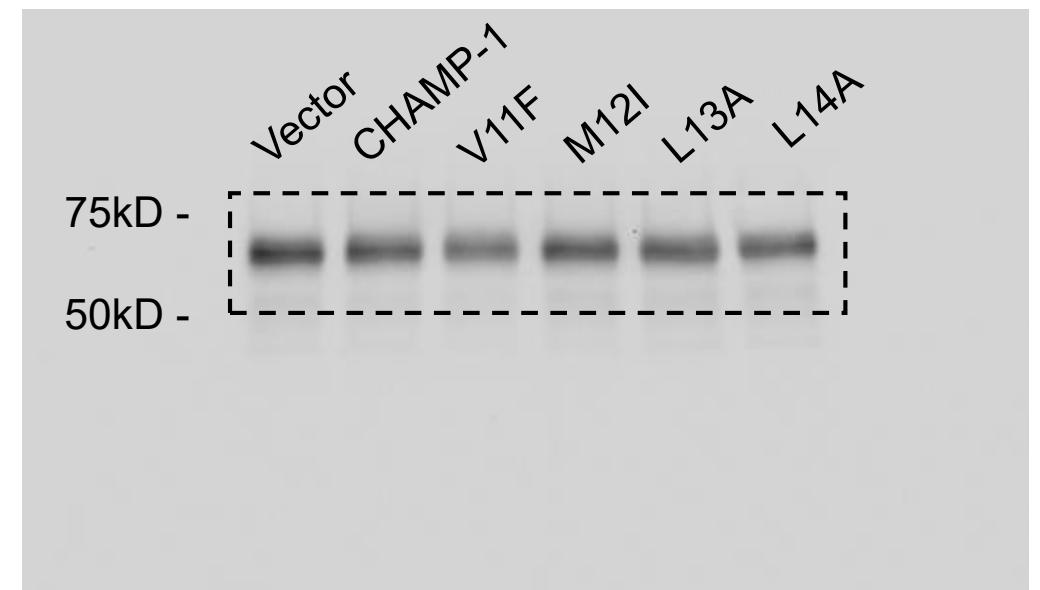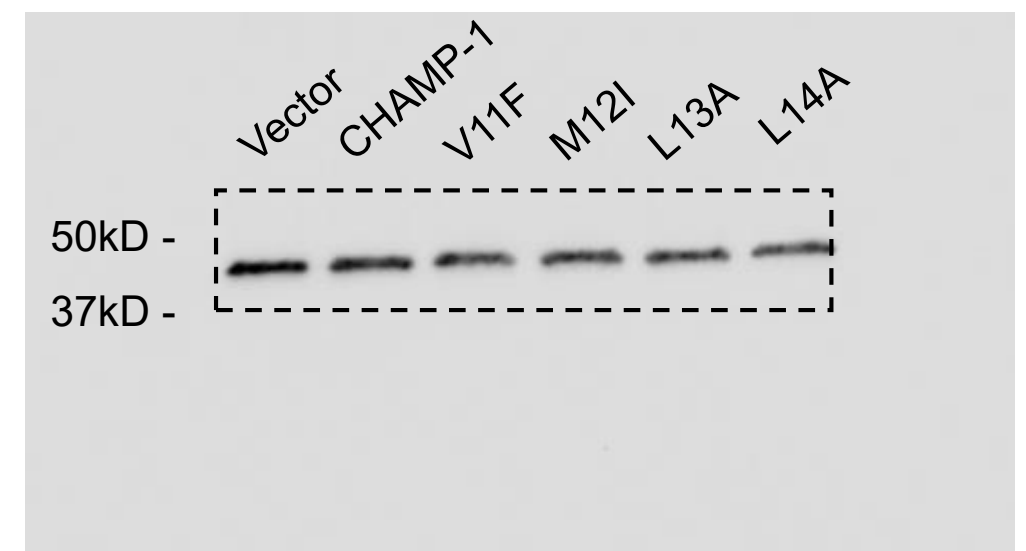

Supplement: Supplementary file 8 — Unprocessed western blots. [file 41589_2024_1562_MOESM8_ESM.pdf]

Extended Data Figure 9b

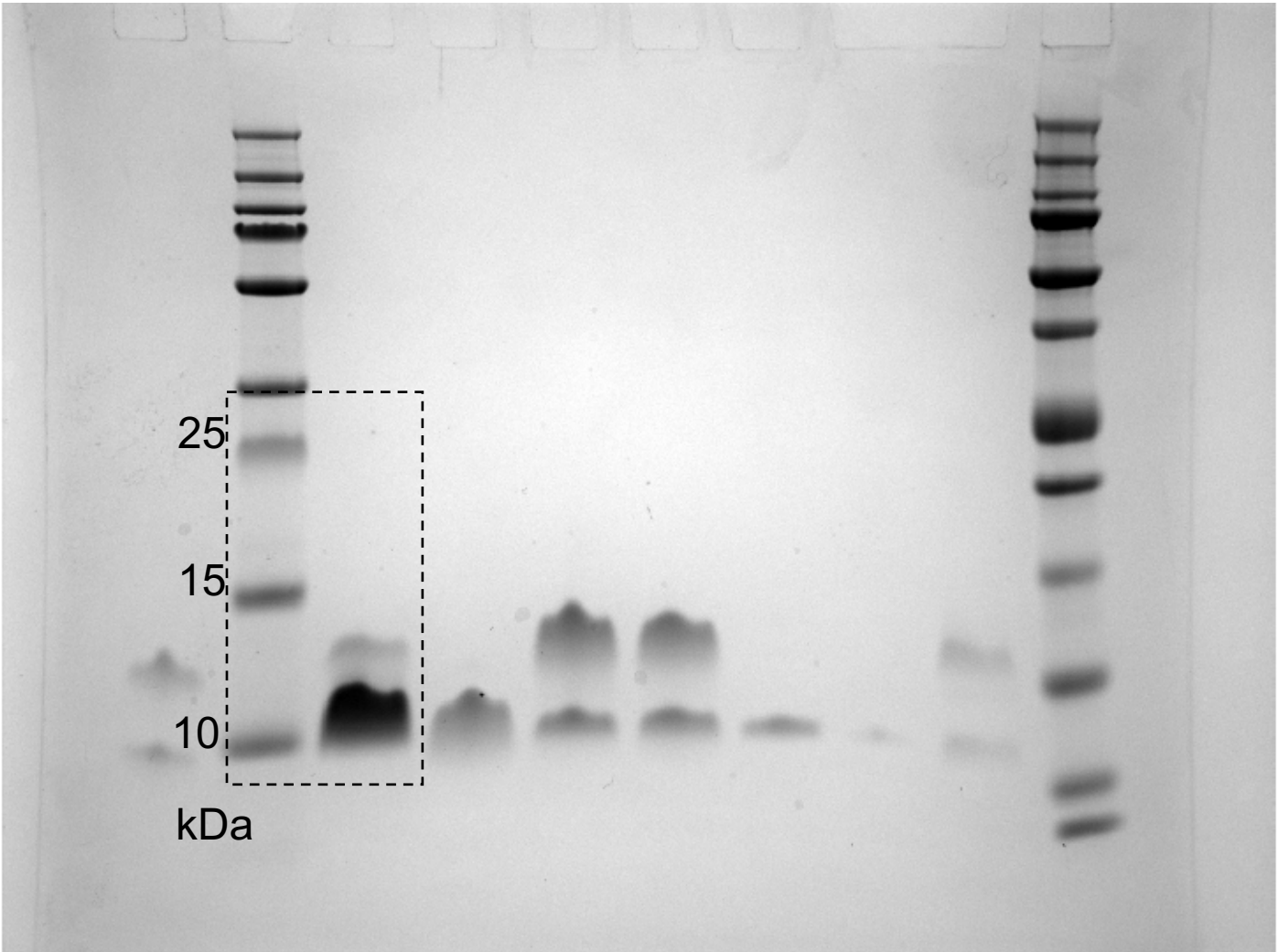

Supplement: Supplementary file 9 — Unprocessed gel. [file 41589_2024_1562_MOESM9_ESM.pdf]
