# Supplementary material for: De novo-designed transmembrane proteins bind and regulate a cytokine receptor
Source: Nat Chem Biol. 2024 Mar 13;20(6):751–60. doi: 10.1038/s41589-024-01562-z (PMC11142920; doi:10.1038/s41589-024-01562-z)

- Extended Fig. S2 Unprocessed gels

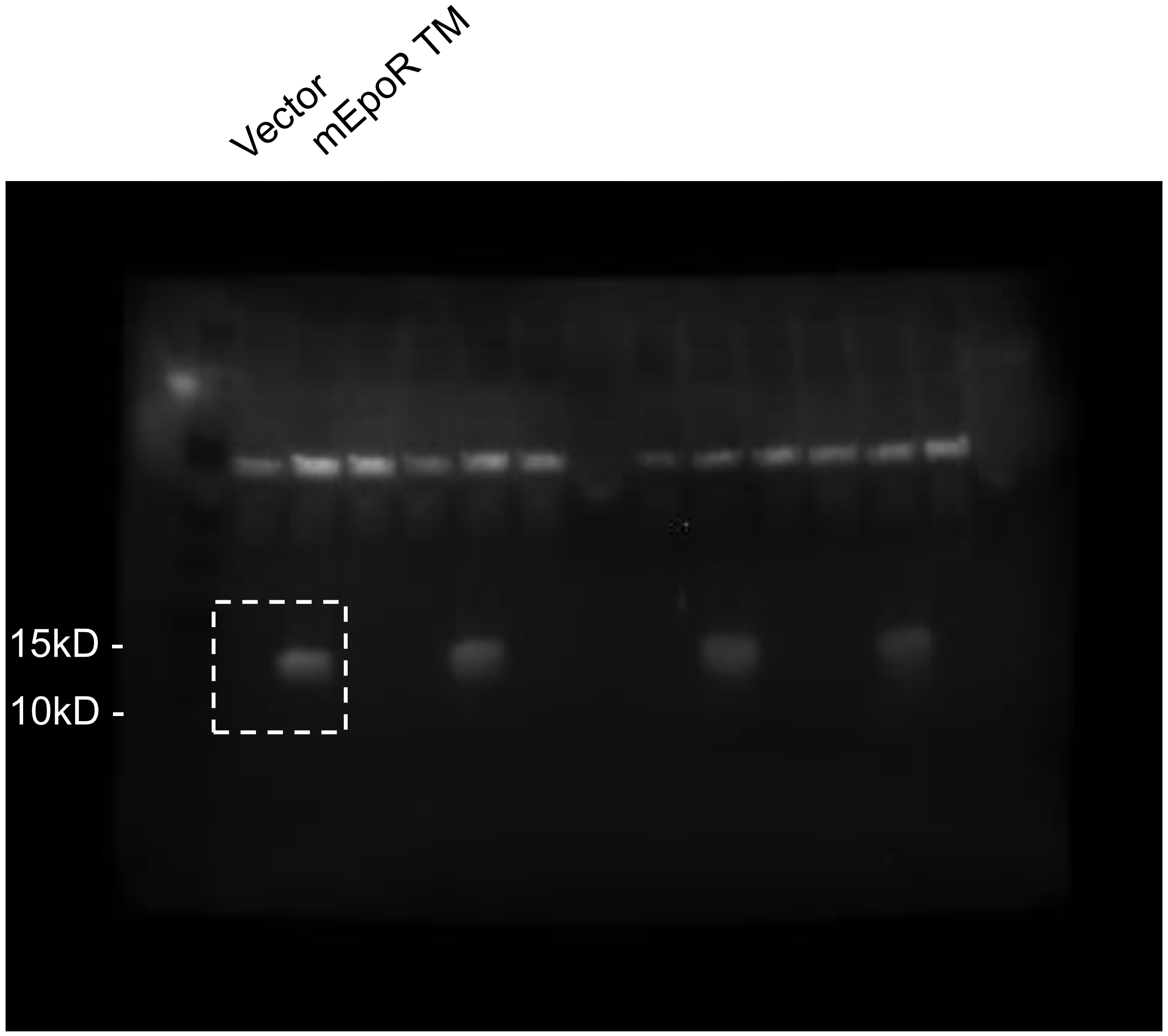

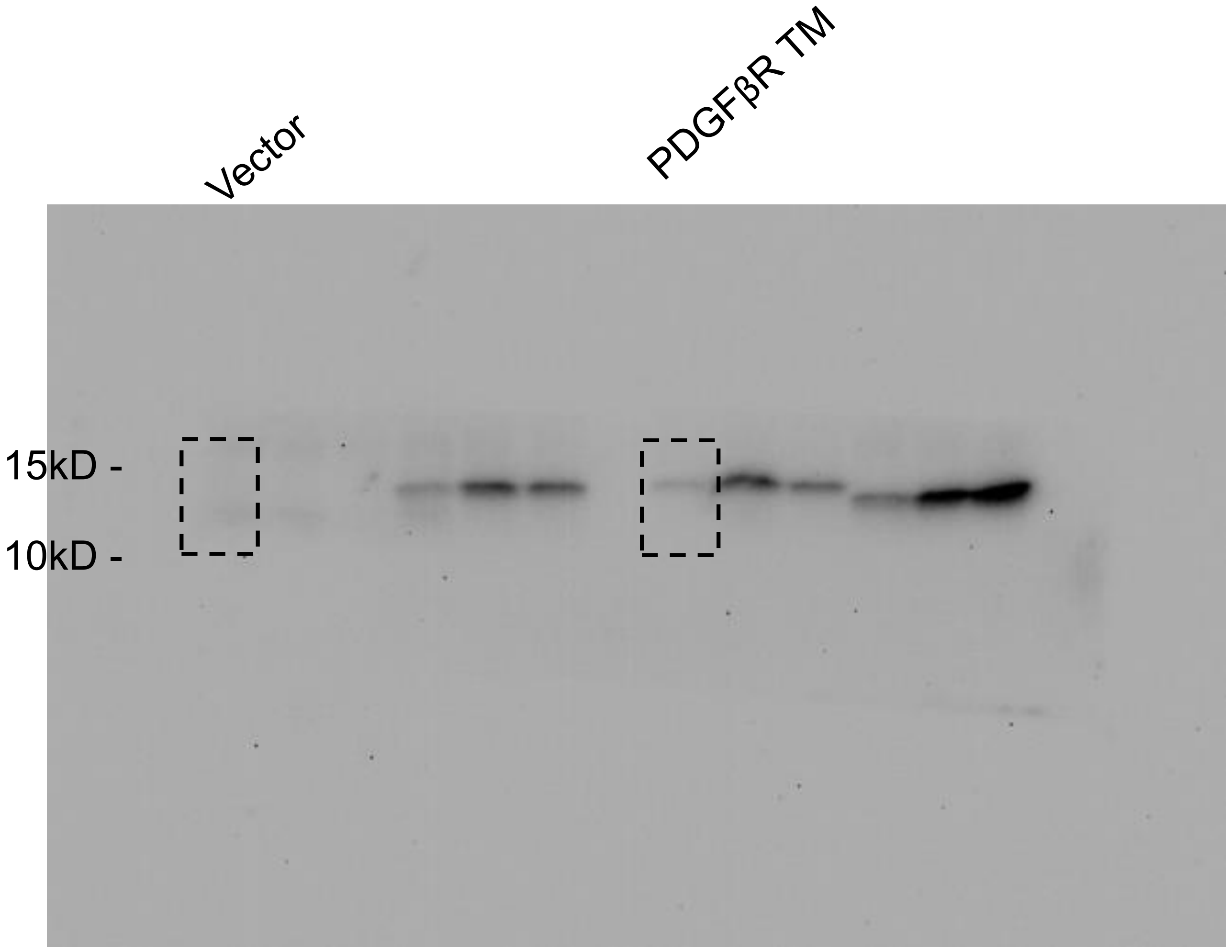

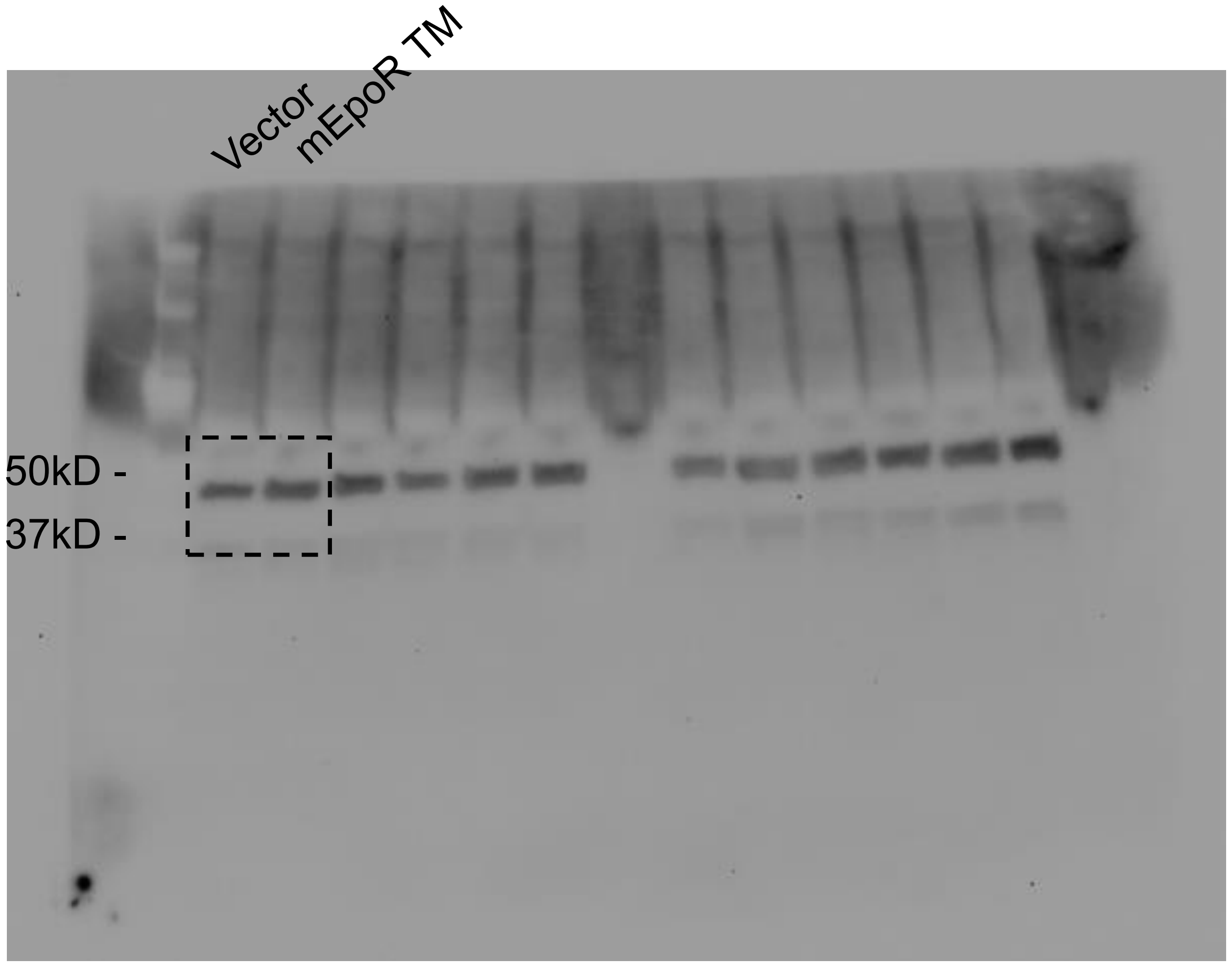

Same blot (loading control)

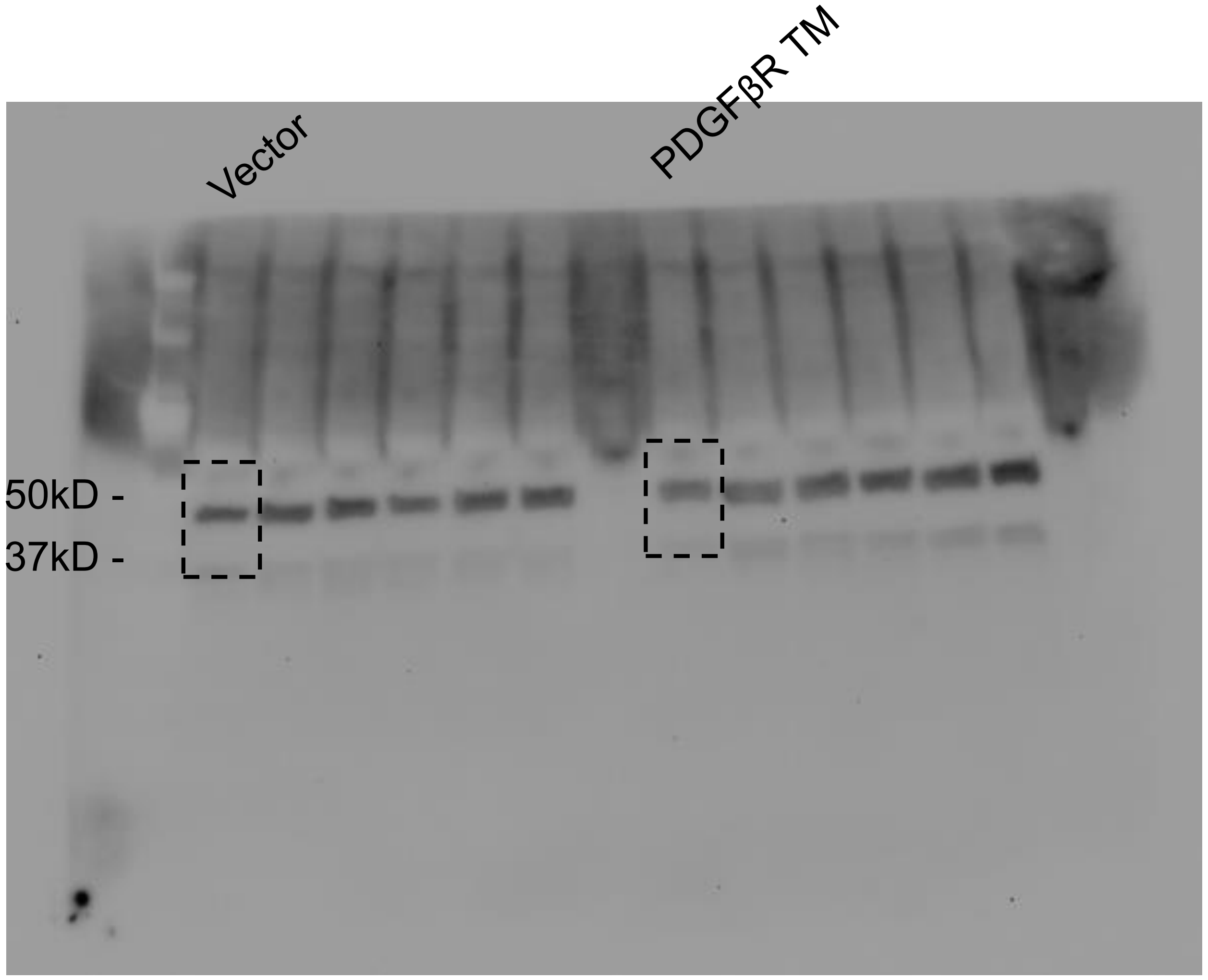

Supplement: Supplementary file 5 — Unprocessed western blots. [file 41589_2024_1562_MOESM5_ESM.pdf]
